# Collagen Sequence Analysis of the Extinct Giant Ground Sloths *Lestodon* and *Megatherium*

**DOI:** 10.1371/journal.pone.0139611

**Published:** 2015-11-05

**Authors:** Michael Buckley, Richard A. Fariña, Craig Lawless, P. Sebastián Tambusso, Luciano Varela, Alfredo A. Carlini, Jaime E. Powell, Jorge G. Martinez

**Affiliations:** 1 Faculty of Life Sciences, Manchester Interdisciplinary Biocentre, The University of Manchester, Manchester, United Kingdom; 2 Sección Paleontología, Facultad de Ciencias, Universidad de la República, Iguá 4225, Montevideo, Uruguay; 3 Michael Smith Building, The University of Manchester, Manchester, United Kingdom; 4 Facultad de Ciencias Naturales y Museo, Museo de La Plata, Buenos Aires, Argentina; 5 Facultad de Ciencias Naturales, Universidad Nacional de Tucumán, Tucumán Province, Argentina; 6 Instituto Superior de Estudios Sociales-CONICET, Instituto de Arqueología y Museo-UNT, Tucumán Province, Argentina; BiK-F Biodiversity and Climate Research Center, GERMANY

## Abstract

For over 200 years, fossils of bizarre extinct creatures have been described from the Americas that have ranged from giant ground sloths to the ‘native’ South American ungulates, groups of mammals that evolved in relative isolation on South America. Ground sloths belong to the South American xenarthrans, a group with modern although morphologically and ecologically very different representatives (anteaters, armadillos and sloths), which has been proposed to be one of the four main eutherian clades. Recently, proteomics analyses of bone collagen have recently been used to yield a molecular phylogeny for a range of mammals including the unusual ‘Malagasy aardvark’ shown to be most closely related to the afrotherian tenrecs, and the south American ungulates supporting their morphological association with condylarths. However, proteomics results generate partial sequence information that could impact upon the phylogenetic placement that has not been appropriately tested. For comparison, this paper examines the phylogenetic potential of proteomics-based sequencing through the analysis of collagen extracted from two extinct giant ground sloths, *Lestodon* and *Megatherium*. The ground sloths were placed as sister taxa to extant sloths, but with a closer relationship between *Lestodon* and the extant sloths than the basal *Megatherium*. These results highlight that proteomics methods could yield plausible phylogenies that share similarities with other methods, but have the potential to be more useful in fossils beyond the limits of ancient DNA survival.

## Introduction

In the last few decades, the impressive South American megafauna has been the subject of an increasing research effort [1]. Among its several appeals, its taxonomic composition is highly peculiar, with many giant xenarthrans across a range of localities. With only about 30 living, rather small species (21 armadillos, four tree sloths and six anteaters; [2]), this monophyletic group of placental mammals had an impressive richness throughout the South American Cenozoic [2–5]. They reached North America during the Great American Biotic Interchange (GABI; [6, 7]) and many of its species (especially those of large body size) went extinct near the Pleistocene—Holocene border [8]. According to the latest classification, xenarthrans, one of the four main placental clades, are the sister group of the remaining placentals, the Epitheria [9], although that node has proved unstable in recent molecular phylogenies (e.g., associated with Afrotheria to make up the Atlantogenata, [10]). Within the Xenarthra, with an origin of around 105 Ma [11, 12], two groups are recognized [3, 5]; one of them, Cingulata, consisting of the armadillos and their extinct relatives (glyptodonts and pampatheres) and the other, Pilosa, including the anteaters (Vermilingua) and the sloths (Tardigrada [2]–but see discussions on nomenclature in Fariña & Vizcaíno [13]).

While the cingulates, with their several modern species, have been the subject of molecular systematics [14], sloths, being so poorly represented in modern faunas, have been mostly classified based on morphological characteristics [15], although molecular phylogenies including extant sloths do exist and they are congruent with those based on morphological characters [16–18]. Moreover, it should be noted that ancient DNA (aDNA) has been described for the fur and faeces of the exceptionally well-preserved *Mylodon darwinii* found mummified in Última Esperanza, southern Chile [19] and *Nothrotheriops shastensis* in Gypsum Cave, USA [20]. Molecular results are congruent with morphological phylogenies [15] that place the modern three-toed sloth *Bradypus* as the sister group of the remaining sloths and the two-toed sloth *Choloepus* among the Megalonychidae. The Mylodontidae, the group that includes one of the species analysed here, *Lestodon armatus*, split in the first subsequent dichotomy from the Megatherioidea, which includes the other species dealt with in this paper, *Megatherium americanum*.

Although aDNA gives interesting results in terms of phylogeny and systematics, some proteins, another phylogenetically-informative class of biomolecules, especially collagen, are promissory as well [21, 22]. They survive in fossils for lengths of time that are an order of magnitude greater than for DNA [23] and have been investigated for the phylogenetic potential to resolve the relationships of extinct taxa for decades [24, 25]. Recent developments in protein sequence analysis enable complex mixtures of proteins (i.e., proteomes) to be routinely analysed using techniques of ‘soft-ionisation’ mass spectrometry. This technology now allows us the ability to obtain protein sequence information and infer evolutionary relationships from long extinct organisms much deeper into the past than previously thought possible.

Although the biomineralised tissue that is bone contains thousands of different proteins [26], most of these do not survive long periods of time within a burial environment, where a general qualitative decrease in proteome complexity with increasing chronological age has been observed [27]. However, the dominant protein of bone, type 1 collagen, has been demonstrated to survive much longer than other non-collagenous proteins [27, 28] and also, more importantly, in specimens that no longer yield aDNA [28]. Recent analyses unambiguously reporting the survival of collagen within Pliocene sub-fossil material ~3.5 Ma [29] demonstrate its potential for a wide range of extinct taxa. Although the use of ancient collagen to infer phylogenetic relationships of extinct taxa has been practised for several decades [22, 24], it is only relatively recently that collagen sequence information has been acquired using proteomics techniques. Although proteomics has the advantage that it can retrieve sequence information from complex mixtures of proteins and peptides [30, 31], because of its probability-matching nature, it is prone to false positive matches and potentially non-random sequence acquisition. Thus the sometimes highly-partial nature of the acquired sequences, which have recently been used to identify the ‘malagasy aardvark’ as a type of giant tenrec [22], and confirmation that the South American endemic ungulates derive from condylarths [32] needs further validation in light of new sequence information from other taxonomic groups.

The aims of this research are to evaluate the phylogenetic integrity of collagen sequencing by proteomics where sequence information of closely-related taxa is limited. Given that the xenarthrans remain the poorest studied of the four major mammalian groups, here we present results of the species *Megatherium americanum*, known since the last years of the 18^th^ century [33], and *Lestodon armatus*, described in the mid-19^th^ century [34].

## Materials and Methods

All permits were obtained to recover archaeological/palaeontological material where required. Protein extraction from two specimens of *Lestodon* (specimen numbers 474 and 975A; repository: held in the Colección del Arroyo del Vizcaíno, Sauce, Departamento de Canelones, Uruguay, no permits were required for the described study under Uruguayan law) from the Arroyo del Vizcaíno site, ca. 30 Ka; [35]) and two specimens of *Megatherium* (one ungual phalange from Penas de las Trampas 1.1, Argentina, ca 12.5 Ka [36] specimen code PVL 6786–1 held in the Colección Paleontología Vertebrados Lillo, Facultad de Ciencias Naturales e Instituto Miguel Lillo—Universidad Nacional de Tucumán, Miguel Lillo 205. (4000) San Miguel de Tucumán, Argentina under permits issued by the Dirección Provincial de Antropología-Secretaría de Estado de Catamarca, Argentina Resolución N° 098/11, and one from Las Chacras, Rio Negro, Argentina, ca Late Pleistocene; MAPB# 3965, a museum specimen at the Museo Arqueológico y Paleontológico de Bariloche, Río Negro, Argentina, dated to ca. 18 Ka) was carried out following methods described by Wadsworth & Buckley [27]. In brief, ~30–50 mg bone powder was decalcified with 0.6 M hydrochloric acid (HCl) for ~18 hours, and centrifuged (14,000 rpm) for 5 min. The supernatant was then frozen whilst the acid-insoluble residue was gelatinised with 6 M Guanidine hydrochloride/5 mM Tris-HCl for a further 18 hours. The fraction of acid-soluble proteins (predominantly collagen) was then applied to a 10 kDa ultrafilter (Vivaspin, UK) and centrifuged, which was repeated with the centrifuged supernatant from the acid-insoluble residue extraction. After the solubilised proteins had passed through the filter, two volumes of 50 mM ammonium bicarbonate (ABC) were also passed through. A further 200 μL ABC was added to the filter, mixed and recovered, which was then incubated with 10 μL 100 mM dithiothreitol (in 50 mM ABC) for 10 min at 60°C. 40 μL of iodoacetamide was added to each sample and then stored in the dark at room temperature for 45 min followed by the addition of a further 10 μL 100 mM dithiothreitol. The sample was then digested overnight with 2 μg sequencing grade trypsin (Promega, UK) at 37°C and subsequently cleaned using C18 ziptips following manufacturer’s procotol (Varian OMIX, UK), dried down and resuspended with 10 μL 0.1% trifluoroacetic acid. 1 μL of each sample was then spotted onto a Bruker 384 well Matrix Assisted Laser Desorption Ionization (MALDI) target plate and co-crystalised with 1 μL alpha-cyano hydroxycinnamic acid prior to MALDI analysis. MALDI spectra were acquired on a Bruker Ultraflex II with a Time of Flight (ToF) mass analyser, over an *m/z* range of 700–3700 using 1000 laser acquisitions.

Samples were also analysed by LC—MS/MS using an UltiMate^®^ 3000 Rapid Separation LC (RSLC, Dionex Corporation, Sunnyvale, CA) coupled to an Orbitrap Elite (Thermo Fisher Scientific, Waltham, MA) mass spectrometer (120 k resolution, Full Scan, Positive mode, normal mass range 350–1500). Peptides in the sample were separated on a 75 mm × 250 μm i.d. 1.7 μM Ethylene Bridged Hybrid (BEH) C18 analytical column (Waters, UK) using a gradient from 92% A (0.1% formic acid in water) and 8% B (0.1% formic acid in acetonitrile) to 33% B in 44 min at a flow rate of 300 nL min−1. Peptides were then automatically selected for fragmentation by data-dependent analysis; 6 MS/MS scans (Velos ion trap, product ion scans, rapid scan rate, Centroid data; scan event: 500 count minimum signal threshold, top 6) were acquired per cycle, dynamic exclusion was employed, and 1 repeat scan (2 MS/MS scans total) was acquired in a 30 s repeat duration with that precursor being excluded for the subsequent 30 s (activation: CID, 2+ default charge state, 2 *m/z* isolation width, 35 eV normalised collision energy, 0.25 Activation Q, 10.0 ms Activation time). Peptide spectra obtained via LC—MS/MS were searched against the SwissProt database using the Mascot search engine (v. 2.2.0.6; Matrix Science, London, UK). Error tolerant searches included the fixed carbamidomethyl modification of cysteine (+57.02 Da) and the variable modifications for oxidation of lysine and proline residues (all +15.99 Da) to account for PTMs (the oxidation of lysine and proline being equivalent to hydroxylation commonly observed in collagen, the dominant protein in bone), whereas decoy searches were run with the additional variable modifications allowing for the oxidation of methionine and deamidation of asparagine and glutamine (+0.98 Da) to allow for diagenetic alterations. Enzyme specificity was limited to trypsin (trypsin/P) with one (error tolerant) or two (decoy) missed cleavages allowed, mass tolerances were set at 5 ppm for the precursor ions and 0.5 Da for the fragment ions; all spectra were considered as having either 2+ or 3+ precursors. Highest matching peptide scores for homologous sequences were then manually inspected for quality, and the most appropriate added to a custom sequence database for subsequent further Error Tolerant and decoy Mascot searches. Initial sequences were obtained via Mascot searches against the other 44 mammalian sequences obtained from the Ensembl databases and the UCSC genome browser as well as the COL1A2 sequence (81% complete) for two-toed sloth (*Choloepus*) and supported by the use of ‘mammal’ sequences whereby variable amino acid loci, across all sequences, were replaced with an X (a similar method to the Error Tolerant search mentioned above but that allow for more variable post-translational modifications).

The Mascot results from the MS/MS queries for the specimens that produced collagen PMFs were filtered to only include peptide matches greater than the highest false positive score for that individual analysis (S1 File Sheets A-F); only peptide matches found in both specimens for each extinct species were used for the sequence analyses. These sequences were then ordered by position and manually aligned in BioEdit Sequence Alignment Editor v.7.1.3.0 with X representing unknown/unmatched amino acid residues (? when at an indel site in sequences from other taxa); where isobaric residues such as isoleucine and leucine were present at the same site, the most abundant was used throughout due to the inability of the presented technique to distinguish between them (S2 File). MS/MS spectra for new unique peptides are presented in the supplementary material (Figures B-P in S3 File). Phylogenetic analyses of the concatenated collagen alpha 1 and alpha 2 sequences (via an R residue; yielding a total length of 2098 amino acid residues) were then carried out using the PhyML plugin [37] for Geneious version 7.1.2 with 44 other mammalian type 1 collagen sequences (concatenated chains) obtained from the Ensembl databases and the UCSC genome browser. The JTT + I + G model was used, identified as most appropriate by PartitionFinderProtein v1.1.1 [38]. Trees were rooted to the duck-billed platypus (*Ornithorhynchus*) as a prototherian out-group. 10,000 bootstraps were carried out to estimate support with NNI branch swapping. Bayesian analyses were also carried out using the MrBayes 3.2.2 [39] with 3,000,000 MCMC generations, discarding the first 25% as burn-in, estimated invariable gamma distribution (4 categories), 4 chains (3 heated, 1 cold) with unconstrained branch lengths and also rooted to the duck-billed platypus (*Ornithorhynchus*). For analyses including only alpha 2 (I) sequences, the *Choloepus* sequence.

## Results

**Fig 1:**
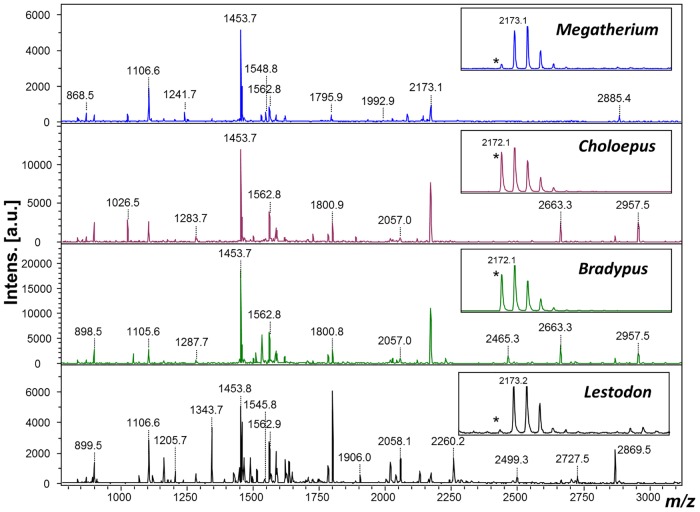
MALDI-ToF mass spectra of collagen extracted from *Lestodon* and *Megatherium* digested with trypsin. *Note the clearly observable difference in deamidation as a marker for protein ageing due to the presence of a glutamine residue in this peptide.

MALDI mass spectrometric fingerprints of the extracted *Megatherium* and *Lestodon* bone collagen (Fig 1) were evaluated primarily for qualitative purposes to confirm the extraction and subsequent enzymatic digestion of protein from the sub-fossil material, but peak differences were also studied for potential variations that could aid the subsequent in-depth proteome analysis. Close inspection of the fingerprints (Fig 1) indicate numerous potentially homologous differences (Table 1), each likely to possess one or more amino acid substitutions. The number of shared MALDI peaks was substantially greater between the two extant sloths than between any other pair of taxa (Table 1). By comparison there are more amino acid substitutions in the recovered proteomic data between the two extinct ground sloths identified from the LC-MS/MS data (Table 1; S1 File). However, it should be noted that sequence coverage can be highly variable between analyses of different species, ranging from 56% to 77% when only peptide matches above the highest scoring false positive match are used (Table 2).

**Table 1:** Number of amino acid variations detected through LC-based methods compared with number of most intense 100 peaks shared in MALDI fingerprints (numbers in brackets indicate observations confirmed in both fossil replicates for each species).

Taxa	*Choloepus (MALDI)*	*Bradypus(MALDI)*	*Lestodon (MALDI)*	*Megatherium (MALDI)*
***Choloepus* (ESI)**		72	42	43
***Bradypus* (ESI)**	0		47	43
***Lestodon* (ESI)**	11(9)	9(8)		46
***Megatherium* (ESI)**	16(9)	13(9)	15(5)	

**Table 2:** Proteomics information relating to the quality of the Mascot search results, including the False Decoy Rate (FDR), highest scoring false positive peptide (HFPS), the total protein score for sloth collagen, the number of peptide matches used for this score, the number of unique sequences and the percentage coverage.

Sample	Location	FDR	HFPS	Score	Matches	Sequences	% Cov.
*Choloepus*	Modern	1.99	38	20144	537	110	72
*Bradypus*	Modern	1.77	29	21166	669	136	77
Lestodon (474)	Arroyo del Vizcaíno	1.97	27	31482	1198	114	70
Lestodon (975a)	Arroyo del Vizcaíno	2.17	43	18467	462	81	57
Megatherium (PdlT)	Penas de las Trampas 1.1	1.22	39	12368	310	90	56
Megatherium (LC)	Las Chacras, Rio Negro	3.05	25	52195	1853	151	76

**Fig 2:**
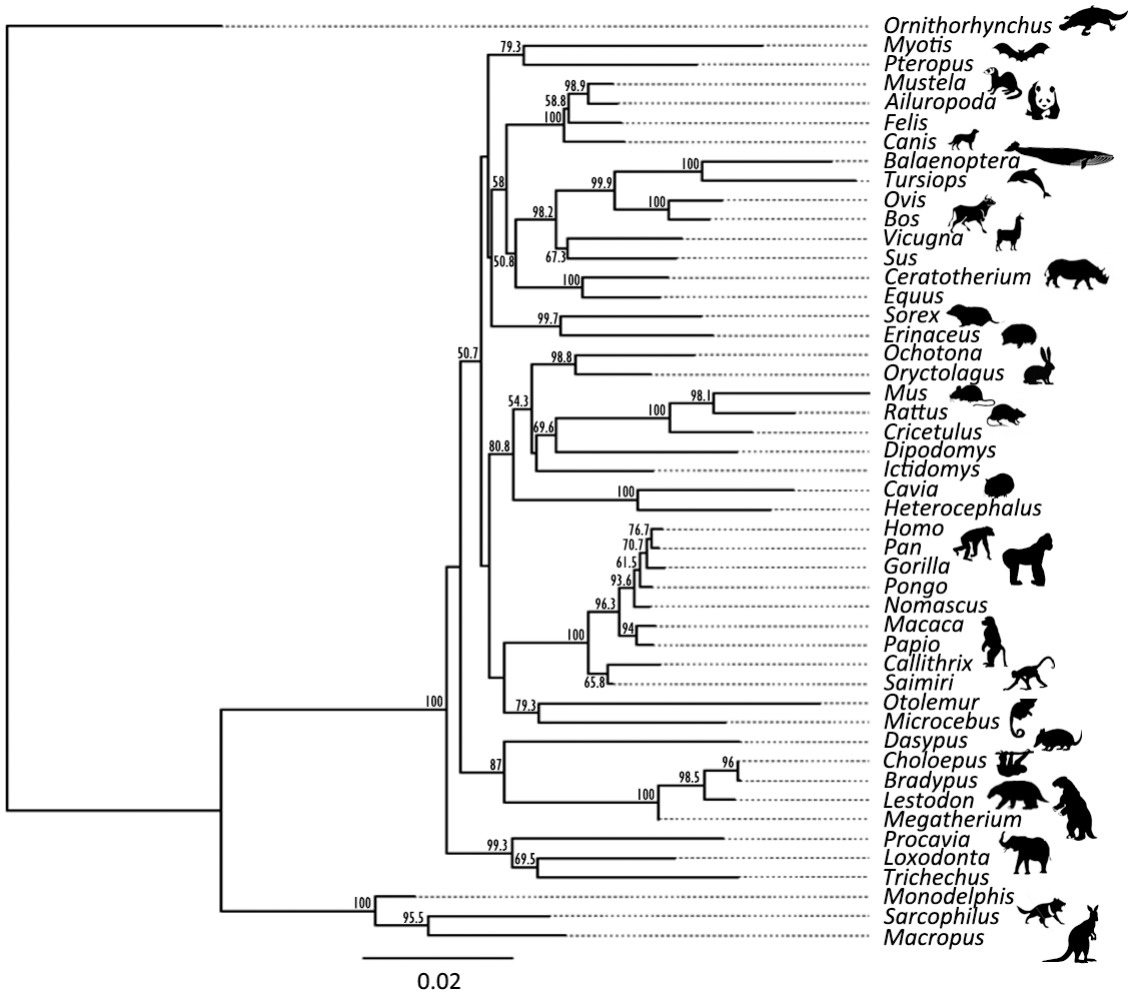
Maximum Likelihood analysis of the concatenated COL1A1 and COL1A2 sequences of eutherian mammals including the proposed consensus sequences of the two extinct ground sloths *Lestodon* and *Megatherium*.

Although there is currently no available COL1A1 sequence for any extant sloth, there is a partial COL1A2 two-toed sloth (*Choloepus hoffmanni*) sequence. When the sequence dataset is cropped to the COL1A2 sequence only and the LC-MS/MS analysis of the three-toed sloth (*Bradypus variegates*) added, the *Bradypus* is consistently placed sister to *Choloepus*, with *Lestodon* and then *Megatherium* forming a grade, and the remainder of the tree still consistent with expected phylogeny for all extant taxa with strong order-level support in most cases, but weak support at the super-order level. In terms of the stability of the xenarthran group on the whole, in the Maximum Likelihood analyses Xenarthra is consistently placed as sister to Boreoeutheria (Laurasiatheria + Euarchontoglires) with Afrotheria as the basal group (Fig 2) even when all other xenarthran sequence information from extant taxa (*Dasypus* and *Choloepus*) are not used (S1 File).

## Discussion

Both the comparison of the MALDI fingerprints and, to a lesser extent, the proteomics-based sequencing indicate that the type 1 collagen is noticeably more different between with the Folivora (sloth) suborder than initially anticipated given their estimated divergence times. By comparison to the Proboscidea, within which we only typically see one or two differences between families (Elephantidae—Mammutidae; [40]) that diverged ~20 Ma, the xenarthran mylodontids and megalonychids are known as well-established groups in South America since Deseadan times (late Oligocene, 28–24 Ma; [41, 42]; and megatherioids since the Santacruzian (early—middle Miocene, 19–16 Ma; [42]). With regards to the two extinct sloths in this study, although we observed numerous points of likely amino acid variation through analysis of the collagen-dominant MALDI fingerprints, we only confirmed the variation of fifteen of these through LC-based sequencing, which reduced to only five substitutions using results from duplicate specimens. Although one amino acid substitution could be responsible for multiple observed peak differences in MALDI, due to the presence of both missed cleavages as well as post-translational modifications, this is indicative of the bias against discovering sequence variation in more distantly related taxa. This is particularly clear in the example of the inability to confirm all of the sequences of peptides described previously as peptide biomarkers (e.g., marker F in [43]) in *Megatherium*, and subsequent identification of the homologous peptide in *Lestodon*, identical to that of the extant taxa. This discrepancy of missing peptide sequence information specific to particular regions of the molecular may impact upon phylogenetic inferences from proteomics-derived sequence data to an extent that has not yet been explored.

**Fig 3:**
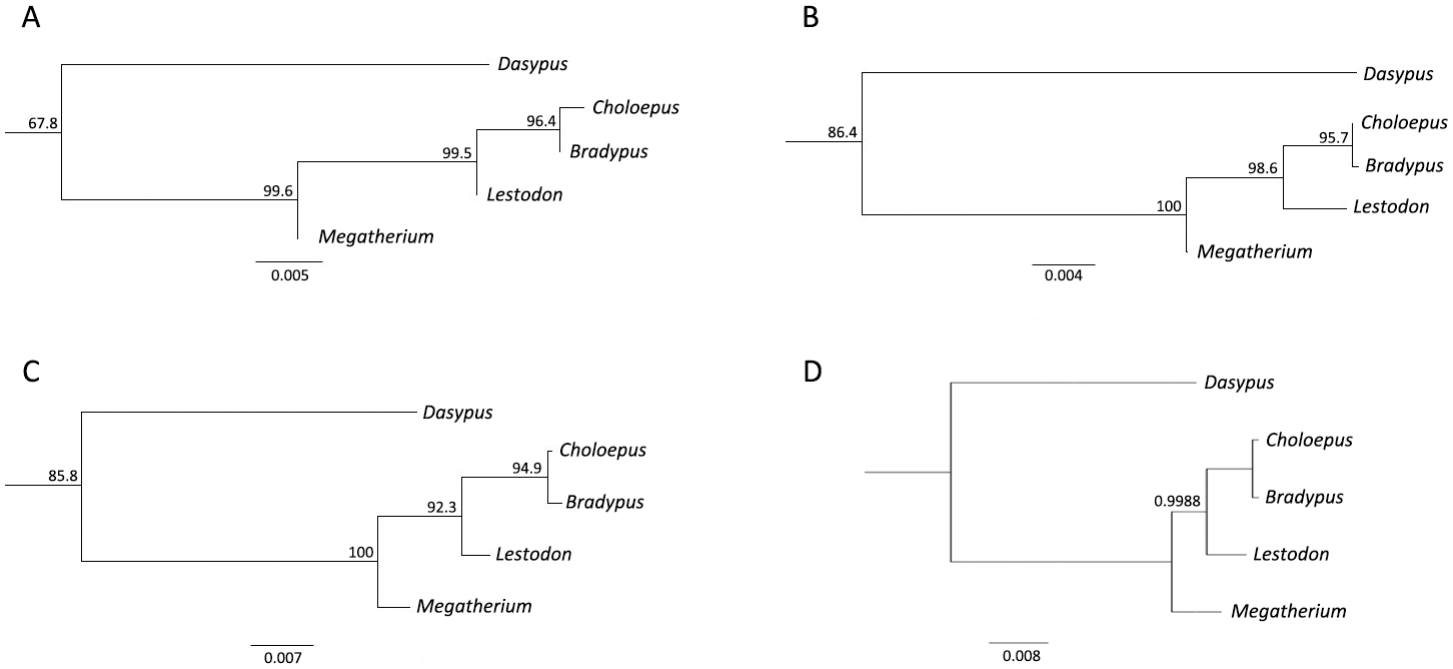
Phylogenetic analyses of COL1A2 sequences of eutherian mammals including the extinct South American native ungulates and two extinct ground sloths *Lestodon* and *Megatherium* in comparison to extant sloths *Bradypus* and *Choloepus* showing (A) Maximum Likelihood analysis of consensus peptide matches observed in the PMF, (B) Maximum Likelihood of peptide matches observed in the PMF from either specimen, (C) Maximum Likelihood of the alpha 2 (I) sequences only, using the Ensembl *Choloepus* sequence and (D) Bayes analysis of consensus peptide matches observed in the PMF.

Previous studies on the phylogenetic relationships of sloths showed a clear distinction between the two extant families (Megalonychidae and Bradypodidae) well supported by both morphological and molecular differences. In particular, Sarich [44] found considerable evolutionary distance between the albumins of the two genera. Previous aDNA studies on extinct sloths [19, 45] indicated a relationship between, on the one hand, *Mylodon darwinii* (Mylodontidae) and *Choloepus* (Megalonychidae), and, on the other, between *Nothrotheriops shastensis* (Megatheriidae) and *Bradypus* spp. (Bradypodidae). However, this assumption was questioned by more recent studies [46]. The inclusion of Mylodontidae as the sister taxon of Megalonychidae does not support the monophyly of Megatherioidea (including the megatheriids, nothrotheriids and megalonychids) as in the widely accepted phylogenetic reconstruction proposals by Gaudin [47] based on craniodental morphological traits. Although in that paper the grouping of Mylodontidae and Megalonychidae is not supported, most of the molecular phylogenetic reconstructions show an allied relation of *Mylodon* and *Choloepus* [19, 45, 46, 48], even with the inclusion of other Megatherioidea as *Nothrotheriops* [20]. Moreover, Gaudin [47] states that the hypothesis of a close relation of *Choloepus* with mylodontids cannot be statistically rejected and that the position of *Choloepus* within megalonychids could not unambiguously be resolved. More recently, an aDNA phylogeny by Clack et al. [49] placed *Choloepus* close to *Mylodon*, with *Bradypus* occupying a derived position among megalonychids and *Nothrotheriops* occupying a basal position as the sister group of all the mentioned sloths. Our study shows a relationship between the extant and extinct sloths not previously proposed by molecular or morphological phylogenies, since both extant sloths appear as sister taxa with *Lestodon* (Mylodontidae) and *Megatherium* (Megatheriidae) forming a successive grade. As noted before, a basal position of Megatheriidae (*Nothrotheriops*) is reported by Clack et al. [49] but the Mylodontidae groups with *Choloepus*, as most aDNA phylogenies. The discrepancies between the collagen, aDNA and morphological phylogenies could be due to the incompleteness of the collagen data, as alluded to earlier. One of the limitations of proteomics-based approaches to phylogenetic reconstruction that needs to be addressed is deciding on an appropriate level of confidence—in this case we have chosen to rely on a peptide score equivalent to the highest false positive match in order to accommodate for differences in the quality of each analysis; this approach is likely to unnecessarily reduce sequence coverage, but comparison of the phylogenies recovered here (e.g., Fig 3A and 3B) imply that the variation in percentage sequence coverage alone in this study is not an issue. Also, the fact that the MALDI fingerprints also show greater similarity between the two extant taxa implies that perhaps it is more likely related to the more limited source information of the collagen-based approach in relation to the other methods. In regard to the phylogenetic relationships of the fossil sloths, belonging to Mylodontidae and Megatheriidae, it should be noted that the absence of northotheriids in our analysis leaves uncertain the possible placement of this taxon, which has been closely associated to Megatheriidae in most morphological studies [47, 50].

In conclusion, given that the resulting topologies of these extinct xenarthran taxa remain consistent, with or without the inclusion of any other xenarthran sequence, the analysis of collagen using proteomics techniques clearly has potential to resolve many currently ambiguous relationships in palaeontology that are currently dominated by morphological analyses. Although collagen sequence phylogenies will not yield as great a phylogenetic resolution as DNA-based analyses, they have the potential to produce results that are to some extent congruent and that can be applied to vertebrates that went extinct much earlier (e.g., millions of years in ideal conditions) than can be currently achieved with the DNA-based methods. However, the minor disagreement with aDNA and morphological methods at the higher taxonomic level could imply that a wider range of proteins [51] should be investigated to further improve the potential of proteomics applications to palaeontology and the evolution of vertebrate life.

## Supporting Information

S1 File Excel files of peptide matches for each sample: modern *Bradypus* (Sheet A), modern *Choloepus* (Sheet B), extinct *Lestodon* from Arroyo del Vizcaíno #474 (Sheet C), extinct *Lestodon* from Arroyo del Vizcaíno #474 (Sheet D), extinct *Megatherium* Penas de las Trampas (Sheet E), and extinct *Megatherium* from Las Chacras (Sheet F). Raw data can be accessed on http://www.peptideatlas.org/PASS/PASS00753.
(XLSX)

S2 File Concatenated COL1A1 and COL1A2 sequences, and additional COL1A2 sloth sequences. (DOCX)

S3 File Supplementary figures (Figures A-P) including additional Maximum Likelihood phylogenetic tree (Figure A) and tandem mass spectra for new unique peptide sequences (Figures B-P).
(ZIP)
