# Correction: Collagen Sequence Analysis of the Extinct Giant Ground Sloths *Lestodon* and *Megatherium*

**DOI:** 10.1371/journal.pone.0144793

**Published:** 2015-12-07

**Authors:** Michael Buckley, Richard A. Fariña, Craig Lawless, P. Sebastián Tambusso, Luciano Varela, Alfredo A. Carlini, Jaime E. Powell, Jorge G. Martinez

The following information is missing from the Funding section: Grant name: Using Fossil Proteomics for Resolving Phylogenetics of Extinct Mammalian Orders in Ancient Biodiversity Hotspots Principal. Investigator: Dr M Buckley, The University of Manchester, Life Sciences. Grant held at: The University of Manchester, Life Sciences. NERC Reference: NE/K000799/1
