# Supplementary material for: Collagen Sequence Analysis of the Extinct Giant Ground Sloths Lestodon and Megatherium
Source: PLoS One. 2015 Nov 5;10(11):e0139611. doi: 10.1371/journal.pone.0139611 (PMC4634953; doi:10.1371/journal.pone.0139611)
Supplement: S2 File — (DOCX) [file pone.0139611.s002.docx]

**Sequences**

**The sloth sequences were acquired from this study, and compared with concatenated mammalian collagen sequences taken from Buckley 2015, with the exception of the *Choloepus* COL1A2 sequence which was taken from the Ensembl Genome Browser,** ENSCHOT00000000336.

>MEGATHERIUM_CONSENSUS

XXXXXXXXXXXX?XXXXXXXXXXXXXXXXXXXXXXXXXXXXXXXXXXXXXXXXXXXXXXX

XXXXXXXXXXXXXXXXXXXXXXXXXXXXXXXXGLPGTAGLPGMKXXXGFSGLDGAKGDAG

PAGPKGEPGSPGENGAPGQMGPRXXXXXXGRPGASGPAGARGNDGATGAAGPPGPTGPAG

PPGFPGAVGAKGEAGPQGARGSEGPQGVRGEPGPPGPAGAAGPAGNPGADGQPGAKGANG

APGIAGAPGFPGARGPSGPQGPSGPPGPKGNSGEPGAPGSKXXXXXXGEPGPTGIQGPPG

PAGEEGKRXXXGEPGPTGLPGPPGERGGPGSRGFPGADGVAGPKGPAGERGSPGPAGPKX

XXXXXXXXXXXXXXXXXGLTGSPGSPGPDGKTGPPGPAGQDGRPGPPGPPGARGQAGVMG

FPGPKGAAGEPGKAGERGVPGPPGAVGPAGKDGEAGAQGPPGPAGPAGERXXXXXXXXXX

XXXXXXXXXXXXXXXXXXXXXXXXXXXXXXXXXXXXXXXXXXXXGVQGPPGPAGPRGSNG

APGNDGAKGDAGAPGAPGSQGAPGLQGMPGERGAAGLPGPKGDRGDAGPKGADGAPGKXX

XXXXXXXXXXXXXXXXXXXXXXXXXXXXXXXXXXXXXXXXXGEPGPPGPAGFAGPPGADG

QPGAKXXXXXXXXXGDAGPAGPAGPTGPPGPIGNVGAPGPKGARGSAGPPGATGFPGAAG

RVGPPGPSGNAGPPGPPGPVGKXXXXXXXGETGPAGRPGEVGPPGPPGPTGEKGSPGADG

PAGAPGTPGPQGIAGQRGVVGLPGQRXXXGFPGLPGPSGEPGKQGPSGSSGERGPPGPVG

PPGLAGPPGESGREGAPGAEGSPGRDGSPGPKGDRGESGPAGPPGAPGAPGAPGPVGPAG

KxxxxxxxxxxxxxxxxxxxxxxGPAGPQGPRGDKGETGEAGDRXXXXXXGFSGLQGPaG

pPGSPGEQGPSGASGPAGPRGPPGSAGSPGKDGLNGLPGPIGPPGPRXXXXXXXXXXXXX

XXXXXXXXXXXXXXXXXXXXXXXXXXXXXXXXXXXXXXXXX?XXGVGLGPGPMGLMGPRX

XXXXXXXXXXXXXXXXXXXXXXXXXXXXXXXXXXXXXXXXXXXXXXXXXXXXXXXXXXXX

XXXXXGFPGTPGLPGFKXXXxxxxxxxxxxxxxxxxxxXXXXXXXXXXXXXXXXXXXXXX

XXXXVGAPGPAGARGSDGSVGPVGPAGPIGSAGPPGFPGAPGPKGELGPVGNTGPSGPAG

PRXXXXXXXXXXXXXXXXXXXXXXXXXXXGAAGLPGVAGAPGLPGPRGIPGPAGASGATG

ARGLVGEPGPAGSKxxxxxxGEPGSAGPQGPPGSSGEEGKRGPNGESGSTGPTGPPGLRX

XXXXXGLPGADGRAGVIGPAGARxxxxxxxxxXXXXXXXXxxxxxxxxxxGLPGSPGNVG

PAGKEGPAGLPGIDGRPGPIGPAGARGEAGNIGFPGPKXXXXXXXXXXXXGHAGLAGNRx

xxxxxxxxxxxxxxxxxxxxxxxXXXXXXXXXXXXXXXXXXXXXXXXXXXXXXGLPGEFG

LPGPAGPRGERGPPGESGAVGPSGAIGSRXXXXXXXXXXXXXXXXXXXXXXXXXXXXXXX

XXXXXGAAGIPGGKGEKGETGLRXXXXXXXXXXXXXXXXXXXXXXXXXXXXXXXXXXXXX

XXXXXXXXXXXXXXGEVGPAGPNGFAGPAGAAGQPGAKGERXXXGPKGENGIVGPTGPVG

SAGPAGPNGPAGPAGSRGDGGPPGMTGFPGAAGRTGPPGPSGITGPPGPPGAAGKXXXXX

XXXXXXXXXXAGETGAGGPPGFTGEKGPSGEPGTAGPPGTAGPQGLLGAPGILGLPGSRG

ERGLPGVAGAVGEPGPLGIQGPPGARGPSGAVGGPGVNGAPGEAGRDGNPGSDGPPGRXX

XXXXXXXXXXXXXXXXXXXXXXXXXXXXXXXXXXHGNRGEPGPVGSVGPVGAIGPRGPSG

PQGIRxxxxxxxxxxxxxxxxxxxxxxxxxxxxxxxxxxxxxxxxxxxxxxxxGPAGPSG

PAGKdgrTGHPGAVGPAGVRXXXXXXXXXXXXXXXXXXXXXXXXXXXXXXXXXXXXXXX

>LESTODON_CONSENSUS

XXXXXXXXXXXX?XXXXXXXXXXXXXXXXXXXXXXXXXXXXXXXXXXXXXXXXXXXXXXX

XXXXXXXXXXXXXXXXXXXXXXXXXXXXXXXXXXXXXXXXXXXXXXXGFSGLDGAKGDAG

PAGPKGEPGSPGENGAPGQMGPRXXXXXXGRPGASGPAGARGNDGATGAAGPPGPTGPAG

PPGFPGAVGAKXXXXXXXXXGSEGPQGVRGEPGPPGPAGAAGPAGNPGADGQPGAKGANG

APGIAGAPGFPGARGPSGPQGPSGSPGPKXXXXXXXXXXXXXXXXXXGEPGPTGIQGPPG

PAGEEGKRXXXGEPGPTGLPGPPGERGGPGSRGFPGADGVAGPKXXXXXXXXXXXXXXXX

XXXXXXXPGEAGLPGAKGLTGSPGSPGPDGKXXXXXXXXXXXXXXXXXXXXXXGQAGVMG

FPGPKXXXXXXXXXXXXGVPGPPGAVGPAGKDGEAGAQGPPGPAGPAGERXXXXXXXXXX

XXXXXXXXXXXXXXXXXXXXXXXXXXXXXXXXXXXXXXXXXXXXGVQGPPGPAGPRXXXX

XXXXXXXXGDAGAPGAPGSQGAPGLQGMPGERXXXXXXXXXXXXXXXXXXXXXXXXXXXX

XXGLTGPIGPPGPAGAPGDKGEsGPSGPAGPTGARXXXXXXGEPGPPGPAGFAGPPGADG

QPGAKXXXXXXXXXGDAGPPGPAGPTGPPGPIGNVGAPGPKXXXGSAGPPGATGFPGAAG

RXXXXXXXXXXXXXXXXXXXXXXXXXXXXXXXXXXXXXXXXXXXXXXXXXXXXXXXXXXX

XXXXXXXXXXXXXXXXXGVVGLPGQRXXXGFPGLPGPSGEPGKQGPSGSSGERGPPGPVG

PPGLAGPPGESGRXXXXXXXXXXXXXXXXXXXGDRGESGPAGPPGAPGAPGAPGPVGPAG

KSGDRGETGPAGPAGPAGPAGARXXXXXXXXXGDKGETGEaGDRXXXXXXGFSGLQGPaG

pPGSPGEQGPSGASGPAGPRGPPGSAGSPGKDGLNGLPGPIGPPGPRXXXXXXXXXXXXX

XXXXXXXXXXXXXXXXXXXXXXXXXXXXXXXXXXXXXXXXX?XXX?X?XXXXXXXXXXXX

XXXXXXXXXXXXXXXXXXXXXXXXXXXXXXXXXXXXXXXXXXXXXXXXXXXXXXXXXXXX

XXXXXGFPGTPGLPGFKXXXGHNGLDGLKGQPGAPGVKXXXXXXXXXXXXXXXXXXXXXX

XXXXXXXXXXXXXXGSDGSVGPVGPAGPIGSAGPPGFPGAPGPKGELGPVGNTGPSGPAG

PRGEQGLPGVSGPVGPPGNPGANGLTGAKGAAGLPGVAGAPGLPGPRGIPGPVGASGATG

ARGLVGEPGPAGSKGESGGKGEPGSAGPQGPPGSSGEEGKRGPSGESGSTGPTGPPGLRX

XXXXXXXXXXXXXXXXXXXXXXXXXXXXXXXXXXXXXXXXPGEPGLMGARGLPGSPGNVG

PAGKEGPAGLPGIDGRPGPIGPAGARGEAGNIGFPGPKXXXXXXXXXXXXXXXXXXXXXG

APGPDGNNGAQGPPGLQGVQGGKXXXXXXXXXXXXXXXXXXXXXXXXXXXXXXGLPGEFG

LPGPAGPRXXXGAPGESGAVGPSGAIGSRXXXXXXXXXXXXXXXXXXXXXXXXXXXXXXX

XXXXXXXXXXXXXXXXXXXXXXXXXXXXXXXXXXXGAPGAVGAPGPAGATGDRGEAGAAG

PAGPAGPRXXXXXXGEVGPAGPNGFAGPAGAAGQPGAKGERXXXGPKGENGIVGPTGPVG

SAGPAGPNGPAGPAGSRGDGGPPGITGFPGAAGRTGPPGPSGITGPPGPPGAAGKXXXXX

XXXXXXXXXXAGETGAGGPPGFTGEKGPSGEPGTAGPPGTAGPQGLLGAPGILGLPGSRX

XXGLPGVAGAVGEPGPLGIQGPPGARGPSGAVGGPGVNGAPGEAGRDGNPGSDGPPGRXX

XXXXXXXXGYAGNPGPAGAAGAPGPHGAVGPAGKHGNRGEPGPVGSAGPVGAIGPRGPSG

PQGIRXXXXXXXXXXXXXXXXXXXXXXXXXXXXXXXXXXXXXXXXXXXXXXXXGPAGPSG

PPGKDGRTGHPGAVGPAGIRXXXXXXXXXXXXXXXXXXXXXXXXXXXXXXXXXXXXXXX

>MEGATHERIUM_SINGLE_BEST

XXXXXXXXXXXX?XXXXXXXXXXXXXXXXXXXXXXXXXXXXXXXXXXXXXXXXXXXXXXX

XXXXXXXXXXXXXXXXXXXXXXXXXXXXXXXXGLPGTAGLPGMKXXXGFSGLDGAKGDAG

PAGPKGEPGSPGENGAPGQMGPRXXXXXXGRPGASGPAGARGNDGATGAAGPPGPTGPAG

PPGFPGAVGAKGEAGPQGARGSEGPQGVRGEPGPPGPAGAAGPAGNPGADGQPGAKGANG

APGIAGAPGFPGARGPSGPQGPSGPPGPKGNSGEPGAPGSKXXXXXXGEPGPTGIQGPPG

PAGEEGKRXXXGEPGPTGLPGPPGERGGPGSRGFPGADGVAGPKGPAGERGSPGPAGPKX

XXXXXXXXXXXXXXXXXGLTGSPGSPGPDGKTGPPGPAGQDGRPGPPGPPGARGQAGVMG

FPGPKGAAGEPGKAGERGVPGPPGAVGPAGKDGEAGAQGPPGPAGPAGERXXXXXXXXXX

XXXXXXXXXXXXXXXXXXXXXXXXXXXXXXXXXXXXXXXXXXXXGVQGPPGPAGPRGSNG

APGNDGAKGDAGAPGAPGSQGAPGLQGMPGERGAAGLPGPKGDRGDAGPKGADGAPGKXX

XXXXXXXXXXXXXXXXXXXXXXXXXXXXXXXXXXXXXXXXXGEPGPPGPAGFAGPPGADG

QPGAKXXXXXXXXXGDAGPAGPAGPTGPPGPIGNVGAPGPKGARGSAGPPGATGFPGAAG

RVGPPGPSGNAGPPGPPGPVGKXXXXXXXGETGPAGRPGEVGPPGPPGPTGEKGSPGADG

PAGAPGTPGPQGIAGQRGVVGLPGQRXXXGFPGLPGPSGEPGKQGPSGSSGERGPPGPVG

PPGLAGPPGESGREGAPGAEGSPGRDGSPGPKGDRGESGPAGPPGAPGAPGAPGPVGPAG

KxxxxxxxxxxxxxxxxxxxxxxGPAGPQGPRGDKGETGEAGDRXXXXXXGFSGLQGPaG

pPGSPGEQGPSGASGPAGPRGPPGSAGSPGKDGLNGLPGPIGPPGPRXXXXXXXXXXXXX

XXXXXXXXXXXXXXXXXXXXXXXXXXXXXXXXXXXXXXXXX?XXGVGLGPGPMGLMGPRX

XXXXXXXXXXXXXXXXXXXXXXXXXXXXXXXXXXXXXXXXXXXXXXXXXXXXXXXXXXXX

XXXXXGFPGTPGLPGFKXXXxxxxxxxxxxxxxxxxxxXXXXXXXXXXXXXXXXXXXXXX

XXXXVGAPGPAGARGSDGSVGPVGPAGPIGSAGPPGFPGAPGPKGELGPVGNTGPSGPAG

PRXXXXXXXXXXXXXXXXXXXXXXXXXXXGAAGLPGVAGAPGLPGPRGIPGPAGASGATG

ARGLVGEPGPAGSKxxxxxxGEPGSAGPQGPPGSSGEEGKRGPNGESGSTGPTGPPGLRX

XXXXXGLPGADGRAGVIGPAGARxxxxxxxxxXXXXXXXXxxxxxxxxxxGLPGSPGNVG

PAGKEGPAGLPGIDGRPGPIGPAGARGEAGNIGFPGPKXXXXXXXXXXXXGHAGLAGNRx

xxxxxxxxxxxxxxxxxxxxxxxXXXXXXXXXXXXXXXXXXXXXXXXXXXXXXGLPGEFG

LPGPAGPRGERGPPGESGAVGPSGAIGSRXXXXXXXXXXXXXXXXXXXXXXXXXXXXXXX

XXXXXGAAGIPGGKGEKGETGLRXXXXXXXXXXXXXXXXXXXXXXXXXXXXXXXXXXXXX

XXXXXXXXXXXXXXGEVGPAGPNGFAGPAGAAGQPGAKGERXXXGPKGENGIVGPTGPVG

SAGPAGPNGPAGPAGSRGDGGPPGMTGFPGAAGRTGPPGPSGITGPPGPPGAAGKXXXXX

XXXXXXXXXXAGETGAGGPPGFTGEKGPSGEPGTAGPPGTAGPQGLLGAPGILGLPGSRG

ERGLPGVAGAVGEPGPLGIQGPPGARGPSGAVGGPGVNGAPGEAGRDGNPGSDGPPGRXX

XXXXXXXXXXXXXXXXXXXXXXXXXXXXXXXXXXHGNRGEPGPVGSVGPVGAIGPRGPSG

PQGIRxxxxxxxxxxxxxxxxxxxxxxxxxxxxxxxxxxxxxxxxxxxxxxxxGPAGPSG

PAGKdgrTGHPGAVGPAGVRXXXXXXXXXXXXXXXXXXXXXXXXXXXXXXXXXXXXXXX

>LESTODON_SINGLE_BEST

XXXXXXXXXXXX?XXXXXXXXXXXXXXXXXXXXXXXXXXXXXXXXXXXXXXXXXXXXXXX

XXXXXXXXXXXXXXXXXXXXXXXXXXXXXXXXXXXXXXXXXXXXXXXGFSGLDGAKGDAG

PAGPKGEPGSPGENGAPGQMGPRXXXXXXGRPGASGPAGARGNDGATGAAGPPGPTGPAG

PPGFPGAVGAKXXXXXXXXXGSEGPQGVRGEPGPPGPAGAAGPAGNPGADGQPGAKGANG

APGIAGAPGFPGARGPSGPQGPSGSPGPKXXXXXXXXXXXXXXXXXXGEPGPTGIQGPPG

PAGEEGKRXXXGEPGPTGLPGPPGERGGPGSRGFPGADGVAGPKXXXXXXXXXXXXXXXX

XXXXXXXPGEAGLPGAKGLTGSPGSPGPDGKXXXXXXXXXXXXXXXXXXXXXXGQAGVMG

FPGPKXXXXXXXXXXXXGVPGPPGAVGPAGKDGEAGAQGPPGPAGPAGERXXXXXXXXXX

XXXXXXXXXXXXXXXXXXXXXXXXXXXXXXXXXXXXXXXXXXXXGVQGPPGPAGPRXXXX

XXXXXXXXGDAGAPGAPGSQGAPGLQGMPGERXXXXXXXXXXXXXXXXXXXXXXXXXXXX

XXGLTGPIGPPGPAGAPGDKGEsGPSGPAGPTGARXXXXXXGEPGPPGPAGFAGPPGADG

QPGAKXXXXXXXXXGDAGPPGPAGPTGPPGPIGNVGAPGPKXXXGSAGPPGATGFPGAAG

RXXXXXXXXXXXXXXXXXXXXXXXXXXXXXXXXXXXXXXXXXXXXXXXXXXXXXXXXXXX

XXXXXXXXXXXXXXXXXGVVGLPGQRXXXGFPGLPGPSGEPGKQGPSGSSGERGPPGPVG

PPGLAGPPGESGRXXXXXXXXXXXXXXXXXXXGDRGESGPAGPPGAPGAPGAPGPVGPAG

KSGDRGETGPAGPAGPAGPAGARXXXXXXXXXGDKGETGEaGDRXXXXXXGFSGLQGPaG

pPGSPGEQGPSGASGPAGPRGPPGSAGSPGKDGLNGLPGPIGPPGPRXXXXXXXXXXXXX

XXXXXXXXXXXXXXXXXXXXXXXXXXXXXXXXXXXXXXXXX?XXX?X?XXXXXXXXXXXX

XXXXXXXXXXXXXXXXXXXXXXXXXXXXXXXXXXXXXXXXXXXXXXXXXXXXXXXXXXXX

XXXXXGFPGTPGLPGFKXXXGHNGLDGLKGQPGAPGVKXXXXXXXXXXXXXXXXXXXXXX

XXXXXXXXXXXXXXGSDGSVGPVGPAGPIGSAGPPGFPGAPGPKGELGPVGNTGPSGPAG

PRGEQGLPGVSGPVGPPGNPGANGLTGAKGAAGLPGVAGAPGLPGPRGIPGPVGASGATG

ARGLVGEPGPAGSKGESGGKGEPGSAGPQGPPGSSGEEGKRGPSGESGSTGPTGPPGLRX

XXXXXXXXXXXXXXXXXXXXXXXXXXXXXXXXXXXXXXXXPGEPGLMGARGLPGSPGNVG

PAGKEGPAGLPGIDGRPGPIGPAGARGEAGNIGFPGPKXXXXXXXXXXXXXXXXXXXXXG

APGPDGNNGAQGPPGLQGVQGGKXXXXXXXXXXXXXXXXXXXXXXXXXXXXXXGLPGEFG

LPGPAGPRXXXGAPGESGAVGPSGAIGSRXXXXXXXXXXXXXXXXXXXXXXXXXXXXXXX

XXXXXXXXXXXXXXXXXXXXXXXXXXXXXXXXXXXGAPGAVGAPGPAGATGDRGEAGAAG

PAGPAGPRXXXXXXGEVGPAGPNGFAGPAGAAGQPGAKGERXXXGPKGENGIVGPTGPVG

SAGPAGPNGPAGPAGSRGDGGPPGITGFPGAAGRTGPPGPSGITGPPGPPGAAGKXXXXX

XXXXXXXXXXAGETGAGGPPGFTGEKGPSGEPGTAGPPGTAGPQGLLGAPGILGLPGSRX

XXGLPGVAGAVGEPGPLGIQGPPGARGPSGAVGGPGVNGAPGEAGRDGNPGSDGPPGRXX

XXXXXXXXGYAGNPGPAGAAGAPGPHGAVGPAGKHGNRGEPGPVGSAGPVGAIGPRGPSG

PQGIRXXXXXXXXXXXXXXXXXXXXXXXXXXXXXXXXXXXXXXXXXXXXXXXXGPAGPSG

PPGKDGRTGHPGAVGPAGIRXXXXXXXXXXXXXXXXXXXXXXXXXXXXXXXXXXXXXXX

>MEGATHERIUM_SINGLE_PMF

XXXXXXXXXXXX?XXXXXXXXXXXXXXXXXXXXXXXXXXXXXXXXXXXXXXXXXXXXXXX

XXXXXXXXXXXXXXXXXXXXXXXXXXXXXXXXxxxxxxxxxxxxxxxxxxxxxxxxGDAG

PAGPKGEPGSPGENGAPGQMGPRXXXXXXGRPGASGPAGARGNDGATGAAGPPGPTGPAG

PPGFPGAVGAKGEAGPQGARGSEGPQGVRGEPGPPGPAGAAGPAGNPGADGQPGAKGANG

APGIAGAPGFPGARxxxxxxxxxxxxxxxxxxxxxxxxxxxxxxxxxxxxxxxxxxxxxx

xxxxxxxxGARGEPGPTGLPGPPGERGGPGSRGFPGADGVAGPKGPAGERXXXXXXXXXx

xxxxxxxxxxxxxxxxxxxxxxxxxxxxxxxTGPPGPAGQDGRPGPPGPPGARGQAGVMG

FPGPKxxxxxxxxxxxxxxxxxxxxxxxxxxxxxxxxxxxxxxxxxxxxxxxxxxxxxxx

xxxxxxxxxxxxxxxxxxxxxxxxxxxxxxxxxxxxxxxxxxxxGVQGPPGPAGPRXXXX

XXXXXXXXXXXXXXXXXXXXXXXXXXXXXXXXXXXXXXXXXXXXXXXXXXXXXXXXXXXX

XXGLTGPIGPPGPAGSPGDKGESGPSGPAGPTGARGAPGDRGEPGPPGPAGFAGPPGADG

QPGAKXXXXXXXXXGDAGPAGPAGPTGPPGPIGNVGAPGPKGARGSAGPPGATGFPGAAG

RVGPPGPSGNAGPPGPPGPVGKXXXXXXXXXXXXXXXXXXXXXXXXXXXXXXXGSPGADG

PAGAPGTPGPQGIAGQRGVVGLPGQRGERGFPGLPGPSGEPGKQGPSGSSGERXXXXXXX

XXXXXXXXXXXXXXXXXXXXXXXXXXXXXXXXXXXXXXXXXXXXXXXXXXXXXXXXXXXX

XNGDRGETGPAGPAGPAGPAGARGPAGPQGPRXXXXXXXXXXXXXXXGHRGFSGLQGPAG

PPGSPGEQGPSGASGPAGPRXXXXXXXXXXXXXXXXXXXXXXXXXXXXXXXXXXXXXXXX

XXXXXXXXXXXXXXXXXXXXXXXXXXXXXXXXXXXXXXXXX?XXX?X?XXXXXXXXXXXX

XXXXXXXXXXXXXXXXXXXXXXXXXXXXXXXXXXXXXXXXXXXXXXXXXXXXXXXXxxxx

xxxxxGFPGTPGLPGFKxxxXXXXXXXXXXXXXXXXXXXXXXXXXXXXXXXXXXXXXXXX

XXxxVGAPGPAGARXXXXXXXXXXXXXXXXXXXXXXXXXXXXXXXXXXXXXXXXXXXXXX

XXXXXXXXXXXXXXXXXXXXXXXXXXXXXGAAGLPGVAGAPGLPGPRGIPGPAGASGATG

ARXXXXXXXXXXXXXXXXXXXXXXXXXXXXXXXXXXXXXXRGPNGESGSTGPTGPPGLRX

XXxxxGLPGADGRAGVIGPAGARGASGPAGVRXXXXXXXXXXXXXXXXXXGLPGSPGNVG

PAGKEGPAGLPGIDGRPGPIGPAGARGEAGNIGFPGPKXXXXXXXXXXXXGHAGLAGNRX

XXXXXXXXXXXXXXXXXXXXXXXXXXXXXXXXXXXXXXXXXXXXXXXXXXXXXGLPGEFG

LPGPAGPRGERGPPGESGAVGPSGAIGSRXXXXXXXXXXXXXXXXXXXXXXXXXXXXXXX

XXXXXXXXXXXXXXXXXXXXXXXXXXXXXXXXXXXXXXXXXXXXXXXXXXXXXxxxxxxx

xxxxxxxxxxxxxxGEVGPAGPNGFAGPAGAAGQPGAKGERXXXXXXGENGIVGPTGPVG

SAGPAGPNGPAGPAGSRXXXXXXXXXXXXXXXXXTGPPGPSGITGPPGPPGAAGKxxxxx

xxxxxxxxxxXXXXXXXXXXXXXXXXXXXXXXXXXXXXXXXXXXXXXXXXXXXXXXXXXG

ERGLPGVAGAVGEPGPLGIQGPPGARXXXXXXXXXXXXXXXXXXXXXXXXXXXXXXXXXX

xxxxxxxxXXXXXXXXXXXXXXXXXXXXXXXXXXHGNRGEPGPVGSVGPVGAIGPRGPSG

PQGIRXXXXXXXXXXXXGLPGLKGHNGLQGLPGLAGQHGDQGAPGPVGPAGPRXXXXXXX

XXXXXXXXXXXXXXXXXXXXXXXXXXXXXXXXXXXXXXXXXXXXXXXXXXXXXXXXXXX

>LESTODON_SINGLE_PMF

XXXXXXXXXXXX?XXXXXXXXXXXXXXXXXXXXXXXXXXXXXXXXXXXXXXXXXXXXXXX

XXXXXXXXXXXXXXXXXXXXXXXGPPGPQGARGLPGTAGLPGMKXXXGFSGLDGAKXXXX

XXXXXXXXXXXXXXXXXXXXXXXXXXXXXGRPGASGPAGARGNDGATGAAGPPGPTGPAG

PPGFPGAVGAKXXXXXXXXXGSEGPQGVRGEPGPPGPAGAAGPAGNPGADGQPGAKGANG

APGIAGAPGFPGARXXXXXXXXXXXXXXXXXXXXXXXXXXXXXXXXXGEPGPTGIQGPPG

PAGEEGKRXXXGEPGPTGLPGPPGERXXXXXXGFPGADGVAGPKXXXXXXXXXXXXXXXx

xxxxxxxPGEAGLPGAKXXXXXXXXXXXXXXTGPPGPAGQDGRPGPPGPPGARGQAGVMG

FPGPKXXXXXXXXXXXXXXXXXXXXXXXXXXDGEAGAQGPPGPAGPAGERXXXXXXXXXX

XXXXXXXXXXXXXXXXXXXXXXXXXXXXXXXXXXXXXXXXXXXXGVQGPPGPAGPRXXXX

XXXXXXXXXXXXXXXXXXXXXXXXXXXXXXXXxxxxxxxxxxxxXXXXXXxxxxxxxxxx

xxGLTGPIGPPGPAGAPGDKGESGPSGPAGPTGARGAPGDRGEPGPPGPAGFAGPPGADG

QPGAKxxxxxxxxxGDAGPPGPAGPTGPPGPIGNVGAPGPKXXXGSAGPPGATGFPGAAG

RVGPPGPSGNAGPPGPPGPVGKXXXXXXXXXXXXXXXXXXXXXXXXXXXXXXXGSPGADG

PAGAPGTPGPQGIAGQRGVVGLPGQRXXXGFPGLPGPSGEPGKQGPSGSSGERGPPGPVG

PPGLAGPPGESGRxxxxxxxxxxxxxxxxxxxGDRGESGPAGPPGAPGAPGAPGPVGPAG

KSGDRGETGPAGPAGPAGPAGARGPAGPQGPRXXXXXXXXXXXXXXXXXXGFSGLQGPAG

PPGSPGEQGPSGASGPAGPRXXXXXXXXXXXDGLNGLPGPIGPPGPRXXXXXXXXXXXXX

XXXXXXXXXXXXXXXXXXXXXXXXXXXXXXXXXXXXXXXXX?XXX?X?XXXXXXXXXXXX

XXXXXXXXXXXXXXXXXXXXXXXXXXXXXXXXXXXXXXXXXXXXXXXXXXXXXXXXXXXX

XXXXXXXXXXXXXXXXXXXXGHNGLDGLKGQPGAPGVKXXXXXXXXXXXXXXXXXXXXXX

XXXXxxxxxxxxxxXXXXXXXXXXXXXXXXXXXXXXXXXXXXXXGELGPVGNTGPSGPAG

PRXXXXXXXXXXXXXXXXXXXXXXXXXXXGAAGLPGVAGAPGLPGPRGIPGPVGASGATG

ARXXXXXXXXXXXXXXXXXXXXXXXXXXXXXXXXXXXXXXRGPSGESGSTGPTGPPGLRX

XXXXXxxxxxxxxxxxxxxxxxxGASGPAGVRxXXXXXXXXXXXXXXXXXXXXXXXXXXX

XXXXEGPAGLPGIDGRPGPIGPAGARGEAGNIGFPGPKXXXXXXXXXXXXGHAGLAGNRX

XXXXXXXXXXXXXXXXXXXXXXXXXXXXXXXXXXXXXXXXXXXXXXXXXXXXXGLPGEFG

LPGPAGPRXXXGAPGESGAVGPSGAIGSRXXXXXXXXXXXXXXXXXXXXXXXXXXXXXXX

XXXXXXXXXXXXXXXXXXXXXXXXXXXXXXXXXXXGAPGAVGAPGPAGATGDRGEAGAAG

PAGPAGPRXXXXXXGEVGPAGPNGFAGPAGAAGQPGAKGERXXXGPKGENGIVGPTGPVG

SAGPAGPNGPAGPAGSRGDGGPPGITGFPGAAGRXXXXXXXXXXXXXXXXXXXXXXXXXX

XXxxxxxxxxAGETGAGGPPGFTGEKGPSGEPGTAGPPGTAGPQGLLGAPGILGLPGSRX

XXGLPGVAGAVGEPGPLGIQGPPGARGPSGAVGGPGVNGAPGEAGRXXXXXXXXXXXXXX

XXXXXXXXXXXXXXXXXXXXXXXXXXXXXXXXXXHGNRGEPGPVGSAGPVGAIGPRGPSG

PQGIRXXXXXXXXXXXXXXXXXXxxxxxxxxxxxxxxxxxxxxxxxxxxxxxxXXXXXXX

XXXXXXXTGHPGAVGPAGIRXXXXXXXXXXXXXXXXXXXXXXXXXXXXXXXXXXXXXXX

>MEGATHERIUM_CONSENSUS_PMF

XXXXXXXXXXXX?XXXXXXXXXXXXXXXXXXXXXXXXXXXXXXXXXXXXXXXXXXXXXXX

XXXXXXXXXXXXXXXXXXXXXXXXXXXXXXXXxxxxxxxxxxxxxxxxxxxxxxxxGDAG

PAGPKGEPGSPGENGAPGQMGPRXXXXXXGRPGASGPAGARGNDGATGAAGPPGPTGPAG

PPGFPGAVGAKGEAGPQGARGSEGPQGVRGEPGPPGPAGAAGPAGNPGADGQPGAKGANG

APGIAGAPGFPGARxxxxxxxxxxxxxxxxxxxxxxxxxxxxxxxxxxxxxxxxxxxxxx

xxxxxxxxxxxGEPGPTGLPGPPGERGGPGSRGFPGADGVAGPKGPAGERXXXXXXXXXx

xxxxxxxxxxxxxxxxxxxxxxxxxxxxxxxTGPPGPAGQDGRPGPPGPPGARGQAGVMG

FPGPKxxxxxxxxxxxxxxxxxxxxxxxxxxxxxxxxxxxxxxxxxxxxxxxxxxxxxxx

xxxxxxxxxxxxxxxxxxxxxxxxxxxxxxxxxxxxxxxxxxxxGVQGPPGPAGPRXXXX

XXXXXXXXXXXXXXXXXXXXXXXXXXXXXXXXXXXXXXXXXXXXXXXXXXXXXXXXXXXX

XXGLTGPIGPPGPAGSPGDKGESGPSGPAGPTGARxxxxxxGEPGPPGPAGFAGPPGADG

QPGAKXXXXXXXXXGDAGPAGPAGPTGPPGPIGNVGAPGPKGARGSAGPPGATGFPGAAG

RVGPPGPSGNAGPPGPPGPVGKXXXXXXXXXXXXXXXXXXXXXXXXXXXXXXXGSPGADG

PAGAPGTPGPQGIAGQRGVVGLPGQRxxxGFPGLPGPSGEPGKQGPSGSSGERXXXXXXX

XXXXXXXXXXXXXXXXXXXXXXXXXXXXXXXXXXXXXXXXXXXXXXXXXXXXXXXXXXXX

XXXXXXXXXXXXXXXXXXXXXXXGPAGPQGPRXXXXXXXXXXXXXXXxxxGFSGLQGPAG

PPGSPGEQGPSGASGPAGPRXXXXXXXXXXXXXXXXXXXXXXXXXXXXXXXXXXXXXXXX

XXXXXXXXXXXXXXXXXXXXXXXXXXXXXXXXXXXXXXXXX?XXX?X?XXXXXXXXXXXX

XXXXXXXXXXXXXXXXXXXXXXXXXXXXXXXXXXXXXXXXXXXXXXXXXXXXXXXXxxxx

xxxxxGFPGTPGLPGFKxxxXXXXXXXXXXXXXXXXXXXXXXXXXXXXXXXXXXXXXXXX

XXxxVGAPGPAGARXXXXXXXXXXXXXXXXXXXXXXXXXXXXXXXXXXXXXXXXXXXXXX

XXXXXXXXXXXXXXXXXXXXXXXXXXXXXGAAGLPGVAGAPGLPGPRGIPGPAGASGATG

ARXXXXXXXXXXXXXXXXXXXXXXXXXXXXXXXXXXXXXXXXXXXXXXXXXXXXXXXXXX

XXxxxGLPGADGRAGVIGPAGARXXXXXXXXXXXXXXXXXXXXXXXXXXXGLPGSPGNVG

PAGKEGPAGLPGIDGRPGPIGPAGARGEAGNIGFPGPKXXXXXXXXXXXXXXXXXXXXXX

XXXXXXXXXXXXXXXXXXXXXXXXXXXXXXXXXXXXXXXXXXXXXXXXXXXXXGLPGEFG

LPGPAGPRGERGPPGESGAVGPSGAIGSRXXXXXXXXXXXXXXXXXXXXXXXXXXXXXXX

XXXXXXXXXXXXXXXXXXXXXXXXXXXXXXXXXXXXXXXXXXXXXXXXXXXXXxxxxxxx

xxxxxxxxxxxxxxGEVGPAGPNGFAGPAGAAGQPGAKGERXXXXXXXXXXXXXXXXXXX

XXXXXXXXXXXXXXXXXXXXXXXXXXXXXXXXXXTGPPGPSGITGPPGPPGAAGKxxxxx

xxxxxxxxxxXXXXXXXXXXXXXXXXXXXXXXXXXXXXXXXXXXXXXXXXXXXXXXXXXG

ERGLPGVAGAVGEPGPLGIQGPPGARXXXXXXXXXXXXXXXXXXXXXXXXXXXXXXXXXX

xxxxxxxxXXXXXXXXXXXXXXXXXXXXXXXXXXHGNRGEPGPVGSVGPVGAIGPRGPSG

PQGIRXXXXXXXXXXXXXXXXXXXXXXXXXXXXXXXXXXXXXXXXXXXXXXXXXXXXXXX

XXXXXXXXXXXXXXXXXXXXXXXXXXXXXXXXXXXXXXXXXXXXXXXXXXXXXXXXXXX

>LESTODON_CONSENSUS_PMF

XXXXXXXXXXXX?XXXXXXXXXXXXXXXXXXXXXXXXXXXXXXXXXXXXXXXXXXXXXXX

XXXXXXXXXXXXXXXXXXXXXXXxxxxxxxxxxxxxxxxxxxxxXXXGFSGLDGAKXXXX

XXXXXXXXXXXXXXXXXXXXXXXXXXXXXGRPGASGPAGARGNDGATGAAGPPGPTGPAG

PPGFPGAVGAKXXXXXXXXXGSEGPQGVRGEPGPPGPAGAAGPAGNPGADGQPGAKGANG

APGIAGAPGFPGARXXXXXXXXXXXXXXXXXXXXXXXXXXXXXXXXXGEPGPTGIQGPPG

PAGEEGKRXXXGEPGPTGLPGPPGERXXXXXXGFPGADGVAGPKXXXXXXXXXXXXXXXx

xxxxxxxPGEAGLPGAKXXXXXXXXXXXXXXxxxxxxxxxxxxxxxxxxxxxxGQAGVMG

FPGPKXXXXXXXXXXXXXXXXXXXXXXXXXXDGEAGAQGPPGPAGPAGERXXXXXXXXXX

XXXXXXXXXXXXXXXXXXXXXXXXXXXXXXXXXXXXXXXXXXXXGVQGPPGPAGPRXXXX

XXXXXXXXXXXXXXXXXXXXXXXXXXXXXXXXxxxxxxxxxxxxXXXXXXxxxxxxxxxx

xxGLTGPIGPPGPAGAPGDKGESGPSGPAGPTGARxxxxxxGEPGPPGPAGFAGPPGADG

QPGAKxxxxxxxxxGDAGPPGPAGPTGPPGPIGNVGAPGPKXXXGSAGPPGATGFPGAAG

RxxxxxxxxxxxxxxxxxxxxxXXXXXXXXXXXXXXXXXXXXXXXXXXXXXXXxxxxxxx

xxxxxxxxxxxxxxxxxGVVGLPGQRXXXGFPGLPGPSGEPGKQGPSGSSGERGPPGPVG

PPGLAGPPGESGRxxxxxxxxxxxxxxxxxxxGDRGESGPAGPPGAPGAPGAPGPVGPAG

KSGDRGETGPAGPAGPAGPAGARxxxxxxxxxXXXXXXXXXXXXXXXXXXGFSGLQGPAG

PPGSPGEQGPSGASGPAGPRXXXXXXXXXXXDGLNGLPGPIGPPGPRXXXXXXXXXXXXX

XXXXXXXXXXXXXXXXXXXXXXXXXXXXXXXXXXXXXXXXX?XXX?X?XXXXXXXXXXXX

XXXXXXXXXXXXXXXXXXXXXXXXXXXXXXXXXXXXXXXXXXXXXXXXXXXXXXXXXXXX

XXXXXXXXXXXXXXXXXXXXGHNGLDGLKGQPGAPGVKXXXXXXXXXXXXXXXXXXXXXX

XXXXxxxxxxxxxxXXXXXXXXXXXXXXXXXXXXXXXXXXXXXXGELGPVGNTGPSGPAG

PRXXXXXXXXXXXXXXXXXXXXXXXXXXXGAAGLPGVAGAPGLPGPRGIPGPVGASGATG

ARXXXXXXXXXXXXXXXXXXXXXXXXXXXXXXXXXXXXXXRGPSGESGSTGPTGPPGLRX

XXXXXxxxxxxxxxxxxxxxxxxxxxxxxxxxxXXXXXXXXXXXXXXXXXXXXXXXXXXX

XXXXEGPAGLPGIDGRPGPIGPAGARGEAGNIGFPGPKXXXXXXXXXXXXxxxxxxxxxX

XXXXXXXXXXXXXXXXXXXXXXXXXXXXXXXXXXXXXXXXXXXXXXXXXXXXXGLPGEFG

LPGPAGPRXXXGAPGESGAVGPSGAIGSRXXXXXXXXXXXXXXXXXXXXXXXXXXXXXXX

XXXXXXXXXXXXXXXXXXXXXXXXXXXXXXXXXXXGAPGAVGAPGPAGATGDRGEAGAAG

PAGPAGPRXXXXXXGEVGPAGPNGFAGPAGAAGQPGAKGERXXXGPKGENGIVGPTGPVG

SAGPAGPNGPAGPAGSRGDGGPPGITGFPGAAGRXXXXXXXXXXXXXXXXXXXXXXXXXX

XXxxxxxxxxAGETGAGGPPGFTGEKGPSGEPGTAGPPGTAGPQGLLGAPGILGLPGSRX

XXGLPGVAGAVGEPGPLGIQGPPGARGPSGAVGGPGVNGAPGEAGRXXXXXXXXXXXXXX

XXXXXXXXXXXXXXXXXXXXXXXXXXXXXXXXXXHGNRGEPGPVGSAGPVGAIGPRGPSG

PQGIRXXXXXXXXXXXXXXXXXXxxxxxxxxxxxxxxxxxxxxxxxxxxxxxxXXXXXXX

XXXXXXXTGHPGAVGPAGIRXXXXXXXXXXXXXXXXXXXXXXXXXXXXXXXXXXXXXXX

>BRADYPUS

XXXXXXXXXXXX?XXXXXXXXXXXXXXXXXXXXXXXXXXXXXXXXXXXXXXXXXXXXXXG

PPGPPGKXXXXXXXXXXXXXXXXGPPGPQGARGLPGTAGLPGMKGHRGFSGLDGAKGDAG

PAGPKGEPGSPGENGAPGQMGPRXXXXXXGRPGASGPAGARGNDGATGAAGPPGPTGPAG

PPGFPGAVGAKGEAGPQGARGSEGPQGVRGEPGPPGPAGAAGPAGNPGADGQPGAKGANG

APGIAGAPGFPGARGPSGPQGPSGpPGPKGNSGEPGAPGSKXXXXXXGEPGPTGIQGPPG

PAGEEGKRGARGEPGPTGLPGpPGERGGPGSRGFPGADGvAGPKXXXXXXXXXXXXXXXG

SPGEAGRPGEAGLPGAKGLTGSPGSPGPDGKTGPPGPAGQDGRPGPpGPPGARGQAGVMG

FPGPKXXXXXXXXXXXXGVPGPPGAVGPAGKDGEAGAQGPPGPAGPAGERXXXXXXXXXX

XXXXXXXXXXXXXXXXXXXXXXXXXXXXXXXXXXXXXXXXXXXXGVQGPPGPAGPRGSNG

APGNDGAKGDAGAPGAPGSQGAPGLQGMPGERGAAGLPGPKGDRGDAGPKXXXXXXXXXX

XXGLTGPIGPPGPAGAPGDKGEsGPSGPAGPTGARGAPGDRGEPGPPGPAGFAGPPGADG

QPGAKXXXXXXXXXGDAGPPGPAGPTGPPGPIGNVGAPGPKXXXGSAGPPGATGFPGAAG

RVGPPGPSGNAGPPGPPGPVGKEGGKGPRGETGPAGRPGEVGPPGPPGPTGEKGSPGADG

PAGAPGTPGPQGIAGQRGVVGLPGQRXXXGFPGLPGPSGEPGKQGPSGSSGERGPPGPVG

PPGLAGPPGESGREGsPGAEGSPGRXXXXXXXGDRGESGPAGPPGAPGAPGAPGPVGPAG

KNGDRGETGPAGPAGPAGPAGARGPAGPQGPRGDKGETGEAGDRXXXXXXGFSGLQGPaG

pPGSPGEQGPSGASGPAGPRGPPGSAGSPGKDGLNGLPGPIGPPGPRXXXXXXXXXXXXX

XXXXXXXXXXXXXXXXXXXXXXXXXXXXXXXXXXXXXXXXX?XXGVGLGPGPMGLMGPRX

XXXXXXXXXXXXXXXXXXXXXXXXXXXXXXXXXXXXXXXXXXXXXXXXXXXXXXXXGVVG

PQGARGFPGTPGLPGFKGIRGYNGLDGLKGQPGAAGVKGEPGAPGENGTPGQTGARXXXX

XXXXVGAPGPAGSRGSDGSVGPVGPAGPIGSAGPPGFPGAPGPKGELGPVGNTGPSGPAG

PRGEQGLPGVSGPVGPPGNPGANGLTGAKGAAGLPGVAGAPGLPGPRGIPGPVGASGATG

ARGLVGEPGPAGSKGESGGKGEPGSAGPQGPPGSSGEEGKRGPSGESGSTGPTGPPGLRX

XXXXXGLPGADGRAGVIGPAGARGASGPAGVRGPSGDTGRPGEPGLMGARGLPGSPGNVG

PAGKEGPAGLPGIDGRPGPIGPAGARGEAGNIGFPGPKXXXXXXXXXXXXXXXXXXXXXG

APGPDGNNGAQGPPGLQGVQGGKXXXXXXXXXXXXXXXXXXXXXXXXXXXXXXGLPGEFG

LPGPAGPRGERGPPGESGAVGPSGAIGSRXXXXXXXXXXXXGEPGVVGAPGTAGPAGSGG

LPGERGAAGIPGGKXXXGETGLRGEVGTTGRDGARGAPGAVGAPGPAGATGDRXXXXXXX

XXXXXXXXXXXXXXGEVGPAGPNGFAGPAGAAGQPGAKXXXXXXGPKGENGIVGPTGPVG

SAGPAGPNGPAGPAGSRGDGGPPGVTGFPGAAGRXXXXXXXXXXXXXXXXXXXXXXXXXX

XXGDQGPLGRAGETGAGGPPGFtGEKGPSGEPGTAGPPGTAGPQGLLGAPGILGLPGSRG

ERGLPGVAGAVGEPGPLGIQGPPGARGPSGAVGGPGVNGAPGEAGRdGNPGSDGPPGRXX

XXXXXXXXGYAGNPGPVGAAGAPGPHGAVGPAGKHGNRGEPGPVGSAGPVGAIGPRGPSG

PQGIRXXXXXXXXXXXXXXXXXXXXXXXXXXXXXXXXXXXXXXXXXXXXXXXXGPSGPSG

PPGKDGRTGHPGAVGPAGIRXXXXXXXXXXXXXXXXXXXXXXXXXXXXXXXXXXXXXXX

>CHOLOEPUS

XXXXXXXXXXXX?XXXXXXXXXXXXXXXXXXXXXXXXXXXXXXXXXXXXXXXXXXXXXXG

PPGPPGKNGDDGEAGKXXXXXXXGPPGPQGARGLPGTAGLPGMKGHRGFSGLDGAKGDAG

PAGPKGEPGSPGENGAPGQMGPRXXXXXXGRPGASGPAGARGNDGATGAAGPPGPTGPAG

PPGFPGAVGAKGEAGPQGARGSEGPQGVRGEPGPPGPAGAAGPAGNPGADGQPGAKGANG

APGIAGAPGFPGARGPSGPQGPSGPPGPKXXXXXXXXXXXXXXXXXXGEPGPTGIQGPPG

PAGEEGKRXXXGEPGPTGLPGpPGERXXXXXXGFPGADGVAGPKXXXXXXXXXXXXXXXG

SPGEAGRPGEAGLPGAKGLTGSPGSPGPDGKXXXXXXXXXXXXXXXXXXXXXXGQAGVMG

FPGPKXXXXXXXXAGERGVPGPPGAVGPAGKDGEAGAQGPPGPAGPAGERXXXXXXXXXX

XXXXXXXXXXXXXXXXXXXXXXXXXXXXXXXXXXXXXXXXXXXXGVQGPPGPAGPRGSNG

APGNDGAKGDAGAPGAPGSQGAPGLQGMPGERGAAGLPGPKGDRGDAGPKXXXXXXXXXX

XXGLTGPIGPPGPAGAPGDKGESGPSGPAGPTGARGAPGDRGEPGPPGPAGFAGPPGADG

QPGAKXXXXXXXXXGDAGPPGPAGPTGPPGPIGNVGAPGPKXXXGSAGPPGATGFPGAAG

RVGPPGPSGNAGPPGPPGPVGKXXXXXXXGETGPAGRPGEVGPPGPPGPTGEKGSPGADG

PAGAPGTPGPQGIAGQRGVVGLPGQRXXXGFPGLPGPSGEPGKQGPSGSSGERGPPGPVG

PPGLAGPPGESGREGSPGAEGSPGRXXXXXXXXXXGESGPAGPPGAPGAPGAPGPVGPAG

KNGDRGETGPAGPAGPAGPAGARGPAGPQGPRGDKGETGEAGDRXXXXXXGFSGLQGPaG

pPGSPGEQGPSGASGPAGPRGPPGSAGSPGKDGLNGLPGPIGPPGPRXXXXXXXXXXXXX

XXXXXXXXXXXXXXXXXXXXXXXXXXXXXXXXXXXXXXXXX?XXGVGLGPGPMGLMGPRX

XXXXXXXXXXXXXXXXXXXXXXXXXXXXXXXXGPAGPPGKXXXXXXXXXXXXXXXXXXXX

XXXXXGFPGTPGLPGFKGIRGYNGLDGLKGQPGAAGVKGEPGAPGENGTPGQTGARXXXX

XXXXVGAPGPAGSRGSDGSVGPVGPAGPIGSAGPPGFPGAPGPKGELGPVGNTGPSGPAG

PRGEQGLPGVSGPVGPPGNPGANGLTGAKGAAGLPGVAGAPGLPGPRGIPGPVGASGATG

ARGLVGEPGPAGSKGESGGKGEPGSAGPQGPPGSSGEEGKRGPSGESGSTGPTGPPGLRX

XXXXXXXXXXXXXAGVIGPAGARGASGPAGVRXXXXXXXXXXXXXXXXXXGLPGSPGNVG

PAGKEGPAGLPGIDGRPGPIGPAGARGEAGNIGFPGPKXXXXXXXXXXXXXXXXXXXXXG

APGPDGNNGAQGPPGLQGVQGGKXXXXXXXXXXXXXXXXXXXXXXXXXXXXXXGLPGEFG

LPGPAGPRGERGPPGESGAVGPSGAIGSRGPSGPPGPDGNKGEPGVVGAPGTAGPAGSGG

LPGERXXXXXXXXXXXXXXXXXXXXXXXXXXXXXXGAPGAVGAPGPAGATGDRGEAGAAG

PAGPAGPRXXXXXXGEVGPAGPNGFAGPAGAAGQPGAKGERXXXGPKGENGIVGPTGPVG

SAGPAGPNGPAGPAGSRGDGGPPGVTGFPGAAGRTGPPGPSGITGPPGPSGAAGKXXXXX

XXGDQGPLGRAGETGAGGPPGFTGEKXXXXXXXXXXXXXXXXXXXXXXXXXXXXXXXXXX

XXGLPGVAGAVGEPGPLGIQGPPGARGPSGAVGGPGVNGAPGEAGRDGNPGSDGPPGRXX

XXXXXXXXGYAGNPGPVGAAGAPGPHGAVGPAGKHGNRGEPGPVGSAGPVGAIGPRGPSG

PQGIRXXXGEAGDKGPRGLPGLKGHNGLQGLPGLAGQHGDQGAPGSVGPAGPRGPSGPSG

PPGKDGRTGHPGAVGPAGIRXXXXXXXXXXXXXXXXXXXXXXXXXXXXXXXXXXXXXXX

>EQUUS

QLSYGYDEKSAG-ISVPGPMGPSGPRGLPGPPGAPGPQGFQGPPGEPGEPGASGPMGPRG

PPGPPGKNGDDGEAGKPGRPGERGPPGPQGARGLPGTAGLPGMKGHRGFSGLDGAKGDAG

PAGPKGEPGSPGENGAPGQMGPRGLPGERGRPGAPGPAGARGNDGATGAAGPPGPTGPAG

PPGFPGAVGAKGEAGPQGARGSEGPQGVRGEPGPPGPAGAAGPAGNPGADGQPGAKGANG

APGIAGAPGFPGARGPSGPQGPSGPPGPKGNSGEPGAPGNKGDTGAKGEPGPTGIQGPPG

PAGEEGKRGARGEPGPTGLPGPPGERGGPGARGFPGADGVAGPKGPAGERGAPGPAGPKG

SPGEAGRPGEAGLPGAKGLTGSPGSPGPDGKTGPPGPAGQDGRPGPPGPPGARGQAGVMG

FPGPKGAAGEPGKAGERGVPGPPGAVGPAGKDGEAGAQGPPGPAGPAGERGEQGPAGSPG

FQGLPGPAGPPGESGKPGEQGVPGDLGAPGPSGARGERGFPGERGVQGPPGPAGPRGSNG

APGNDGAKGDAGAPGAPGSQGAPGLQGMPGERGAAGLPGPKGDRGDAGPKGADGSPGKDG

VRGLTGPIGPPGPAGAPGDKGETGPSGPAGPTGARGAPGDRGEPGPPGPAGFAGPPGADG

QPGAKGEPGDAGAKGDAGPPGPAGPAGPPGPIGSVGAPGPKGARGSAGPPGATGFPGAAG

RVGPPGPSGNAGTPGPPGPVGKEGGKGPRGETGPAGRPGEAGPPGPPGPSGEKGSPGADG

PAGAPGTPGPQGIAGQRGVVGLPGQRGERGFPGLPGPSGEPGKQGPSGASGERGPPGPVG

PPGLAGPPGESGREGAPGAEGSPGRDGSPGPKGDRXXXXXXXXXXXXXXXXXXXXXXXXX

XXXXXXXXGPAGPAGPIGPVGARGPAGPQGPRGDKGETGEQGDRGIKGHRGFSGLQGPPG

PPGSPGEQGPSGASGPAGPRGPPGSAGAPGKDGLNGLPGPIGPPGPRGRTGDAGPVGPPG

PPGPPGPPGPPSGGFDFSFLPQPPQEKSHDGGRYYRARQFDA-KG-G-GPGPMGLMGPRG

PPGASGAPGPQGFQGPAGEPGEPGQTGPAGARGPPGPPGKAGEDGHPGKPGRPGERGVVG

PQGARGFPGTPGLPGFKGIRGHNGLDGLKGQPGAPGVKGEPGAPGENGTPGQAGARGLPG

ERGRVGAPGPAGARGSDGSVGPVGPAGPIGSAGPPGFPGAPGPKGELGPVGNPGPAGPAG

PRGEVGLPGLSGPVGPPGNPGANGLTGAKGAAGLPGVAGAPGLPGPRGIPGPAGAAGATG

ARGLVGEPGPAGSKGESGNKGEPGAAGPQGPPGPSGEEGKRGPNGEPGSTGPAGPPGLRG

SPGSRGLPGADGRAGVMGPAGSRGASGPAGVRGPNGDSGRPGEPGLMGPRGFPGSPGNIG

PAGKEGPVGLPGIDGRPGPIGPAGARGEPGNIGFPGPKGPSGEPGKPGDKGHAGLAGARG

APGPDGNNGAQGPPGPQGVQGGKGEQGPAGPPGFQGLPGPAGTAGEVGKPGERGLPGEFG

LPGPAGARGERGPPGESGAAGPAGPIGSRGPSGPPGPDGNKGEPGVLGAPGTAGPSGPSG

LPGERGAAGIPGGKGEKGETGLRGEIGNPGRDGARGAPGAVGAPGPAGANGDRGEAGAAG

PAGPAGPRGSPGERGEVGPAGPNGFAGPAGAAGQPGAKGERGTKGPKGENGPVGPTGPVG

AAGPSGPNGPPGPAGSRGDGGPPGVTGFPGAAGRTGPPGPSGISGPPGPPGAAGKEGLRG

PRGDQGPVGRAGETGASGPPGFAGEKGPSGEPGTAGPPGTPGPQGLLGAPGILGLPGSRG

ERGLPGVAGSLGEPGPLGIAGPPGARGPPGAVGAPGVNGAPGEAGRDGNPGSDGPPGRDG

QPGHKGERGYPGNAGPVGAVGAPGPHGPVGPTGKHGHRGEPGPVGSVGPVGAVGPRGPSG

PQGVRGDKGEPGDKGPRGLPGLKGHNGLQGLPGLAGQHGDQGAPGSVGPAGPRGPAGPTG

PVGKDGRSGQPGTVGPAGVRGSQGSQGPAGPPGPPGPPGPPGPSGGGYDFGYDGDFYRA

>CERATOTHERIUM

XXXXXXXXXXXX?XXXXXXXGPSGPRGLPGPPGAPGPQGFQGPPGEPGEPGASGPMGPRG

PPGPPGKNGDDGEAGKPGRPGERGPPGPQGARGLPGTAGLPGMKGHRGFSGLDGAKGDAG

PAGPKGEPGSPGENGAPGQMGPRGLPGERGRPGAPGPAGARGNDGATGAAGPPGPTGPAG

PPGFPGAVGAKGEAGPQGARGSEGPQGVRGEPGPPGPAGAAGPAGNPGADGQPGAKGANG

APGIAGAPGFPGARGPSGPQGPSGPPGPKGNSGEPGAPGSKGDTGAKGEPGPTGIQGPPG

PAGEEGKRGARGEPGPTGLPGPPGERXXXXXXXXXXXXXXXXXXGPAGERGAPGPAGPKG

SPGEAGRPGEAGLPGAKGLTGSPGSPGPDGKTGPPGPAGQDGRPGPPGPPGARGQAGVMG

FPGPKGAAGEPGKAGERGVPGPPGAVGPAGKDGEAGAQGPPGPAGPAGERGEQGPAGSPG

FQGLPGPAGPPGESGKPGEQGVPGDLGAPGPSGARGERGFPGERGVQGPPGPAGPRGANG

APGNDGAKGDAGAPGAPGSQGAPGLQGMPGERGAAGLPGPKGDRGDAGPKGADGAPGKDG

VRGLTGPIGPPGPAGAPGDKGESGPSGPAGPTGARGAPGDRGEPGPPGPAGFAGPPGADG

QPGAKGEPGDAGAKGDAGPPGPAGPTGPPGPIGSVGAPGPKGARGSAGPPGATGFPGAAG

RVGPPGPSGNAGPPGPPGPVGKEGGKGPRGETGPAGRPGEAGPPGPPGPSGEKGSPGADG

PAGAPGTPGPQGIAGQRGVVGLPGQRGERGFPGLPGPSGEPGKQGPSGASGERGPPGPVG

PPGLAGPPGESGREGAPGAEGSPGRDGSPGPKGDRGETGPAGPPGAPGAPGAPGPVGPAG

KSGDRGETGPAGPAGPVGPVGARGPAGPQGPRGDKGETGEQGDRGIKGHRGFSGLQGPPG

PPGSPGEQGPSGASGPAGPRGPPGSAGAPGKDGLNGLPGPIGPPGPRGRTGEAGPVGPPG

PPGPPGPPGPPSAGFDFSFLPQPPQEKAHDGGRYYRARXXXX?XX?X?XXXXXGLMGPRG

PPGASGAPGPQGFQGPAGEPGEPGQTGPAGARGPPGPPGKAGEDGHPGKPGRPGERGVVG

PQGARGFPGTPGLPGFKGIRGHNGLDGLKGQPGAPGVKGEPGAPGENGTPGQXGARGLPG

ERGRVGAPGPAGARGSDGSVGPVGPAGPIGSAGPPGFPGAPGPKGELGPVGNPGPAGPAG

PRGEVGLPGLSGPVGPPXXXXXXXXXXXXXXXGLPGVAGAPGLPGPRGIPGPAGAAGATG

ARGLVGEPGPAGSKGESGNKGEPGSVGAQGPPGPSGEEGKRGPNGEAGSTGPAGPPGLRG

SPGSRGLPGADGRAGVMGLAGSRGATGPAGARGPSGDSGRPGEPGLMGPRXXXXXXXXXX

XXXXXXXXGLPGIDGRPGPVGPAGARGEPGNIGFPGPKGPXXXXXXXXXXXXXXXXXXXG

APGPDGNNGAQGPPGPQGVQGGKGEQGPAGPPGFQGLPGPAGTAGEVGKPGERXXXXXXX

XXXXXXXXGERGPPGESGAAGPAGPIGSRGPSGAPGPDGNKGEPGVLGAPGTAGPSGPSG

LPGERGAAGIPGGKGEKGETGLRGEIGNSGRDGARGPPGAVGAPGPAGANGDRXXXXXXX

XXXXXXXXXXXGERGEVGPAGPNGFAGPAGAAGQPGAKGERGTKGPKGEIGPVGPTGAVG

SAGPSGPNGPPGPAGSRGDGGPPGATGFPGAAGRTGPPGPSGITGPPGPPGAAGKEGVRG

PRGDQGPVGRAGETGSSGPPGFAGEKGPSGEPGTAGPPGTPGPQGLLGAPGILGLPGSRG

ERGLPGVAGSXGEPGPLGIAGPPGARGPPGAVGAPGVNGAPGETGRDGNPGNDGPPGRDG

QPGHKGERGYPGNAGPVGAVGAPGSHGPVGPTGKHGNRGEPXXXXXXXXXXXXXXXXXXG

PQGVRGDKGEPGDKGPRGLPGLKGHGGLQGLPGLAGHHGDQGAPGSVGPAGPRGPAGPTG

PVGKDGRSGQPGTVGPAGVRGSQGSQGPAXXXXXXXXXXXXXXXXXXXXXXXXXXXXXX

>VICUGNA

QMSYGYDEKSTG-ISVPGPMGPSGPRGLPGPPGAPGPQGFQGPPGEPGEPGSSGPMGPRG

PPGPPGKNGDDGEAGKPGRPGERGPPGPQGARGLPGTAGLPGMKGHRGFSGLDGAKGDAG

PAGPKGEPGSPGENGAPGQMGPRGLPGERGRPGAPGPAGARGNDGATGAAGPPGPTGPAG

PPGFPGAVGAKGEAGPQGARGSEGPQGVRGEPGPPGPAGAAGPAGNPGADGQPGAKGANG

APGIAGAPGFPGARGPSGPQGPSGPPGPKGNSGEPGAPGNKGDTGAKGEPGPTGVQGPPG

PAGEEGKRGARGEPGPAGLPGPPGERGGPGSRGFPGADGVAGPKGPAGERGSPGPAGPKG

SPGEAGRPGEAGLPGAKGLTGSPGSPGPDGKTGPPGPAGQDGRPGPPGPPGARGQAGVMG

FPGPKGAAGEPGKAGERGVPGPPGAVGPAGKDGEAGAQGPPGPAGPAGERGEQGPAGSPG

FQGLPGPAGPPGEAGKPGEQGVPGDLGAPGPSGARGERGFPGERGVQGPPGPAGPRGANG

APGNDGAKGDAGAPGAPGSQGAPGLQGMPGERGAAGLPGPKGDRGDAGPKGADGSPGKDG

VRGLTGPIGPPGPAGAPGDKGETGPSGPAGPTGARGAPGDRGEPGPPGPAGFAGPPGADG

QPGAKGEPGDAGAKGDAGPPGPAGPTGPPGPIGSVGAPGPKGARGSAGPPGATGFPGAAG

RVGPPGPSGNAGPPGPPGPVGKEGSKGPRGETGPAGRPGEVGPPGPPGPAGEKGAPGADG

PAGAPGTPGPQGIAGQRGVVGLPGQRGERGFPGLPGPSGEPGKQGPSGPNGERGPPGPMG

PPGLAGPPGESGREGAPGAEGSPGRDGSPGAQGDRGETGPAGPPGAPGAPGAPGPVGPAG

KSGDRGETGPAGPAGPIGPVGARGPAGPQGPRGDKGETGEQGDRGIKGHRGFSGLQGPPG

PPGSPGEQGPSGASGPAGPRGPPGSAGAPGKDGLNGLPGPIGPPGPRGRTGDAGPXXXXX

XXXPLGPPGPPSGGFDFSFLPQPPQEKXXXXXXXXXXRQFDG-KGXXXGPGPMGLMGPRG

PPGAAGAPGPQGFQGPAGEPGEPGQTGPAGARGPPGPPGKAGEDGHPGKPGRPGERGVVG

PQGARGFPGTPGLPGFKGIRGHNGLDGLKGQPGAPGVKGEPGAPGENGTPGQTGARGLPG

ERGRVGAPGPAGARGSDGSVGPVGPAGPIGSAGPPGFPGAPGPKGELGPVGNPGPAGPAG

PRGEVGLPGVSGPVGPPGNPGANGLTGAKGAAGLPGVAGAPGLPGPRGIPGPTGAAGATG

ARGLVGEPGPAGSKGESGNKGEPGAAGPQGPPGPSGEEGKRGPTGEVGSPGPAGPPGLRG

NPGSRGLPGADGRAGVMGPAGSRGATGPAGVRGPNGDSGRPGEPGLMGPRGFPGSPGNIG

PAGKEGPVGLPGIDGRPGPIGPAGARGEPGNIGFPGPKGPAGEPGKHGEKGHAGLAGARG

APGPDGNNGAQGPPGPQGVQGGKGEQGPAGPPGFQGLPGPAGTAGEVGKPGERGLPGEFG

LPGPAGPRGERGPPGESGAAGPAGPIGSRGPSGPPGPDGNKGEPGVLGAPGTAGPSGPSG

LPGERGAAGIPGGKGEKGETGLRGDVGSPGRDGARGAPGAVGAPGPAGANGDRGEAGPAG

AAGPAGPRGSPGERGEVGPAGPNGFAGPAGAAGQPGAKGERGTKGPKGENGPVGPTGPVG

AAGPSGPNGPPGPAGSRGDGGPPGATGFPGAAGRTGPPGPSGISGPPGPPGPAGKEGLRG

PRGDQGPVGRAGETGASGPPGFAGEKGPSGEPGTAGPPGTPGPQGLLGAPGFLGLPGSRG

ERGLPGVAGAVGEPGPLGISGPPGARGPPGGVGSPGVNGAPGEAGRDGNPGSDGPPGRDG

QPGHKGERGYPGNAGPTGVVGAPGPQGPVGPAGKHGNRGEPGAAGSVGPTGAIGPRGPSG

PQGIRGDKGEPGDKGPRGLPGLKGHNGLQGLPGLAGHHGDQGAPGPVGPAGPRGPAGPSG

PAGKDGRSGHPGTVGPAGIRGSQGSQGPAGPPGPPGPPGPPGPSGGGYDFGYDGDFYRA

>PTEROPUS

QMSYGYDEKSAG-VSVPGPMGPSGPRGLPGPPGAPGPQGFQGPPGEPGEPGASGPMGPRG

PPGPPGKNGDDGEAGKPGRPGERGPPGPQGARGLPGTAGLPGMKGHRGFSGLDGAKGDSG

PAGPKGEPGSPGENGAPGQMGPRGLPGERGRPGAPGPAGARGNDGATGAAGPPGPTGPAG

PPGFPGAVGAKGEAGPQGSRGSEGPQGVRGEPGPPGPAGAAGPAGNPGADGQPGAKGANG

APGIAGAPGFPGARGPSGPQGPGGPPGPKGNSGEPGAPGNKGDAGAKGEPGPTGIQGPPG

PAGEEGKRGARGEPGPSGLPGPPGERGGPGSRGFPGADGVAGPKGPAGERGSPGPAGPKG

SPGEAGRPGEAGLPGAKGLTGSPGSPGPDGKTGPAGPAGQDGRPXPPGPPXARGQAGVMG

FPGXXXXXGEPGKAGERGVPGPPGAVGAAGKDGEAGAQGPPGPAGPAGERGEQGPAGSPG

FQGLPGPSGPPGEAGKPGEQGVPGDLGAPGPSGARGERGFPGERGVQGPPGPAGPRGANG

APGNDGAKGDAGAPGAPGSQGAPGLQGMPGERGAAGLPGPKGDRGDAGPKGADGAPGKDG

VRGLTGPIGPPGPAGAPGDKGESGPSGPAGPTGARGAPGDRGEPGPPGPAGFAGPPGADG

QPGAKGEPGDAGAKGDAGPAGPAGPAGPPGPIGNVGAPGPKGARGSAGPPGATGFPGAAG

RVGPPGPSGNAGPPGPPGPVGKEGGKGPRGETGPAGRPGEAGPPGPPGPAGEKGSPGADG

PAGAPGTPGPQGIAGQRGVVGLPGQRGERGFPGLPGPSGEPGKQGPSGTSGERGPPGPMG

PPGLAGPPGESGREGSPGAEGSPGRDGSPGPKGDRGETGPAGAPGAPGAPGAPGPVGPAG

KSGDRGETGPAGPAGPVGPVGARGPTGPQGPRGDKGETGEQGDRGIKGHRGFSGLQGPPG

PPXXXXXXXXXXXXXXXXXXGPPGSAGAAGKDGLNGLPGPIGPPGPRGRTGDAGPVGPPG

PPGPPGPPGPPSGGFDFSFLPQPPQEKAHDGGRYYRARQYDG-KGVGLGPGPMGLMGPRG

PPGAAGAPGPQGFQGPAGEPGEPGQTGPAGARGPTGPPGKAGEDGHPGKPGRPGERGVVG

PQGARGFPGTPGLPGFKGIRGHNGLDGLKGQPGAPGIKXXXXXXXXXXXXXXXGARGLPG

ERGRVGAPGPAGARGSDGSVGPVGPAGPIGSAGPPGFPGAPGPKGELGPVGNPGPAGPAG

PRGEVGLPGLSGPVGPPGNPGANGLTGAKGAAGLPGVAGAPGLPGPRGIPGPPGAVGATG

ARGLVGEPGPAGSKGESGNKGEPGSAGAQGPPGPSGEEGKRGSNGEAGSAGPPGPPGLRG

SPGSRGLPGADGRAGVMGPAGSRGATGPAGVRGPSGDSGRXXXXGLMGPRGFPGSPGNVG

PAGKEGPMGLPGIDGRPGPIGPAGARGEPGNIGFPGPKGPTGDPGKSGEKGHAGLAGPRG

APGPDGNNGAQGPPGLQGVQGGKGEQGPAGPPGFQGLPGPAGTTGEVGKPGERGLPGEFG

LPGPAGPRGERGPPGESGAVGPSGPIGSRGPSGPPGPDGNKGEPGGVGAPGTAGASGSGG

LPGERGAAGIPGGKGEKGETGLRGEVGSTGRDGARGAPGAIGAPGPAGATGDRGEAGPAG

PAGPAGPRGSPGERGEVGPAGPNGFAGPAGAAGQPGAKGERGTKGPKGENGPVGPTGPAG

SSGPAGPNGPPGPAGSRGDGGPPXXXXXXGAAGRTGPSGPSGITGPPGPPGAAGKEGVRG

PRGDQGPVGRTGETGAGGPPGFTGEKGPSGEPGTAGPPGTPGPQGLLGAPGILGLPGSRG

ERGLPGVAGSVGEPGPLGISGPPGARGPPGAVGNPGVNGAPGEAGRDGNPGNDGPPGRDG

QPGHKGERGYPGNPGPVGALGAPGPHGPVGPTGKHGNRGEPGPAGSVGPTGAVGPRGPSG

PQGIRGDKGEPGDKGPRGLPGLKGHNGLQGLPGLAGHHGDQGSPGSVGPAGPRGPAGPSG

PAGKDGRTGHPGTVGPAGIRGSQGSQGPAGPPGPPGPPGPPGVSGGGYDFGFDGDFYRA

>ERINACEUS

QMSYGYDEKSTGGMSVPGPMGPSGPRGLPGPPGSPGPQGFQGPPGEPGEPGASXXXXXXX

XXXXXXXXXXXGEAGKPGRPGERGPPGPQGARGLPGTAGLPGMKGHRGFSGLDGAKGDSG

PAGPKGEPGSPGENGAPGQMGPRGLPGERGRPGATGPAGARGNDGATGAAGPPGPTGPAG

PPGFPGAVGAKGEAGPQGARGSEGPQGVRGEPGPPGPAGAAGPAGNPGADGQPGAKGANG

APGIAGAPGFPGARGPSGPQGPSGPPGPKGNSGEPGAPGNKGDTGAKGEPGPAGVQGPPG

PAGEEGKRGARGEPGPTGLPGPPGERGGPGSRGFPGSDGAAGPKGPAGERGSPGPAGPKG

SPGEAGRPGEAGLPGAKGLTGSPGSPGPDGKTGPPGPAGQDGRPGPPGPPGARGQAGVMG

FPGPKGAAGEPGKAGERGVPGPPGAVXXXXXXXXXXXXXXXXXXGPAGERGEQGPAGSPG

FQGLPGPAGPPGEAGKPGEQGAPGDLGAPGPSGARGERGFPGERGVQGPPGPAGPRGSNG

APGNDGAKGDAGAPGAPGSQGAPGLQGMPGERGAAGLPGPKGDRGDAGPKGADGSPGKDG

VRGLTGPIGPPGPAGAPGDKGESGPSGPAGPTGARGAPGDRGEPGPPGPAGFAGPPGADG

QPGAKGEPGDAGAKGDSGPPGPAGPTGPPGPIGNVGAPGPKGARGAAGPPGATGFPGAAG

RVGPPGPSGNAGPPGPPGPVGKEGGKGPRGETGPAGRPGEAGPPGPPGPAGEKGSPGADG

PAGSPGTPGPQGIAGQRGVVGLPGQRGERGFPGLPGPSGEPGKQGPSGASGERGPPGPMG

PPGLAGPPGESGREGSPGAEGSPGRDGSPGPKGDRGETGPAGPPGAPGAPGAPGPVGPAG

KSGDRGETGPAGPAGPIGPAGARGPAGPQGPRGDKGETGEQGDRGMKGHRGFSGLQGPPG

PPGSPGEQGPSGASGPAGPRGPPGSAGAAGKDGLNGLPGPIGPPGPRGRTGDAGPXGPPG

PPGPPGPPGPPSGGFDLNFLPQPPQEKAHDGGRYYRARQYDG-KGVGLGPGPMGLMGPRG

PPGASGAPGPPGFQGPAGEPGEPGQTGPAGARGPTGPPGKAGEDGHPGKPGRPGERGVVG

PQGARGFPGTPGLPGFKGIRGHNGLDGLKGQPGAPGVKGEPGAPGENGTPGQTGARGLPG

ERGRVGAPGPAGARGSDGSVGPVGPAGPIGSAGPPGFPGAPGPKGELGPVGNPGPSGPAG

ARGEVGLPGVSGPVGPPGNPGANGLTGAKGAAGLPGVAGAPGLPGPRGIPGPVGAAGASG

ARGLVGEPGPAGSKGETGNKGEPGSAGAQGLPGPSGEEGKRGQNGEAGSAGPAGPPGLRG

SPGSRGLPGADGRPGVMGPPGSRGASGPAGVRGPSGDSGRPGEPGLMGPRGFPGSPGNVG

PAGKEGPSGLPGIDGRPGPIGPAGARGEPGNIGFPGPKGPSGDPGKSGDKGHAGLAGARG

APGPDGNNGAQGPPGAQGVQGGKGEQGPAGPPGFQGLPGPAGTTGEVGKPGERGLPGEFG

LPGPAGPRGERGPPGQSGAAGPSGPIGSRGPSGSPGPDGNKGEPGVLGAPGTAGPSGPGG

LPGERGAAGVPGGKGEKGETGLRGEIGNPGRDGARXXXXXXXXXXXXXXXGDRGEAGPAG

PAGPAGPRGSPGERGEVGPAGPNGFAGPAGAAGQPGAKGERGTKGPKGENGIVGPTGPVG

AAGPSGPNGPPGPAGGRGDGGPPGATGFPGAAGRTGPPGPSGITGPPGPPGAAGKEGLRG

PRGDQGPVGRTGETGGSGPPGFTGEKGPAGEPGTAGPPGTAGPQGLLGAPGILGLPGSRG

ERGLPGVFGSVGEPGPLGIAGPPGARGPPGAVGNPGVNGAPGEAGRDGNPGSDGPPGRDG

QPGHKGERGYPGNAGSVGAAGAPGPHGSVGPAGKHGNRGEPGPAGAVGPVGAFGPRGPSG

PQGIRGDKGEPGDKGPRGLPGLKGHNGLQGLPGLAGQHGDQGAPGSVGPAGPRGPAGPSG

PAGKDGHNGQPGTVGPAGIRGSQGNQGPAGPAGPPGPPGPPGPSGGGYDFGYEGDFYRA

>SOREX

XXXXXXXXXXXX?XXXXXXXGPSGPRGLPGPPGAPGPQGFQGPPGEPGEPGASXXXXXXX

XXXXXXXXXXXGEAGKPGRPGERGPPGPQGARGLPGTAGLPGMKGHRGFSGLDGAKGDSG

PAGPKGEPGSPGENGAPGQMGPRGLPGERGRPGAPGPAGARGNDGATGAAGPPGPTGPAG

PPGFPGAVGAKGEAGPQGARGSEGPQGVRGEPGPPGPAGAAGPAGNPGADGQPGAKGANG

APGIAGAPGFPGARGPSGPQGPSGPPGPKGNSGEPGAPGNKGDTGAKGEPGPAGVQGPPG

PAGEEGKRGARGEPGPTGLPGPPGERXXXXXXXXXXXXXXXXXXGPAGERGSPGPAGPKG

SPGEAGRPGEAGLPGAKGLTGSPGSPGPDGKTGPPGPAGQDGRPGPPGPPGARGQAGVMG

FPGPKGAAGEPGKAGERGVPGPPGAVXXXXXXXXXXXXXXXXXXGPAGERGEQGPAGSPG

FQGLPGPAGPPGEAGKPGEQGAPGDLGAPGPSGARGERGFPGERGVQGPPGPAGPRGSNG

APGNDGAKGDAGAPGAPGSQGAPGLQGMPGERGAAGLPGPKGDRGDAGPKGADGSPGKDG

VRGLTGPIGPPGPAGAPGDKGESGPSGPAGPTGARGAPGDRGEPGPPGPAGFAGPPGADG

QPGAKGEPGDAGAKGDAGPPGPAGPTGAPGPIGNVGAPGPKGARGSAGPPGATGFPGAAG

RVGPPGPSGNAGPPGPPGPVGKEGGKGPRGETGPAGRPGEVGPPGPPGPAGEKGSPGADG

PAGSPGTPGPQGIAGQRGVVGLPGQRGERGFPGLPGPSGEPGKQGPSGSSGERGPPGPMG

PPGLAGPPGESGREGSPGAEGSPGRDGSPGPKGDRGETGPAGPPGAPGAPGAPGPVGPAG

KSGDRGETGPAGPAGPIGPAGARGPAGPQGPRGDKGETGEQGDRGMKGHRGFSGLQGPPG

PPGSPGEQGPSGASGPAGPRGPPGSAGAAGKDGLNGLPGPIGPPGPRGRTGDAGPVGPPG

PPGPPGPPGPPSGGFDFSFMPQPPQEKAHDGGRYYRARQYDG-KGVGLGPGPMGLMGPRG

PPGASGAPGPQGFQGPAGEPGEPGQTGPAGARGPPGPPGKAGEDGHPGKAGRPGERGVVG

PQGARGFPGTPGLPGFKGIRGHNGLDGLKGQPGAPGVKGEPGAPGENGTPGQAGARGLPG

ERGRVGAPGPAGARGSDGSVGPVGPAGAIGSAGPPGFPGAPGPKGELGAVGNPGPAGPAG

ARGEVGLPGVSGPVGPAGNPGANGLTGAKGAAGLPGVAGAPGLPGPRGIPGPVGAAGASG

PRGLIGEPGPAGSKGEGGNKGEPGSAGPQGPPGPSGEEGKRGQNGEPGSAGPTGPPGLRG

TPGSRGLPGADGRPGVMGPPGSRGASGPAGARGPNGDSGRPGEPGLVGPRGFPGSPGSVG

PAGKEGPVGLPGIEGRPGAIGPAGARGEPGNIGFPGPKGPNGEPGKSGDKGHPGLAGARG

APGPDGNNGAQGPPGPQGVQGGKGEQGPAGPPGFQGLPGPAGTTGEVGKPGERGLPGEFG

LPGPAGPRGERGPPGESGAAGPAGPIGSRGPSGPPGPDGNKGEPGVVGAPGNAGPSGPGG

LPGERGAAGIPGGKGEKGETGLRGEIGNPGRDGARGAPGAVGAPGPSGAAGDRGEAGAAG

PAGPAGPRGSPGERGEVGPAGPNGFAGPAGAAGQPGAKGERGTKGPKGENGVVGPTGPVG

AAGPSGPNGPPGPAGSRGDGGPPGATGFPGAAGRTGPPGPSGITGPPGPPGAAGKEGLRG

PRGDQGPVGRTGETGASGLPGFAGEKGPNGEPGTAGPPGTPGPQGLLGAPGILGLPGSRG

ERGLPGIAGSVGEPGPLGISGPPGARGPPGAVGNPGVNGAPGEAGRDGNPGSDGPPGRDG

QPGHKGERGYPGNAGPVGAVGAPGPHGPVGPTGKHGNRGEPGPAGAVGPAGAFGPRGPSG

PQGIRGDKGEPGDKGARGLPGLKGHNGLQGLPGLAGHHGDQGAPGSVGPAGPRGPAGPSG

PAGKDGRSGHPGTVGPAGIRGSQGNQGPAGPPGPPGPPGPPGPSGGGYDFGYDGDFYRA

>MYOTIS

QMSYGYDEKSAG-VSVPGPMGPSGPRGLPGPPGSPGPQGFQGPPGEPGEPGASGPMGPRG

PPGPPGKNGDDGEAGKPGRPGERGPPGPQGARGLPGTAGLPGMKGHRGFSGLDGAKGDAG

PAGPKGEPGSPGENGVPGQMGPRGLPGERGRPGAPGPAGARGNDGATGAAGPPGPTGPAG

PPGFPGAVGAKGEAGPQGSRGSEGPQGVRGEPGPPGPAGAAGPAGNPGADGQPGAKGANG

APGIAGAPGFPGARGPSGPQGPSGAPGPKGNSGEPGAPGNKGDTGAKGEPGPTGIQGPPG

PAGEEGKRGARGEPGPAGLPGPPGERGGPGSRGFPGADGVAGPKGPAGERGSPGPAGPKG

SPGEAGRPGEAGLPGAKGLTGSPGSPGPDGKTGPTGPAGQDGRPGPPGPPGARGQAGVMG

FPGPKGAAGEPGKAGERGVPGPPGAVGPAGKDGEAGAQGAPGPAGPAGERGEQGPAGSPG

FQGLPGPAGPPGEAGKPGEQGAPGDLGAPGPSGARGERGFPGERGVQGPPGPAGPRGSNG

APGNDGAKGDAGAPGAPGSQGAPGLQGMPGERGAAGLPGPKGDRGDAGPKGADGAPGKDG

VRGLTGPIGPPGPAGAPGDKGETGPSGPAGPTGARGAPGDRGEPGPPGPAGFAGPPGADG

QPGAKGEPGDAGAKGDAGPAGPAGPAGPPGPIGNVGAPGPKGARGSAGPPGATGFPGAAG

RVGPPGPSGNAGPPGPPGPAGKEGGKGPRGETGPAGRPGEVGPPGPPGPAGEKGSPGSDG

PAGSPGTPGPQGIAGQRGVVGLPGQRGERGFPGLPGPSGEPGKQGPSGSSGERGPPGPMG

PPGLAGPPGESGREGSPGAEGSPGRDGSPGPKGDRGETGPAGPPGAPGAPGAPGPVGPAG

KSGDRGETGPAGPAGPIGPAGARGPAGPQGPRGDKGETGEQGDRGIKGHRGFSGLQGPPG

PPGSPGDQGPSGASGPAGPRGPPGSPGAAGKDGLNGLAGPIGPPGPRGRTGDAGPVGPPG

PPGPPGPPGPPSGGFDFSFMPQPPQEKAHDGGRYYRARQFDG-KGVGGGPGPMGLMGPRG

PPGAAGAPGPQGFQGPAGEPGEPGQTGPAGSRGPAGPPGKAGEDGHPGKPGRPGERGVVG

PQGARGFPGTPGLPGFKGIRGHNGLDGLKGQPGAPGIKGEPGAPGENGTPGQTGARGLPG

ERGRVGAPGPAGARGSDGSVGPVGPAGPIGSAGPPGFPGAPGPKGELGPVGNPGPSGPAG

PRGEVGLPGLSGPVGPPGNPGANGLAGAKGAAGLPGVAGAPGLPGPRGIPGPPGAAGAAG

PRGLIGEPGPAGSKGETGNKGEPGSAGAQGPPGPSGEEGKRGTAGEAGPAGPPGPAGLRG

NPGSRGLPGADGRAGVMGPAGPRGATGPAGARGPNGDAGRPGEPGLMGPRGFPGSPGNVG

PAGKEGPVGLPGIDGRPGPIGPAGARGEPGNIGFPGPKGPTGDAGKPGERGHAGLAGARG

APGPDGNNGAQGPPGPQGVQGGKGEQGPAGPPGFQGLPGPAGTAGEAGKPGERGLPGEFG

LPGPAGPRGERGPPGESGAVGPSGPIGSRGPSGPPGPDGNKGEPGSVGAPGSAGAPGPGG

LPGERGAAGIPGGKGDKGEPGLRGEMGTTGRDGARGAPGAMGAPGPSGASGDRGEAGAAG

PAGPAGPRGSPGERGEVGPAGPNGFAGPAGAAGQPGAKGERGTKGPKGENGVVGPTGPVG

AAGPSGPNGPPGPAGTRGDGGPPGMTGFPGAAGRTGPPGPSGITGPPGPPGASGKEGLRG

PRGDQGPVGRTGETGATGPPGFVGEKGPSGEPGAAGPPGTPGPQGLLGAPGILGLPGSRG

ERGLPGVSGSVGEPGPLGIAGPPGARGPPGAVGSPGVNGAPGEAGRDGNPGSDGPPGRDG

QPGHKGDRGYPGNAGPVGTVGAPGPHGPVGPTGKHGNRGEPGPAGSVGPTGAVGPRGPSG

AQGIRGDKGEPGEKGPRGLPGLKGHNGLQGLPGLAGHHGDQGAPGTVGPAGPRGPAGPSG

PPGKDGRNGHPGVVGPAGIRGTQGSQGPAGPPGPPGPPGPPGISGGGYDFGFDGDFYRA

>TURSIOPS

QMSYGYDEKSTG-ISVPGPMGPSGPRGLPGPPGTPGPQGFQGPPGEPGEPGASGPMGPRG

PPGPPGKNGDDGEAGKPGRPGERGPPGPQGARGLPGTAGLPGMKGHRGFSGLDGAKGDAG

PAGPKGEPGSPGENGAPGQMGPRGLPGERGRPGAPGPAGARGNDGATGAAGPPGSTGPAG

PPGFPGAVGAKGEAGPQGSRGSEGPQGVRGEPGPPGPAGATGPAGNPGADGQPGAKGANG

APGIAGAPGFPGARGPSGPQGPSGPPGPKGNSXXXXXXXXXXXXXXXXXXGPTGIQGPPG

PAGEEGKRGARGEPGPAGLPGPPGERGGPGSRGFPGADGIAGPKGPAGERGAPGLAGPKX

SPGEAGRPGEAGLPGAKGLTGSPGSPGPDGKTGPPGPAGQDGRPGPPGPPGSRGQAGVMG

FPGPKGAAGEPGKAGERGVPGPPGAAGPAGKDGEAGAQGPPGPAGPAGERGEQGPAGSPG

FQGLPGPSGPPGEAGKPGEQGAPGDLGAPGPSGARGERGFPGERGVQGPSGPAGPRGSNG

PPGNDGAKGDAGAPGAPGNQGAPGLQGMPGERGAAGLPGPKGDRGDAGPKGADGAPGKDG

VRGLTGPIGPPGPAGAPGDKGETGPSGPAGPTGARGAPGDRGEPGPPGPAGFAGPPGADG

QPGAKGEPGDAGAKGDAGAPGSAGPTGPPGPIGNVGAPGPKGARGSAGPPGATGFPGAAG

RVGPPGPSGNAGPPGPSGPAGKEGSKGPRGETGPAGRAGEVGPPGPPGPAGEKGAPGADG

PAGSPGSPGPQGIAGQRGVVGLPGQRGERGFPGLPGPSGEPGKQGPSGASGERGPPGPMG

PPGLAGPPGESGREGAPGAEGSPGRDGSPGPKGDRGETGPAGPPGAPGSPGAPGPVGPAG

KSGDRGETGPAGPAGPIGPAGARGPTGPQGPRGDKGETGEQGDRGIKGHRGFSGLQGPPG

PPGSPGEQGPSGASGPAGPRGPPGSAGTPGKDGLNGLPGPIGPPGPRGRTGDAGPAGPPG

PPGPPGPPGPPSGGYDFSFLPQPPQEKAQDGGRYYRARQYDG-KGVGLGPGPMGLMGPRG

PPGASGVPGPQGFQGPPGEPGEPGQTGPAGARGPPGPPGKAGEDGHPGKPGRPGERGVVG

PQGARGFPGTPGLPGFKGIRGHNGLDGLKGQPGTPGVKGEPGAPGENGIPGQVGARGLPG

ERGRVGAPGPAGARGSDGSVGPVGPAGPVGSAGPPGFPGAPGPKGELGPVGNPGPAGPAG

SRGEVGLPGVSGPVGPPGNPGANGLHGAKGAAGLPGVAGAPGLPGPRGIPGPVGAAGATG

ARGLVGEPGPAGSKGESGNKGEPGAAGPTGPPGPSGEEGKRGSTGEIGSAGPPGPPGLRG

NPGSRGLPGADGRAGVMGPHGSRGGTGPAGVRGPSGDSGRPGEPGLMGPRGFPGSPGNVG

PAGKEGPMGLPGIDGRPGPIGPAGARGEPGNIGFPGPKGPTGDPGKNGEKGHAGLAGPRG

APGPDGNNGAQGPPGPQGVSGGKGEQGPAGPPGFQGLPGPAGTAGEAGKAGERGLPGEFG

LPGPAGPRGERGPPGESGAAGPTGPVGSRGPSGPAGPDGNKGEPGVVGAPGSAGPSGPNG

LPGERGAAGIPGGKGEKGETGLRGDAGSHGRDGARGAPGAVGAPGPAGANGDRGEAGPAG

PAGPAGPRGSPGERGEVGPAGPNGFAGPAGAAGQPGAKGERGTKGPKGENGPTGPTGPVG

AAGPAGPNGPPGPAGSRGDGGPPGATGFPGAAGRTGPPGPSGITGPPGPPGPAGKEGLRG

PRGDQGPVGRTGETGASGPPGFVGEKGPSGEPGTAGSPGTPGPQGLLGAPGFLGLPGSRG

ERGLPGVAGSVGEPGPLGIAGPTGARGPPGAVGNPGVNGAPGEAGRDGNPGNDGPPGRDG

QAGHKGDRGYPGNAGPTGTVGAPGPQGPVGPTGKHGNRGEPGPSGPIGLAGAVGPRGPSG

PQGIRGDKGEPGDKGPRGLPGLKGHNGLQGLPGLAGHHGDQGAPGTVGPAGPRGPSGPSG

PSGKDGRTGHPGAVGPAGIRGSQGSQGPSGPPGPPGPPGPPGPSGGGYDFGFDGDFYRA

>BALAEONOPTERA

QMSYGYDEKSTG-ISVPGPMGPSGPRGLPGPPGAPGPQGFQGPPGEPGEPGASGPMGPRG

PPGPPGKNGDDGEAGKPGRPGERGPPGPQGARGLPGTAGLPGMKGHRGFSGLDGAKGDAG

PAGPKGEPGSPGENGAPGQMGPRGLPGERGRPGAPGPAGARGNDGATGAAGPPGPTGPAG

PPGFPGAVGAKGEAGPQGSRGSEGPQGVRGEPGPPGPAGATGPAGNPGADGQPGAKGANG

APGIAGAPGFPGARGPSGPQGPSGPPGPKGNSGEPGAPGNKGDTGAKGEPGPTGIQGPPG

PAGEEGKRGTRGEPGPAGLPGPPGERGGPGSRGFPGADGVSGPKGPAGERGAPGPAGPKG

SPGEAGRPGEAGLPGAKGLTGSPGSPGPDGKTGPPGPAGQDGRPGPPGPPGSRGQAGVMG

FPGPKGAAGEPGKAGERGVPGPPGAVGPAGKDGEAGAQGPPGPAGPAGERGEQGPAGSPG

FQGLPGPAGPPGEAGKPGEQGVPGDLGAPGPSGARGERGFPGERGVQGPSGPAGPRGSNG

APGNDGAKGDAGAPGAPGNQGAPGLQGMPGERGAAGLPGLKGDRGDVGPKGADGAPGKDG

VRGLTGPIGPPGPAGAPGDKGETGPSGPAGPTGARGAPGDRGEPGPPGPAGFAGPPGADG

QPGAKGEPGDAGAKGDAGLPGAAGPTGPPGPIGNVGAPGPKGARGSAGPPGATGFPGAAG

RVGPPGPSGNAGPPGPSGPAGKEGSKGARGETGPAGRAGEVGPPGPPGPAGEKGAPGADG

PAGAPGSPGPQGIAGQRGVVGLPGQRGERGFPGLPGPSGEPGKQGPSGASGERGPPGPMG

PPGLAGPPGESGREGAPGAEGSPGRDGSPGPKGDRGETGPAGPPGAPGAPGAPGPVGPAG

KSGDRGETGPAGPAGPIGPVGARGPAGPQGPRGDKGETGEQGDRGIKGHRGFSGLQGPPG

PPGSPGEQGPSGASGPAGPRGPPGSAGTPGKDGLNGLPGPIGPPGPRGRTGDAGPAGPPG

PPGPPGPPGPPSGGYDFSFMPQPPQEKAQDGGRYYRARQFDA-KGVG?GPGPMGLMGPRG

PPGASGAPGPQGFQGLPGEPGEPGQTGPAGSRGPPGPPGKAGEDGHPGKPGRPGERGVVG

PQGARGFPGTPGLPGFKGIRGHNGLDGLKGQPGAPGVKGEPGAPGENGTPGQTGARGLPG

ERGRVGAPGPAGARGSDGSVGPVGPAGPIGSAGPPGFPGAPGPKGELGPVGNPGPPGPAG

SRGEVGLAGVSGPVGPPGNPGANGLPGAKGAAGLPGVAGAPGLPGPRGIPGPVGAAGATG

ARGLVGEPGPAGSKGESGNKGEPGAAGPTGPPGPSGEEGKRGTTGEIGSAGPPGPPGLRG

NPGSRGLPGADGRAGVMGPHGSRGGTGPAGMRGPSGDSGRPGEPGLMGPRGFPGSPGNVG

PAGKEGPVGLPGIDGRPGAIGPAGARGEPGNIGFPGPKGPSGDPGKAGEKGHAGLAGARG

APGPEGNNGAQGPPGLQGVSGGKGEQGPAGPPGFQGLPGPAGTAGEAGKPGERGLPGEFG

LPGPAGARGERGPPGESGAAGPTGPIGNRGPSGPAGPDGNKGEPGVVGAPGTAGPSGPSG

LPGERGAAGIPGGKGEKGETGLRGDIGSPGRDGARGAPGAVGAPGPAGANGDRGEAGPAG

PAGPAGPRGSPGERGEVGPAGPNGFAGPAGAAGQPGAKGERGTKGPKGENGPAGPTGPVG

AAGPSGPNGPPGPAGSRGDGGPPGVTGFPGAAGRTGPPGPSGITGPPGPTGPAGKEGLRG

PRGDQGPVGRTGETGASGPPGFVGEKGPSGEPGTAGSPGTPGPQGLLGAPGFLGLPGSRG

ERGLPGVAGSVGEPGPLGISGPTGARGPPGAVGNPGVNGAPGEAGRDGNPGNDGPPGRDG

QPGHKGDRGYPGNAGPTGTAGAPGPQGPQGPVGKHGNRGEPGPAGAVGPAGAVGPRGPSG

PQGIRGDKGEPGDKGPRGLPGLKGHNGLQGLPGLKGHHGDQGAPGTVGPAGPRGPAGPSG

PSGKDGRTGHPGAVGPAGIRGSQGSQGPAGPPGPPGPPGPPGPSGGGYEFGFDGDFYRA

>BOS

QLSYGYDEKSTG-ISVPGPMGPSGPRGLPGPPGAPGPQGFQGPPGEPGEPGASGPMGPRG

PPGPPGKNGDDGEAGKPGRPGERGPPGPQGARGLPGTAGLPGMKGHRGFSGLDGAKGDAG

PAGPKGEPGSPGENGAPGQMGPRGLPGERGRPGAPGPAGARGNDGATGAAGPPGPTGPAG

PPGFPGAVGAKGEGGPQGPRGSEGPQGVRGEPGPPGPAGAAGPAGNPGADGQPGAKGANG

APGIAGAPGFPGARGPSGPQGPSGPPGPKGNSGEPGAPGSKGDTGAKGEPGPTGIQGPPG

PAGEEGKRGARGEPGPAGLPGPPGERGGPGSRGFPGADGVAGPKGPAGERGAPGPAGPKG

SPGEAGRPGEAGLPGAKGLTGSPGSPGPDGKTGPPGPAGQDGRPGPPGPPGARGQAGVMG

FPGPKGAAGEPGKAGERGVPGPPGAVGPAGKDGEAGAQGPPGPAGPAGERGEQGPAGSPG

FQGLPGPAGPPGEAGKPGEQGVPGDLGAPGPSGARGERGFPGERGVQGPPGPAGPRGANG

APGNDGAKGDAGAPGAPGSQGAPGLQGMPGERGAAGLPGPKGDRGDAGPKGADGAPGKDG

VRGLTGPIGPPGPAGAPGDKGEAGPSGPAGPTGARGAPGDRGEPGPPGPAGFAGPPGADG

QPGAKGEPGDAGAKGDAGPPGPAGPAGPPGPIGNVGAPGPKGARGSAGPPGATGFPGAAG

RVGPPGPSGNAGPPGPPGPAGKEGSKGPRGETGPAGRPGEVGPPGPPGPAGEKGAPGADG

PAGAPGTPGPQGIAGQRGVVGLPGQRGERGFPGLPGPSGEPGKQGPSGASGERGPPGPMG

PPGLAGPPGESGREGAPGAEGSPGRDGSPGAKGDRGETGPAGPPGAPGAPGAPGPVGPAG

KSGDRGETGPAGPAGPIGPVGARGPAGPQGPRGDKGETGEQGDRGIKGHRGFSGLQGPPG

PPGSPGEQGPSGASGPAGPRGPPGSAGSPGKDGLNGLPGPIGPPGPRGRTGDAGPAGPPG

PPGPPGPPGPPSGGYDLSFLPQPPQEKAHDGGRYYRARQFDA-KG-G-GPGPMGLMGPRG

PPGASGAPGPQGFQGPPGEPGEPGQTGPAGARGPPGPPGKAGEDGHPGKPGRPGERGVVG

PQGARGFPGTPGLPGFKGIRGHNGLDGLKGQPGAPGVKGEPGAPGENGTPGQTGARGLPG

ERGRVGAPGPAGARGSDGSVGPVGPAGPIGSAGPPGFPGAPGPKGELGPVGNPGPAGPAG

PRGEVGLPGLSGPVGPPGNPGANGLPGAKGAAGLPGVAGAPGLPGPRGIPGPVGAAGATG

ARGLVGEPGPAGSKGESGNKGEPGAVGQPGPPGPSGEEGKRGSTGEIGPAGPPGPPGLRG

NPGSRGLPGADGRAGVMGPAGSRGATGPAGVRGPNGDSGRPGEPGLMGPRGFPGSPGNIG

PAGKEGPVGLPGIDGRPGPIGPAGARGEPGNIGFPGPKGPSGDPGKAGEKGHAGLAGARG

APGPDGNNGAQGPPGLQGVQGGKGEQGPAGPPGFQGLPGPAGTAGEAGKPGERGLPGEFG

LPGPAGARGERGPPGESGAAGPTGPIGSRGPSGPPGPDGNKGEPGVVGAPGTAGPSGPSG

LPGERGAAGIPGGKGEKGETGLRGDIGSPGRDGARGAPGAIGAPGPAGANGDRGEAGPAG

PAGPAGPRGSPGERGEVGPAGPNGFAGPAGAAGQPGAKGERGTKGPKGENGPVGPTGPVG

AAGPSGPNGPPGPAGSRGDGGPPGATGFPGAAGRTGPPGPSGISGPPGPPGPAGKEGLRG

PRGDQGPVGRSGETGASGPPGFVGEKGPSGEPGTAGPPGTPGPQGLLGAPGFLGLPGSRG

ERGLPGVAGSVGEPGPLGIAGPPGARGPPGNVGNPGVNGAPGEAGRDGNPGNDGPPGRDG

QPGHKGERGYPGNAGPVGAAGAPGPQGPVGPVGKHGNRGEPGPAGAVGPAGAVGPRGPSG

PQGIRGDKGEPGDKGPRGLPGLKGHNGLQGLPGLAGHHGDQGAPGAVGPAGPRGPAGPSG

PAGKDGRIGQPGAVGPAGIRGSQGSQGPAGPPGPPGPPGPPGPSGGGYEFGFDGDFYRA

>OVIS

QLSYGYDEKSTG-ISVPGPMGPSGPRGLPGPPGAPGPQGFQGPPGEPGEPGASGPMGPRG

PPGPPGKNGDDGEAGKPGRPGERGPPGPQGARGLPGTAGLPGMKGHRGFSGLDGAKGDAG

PAGPKGEPGSPGENGTPGQMGPRGLPGERGRPGAPGPAGARGNDGATGAAGPPGPTGPAG

PPGFPGAVGAKGEAGPQGPRGSEGPQGVRGEPGPPGPAGAAGPAGNPGADGQPGAKGANG

APGIAGAPGFPGARGPSGPQGPSGPPGPKGNSGEPGAPGSKGDTGAKGEPGPTGIQGPPG

PAGEEGKRGARGEPGPAGLPGPPGERGGPGSRGFPGADGVAGPKGPAGERGAPGPAGPKG

SPGEAGRPGEAGLPGAKGLTGSPGSPGPDGKTGPPGPAGQDGRPGPPGPPGARGQAGVMG

FPGPKGAAGEPGKAGERGVPGPPGAVGPAGKDGEAGAQGPPGPAGPAGERGEQGPAGSPG

FQGLPGPAGPPGEAGKPGEQGVPGDLGAPGPSGARGERGFPGERGVQGPPGPAGPRGANG

APGNDGAKGDAGAPGAPGSQGAPGLQGMPGERGAAGLPGPKGDRGDAGPKGADGAPGKDG

VRGLTGPIGPPGPAGAPGDKGETGPSGPAGPTGARGAPGDRGEPGPPGPAGFAGPPGADG

QPGAKGEPGDAGAKGDAGPPGPAGPAGPPGPIGNVGAPGPKGARGSAGPPGATGFPGAAG

RVGPPGPSGNAGPPGPPGPAGKEGSKGPRGETGPAGRAGEVGPPGPPGPAGEKGAPGADG

PAGAPGTPGPQGIAGQRGVVGLPGQRGERGFPGLPGPSGEPGKQGPSGASGERGPPGPMG

PPGLAGPPGESGREGAPGAEGSPGRDGAPGAKGDRGETGPAGPPGAPGAPGAPGPVGPAG

KSGDRGETGPAGPAGPIGPVGARGPAGPQGPRGDKGETGEQGDRGIKGHRGFSGLQGPPG

PPGSPGEQGPSGASGPAGPRGPPGSAGTPGKDGLNGLPGPIGPPGPRGRTGDAGPAGPPG

PPGPPGPPGPPSGGYDLSFLPQPPQEKAHDGGRYYRARQFDG-KG-G-GPGPMGLMGPRG

PPGASGAPGPQGFQGPPGEPGEPGQTGPAGARGPPGPPGKAGEDGHPGKPGRPGERGVVG

PQGARGFPGTPGLPGFKGIRGHNGLDGLKGQPGAPGVKGEPGAPGENGTPGQTGARGLPG

ERGRVGAPGPAGARGSDGSVGPVGPAGPIGSAGPPGFPGAPGPKGELGPVGNPGPAGPAG

PRGEVGLPGLSGPVGPPGNPGANGLPGAKGAAGLPGVAGAPGLPGPRGIPGPVGAAGATG

ARGLVGEPGPAGSKGESGNKGEPGAVGQPGPPGPSGEEGKRGSTGEIGPAGPPGPPGLRG

NPGSRGLPGADGRAGVMGPAGSRGATGPAGVRGPNGDSGRPGEPGLMGPRGFPGSPGNIG

PAGKEGPAGLPGIDGRPGPIGPAGARGEPGNIGFPGPKGPTGDPGKAGEKGHAGLAGPRG

APGPDGNNGAQGPPGLQGVQGGKGEQGPAGPPGFQGLPGPAGTAGEAGKPGERGLPGEFG

LPGPAGARGERGPPGESGAAGPTGPIGSRGPSGPPGPDGNKGEPGVVGAPGTAGPSGPSG

LPGERGAAGIPGGKGEKGETGLRGDVGSPGRDGARGAPGAVGAPGPAGANGDRGEAGPAG

PAGPAGPRGSPGERGEVGPAGPNGFAGPAGAAGQPGAKGERGTKGPKGENGPVGPTGPVG

AAGPSGPNGPPGPAGSRGDGGPPGATGFPGAAGRTGPPGPAGISGPPGPPGPAGKEGLRG

PRGDQGPVGRTGEPGAAGPPGFVGEKGPSGEPGTAGPPGTPGPQGLLGAPGFLGLPGSRG

ERGLPGVAGSVGEPGPLGIAGPPGARGPPGNVGNPGVNGAPGEAGRDGNPGNDGPPGRDG

QPGHKGERGYPGNAGPVGAAGAPGPQGPVGPTGKHGSRGEPGPVGAVGPAGAVGPRGPSG

PQGIRGDKGEPGDKGPRGLPGLKGHNGLQGLPGLAGHHGDQGAPGAVGPAGPRGPAGPTG

PAGKDGRTGQPGAVGPAGIRGSQGSQGPAGPPGPPGPPGPPGPSGGGYDFGFDGDFYRA

>SUS

QLSYGYDEKSAG-ISVPGPMGPSGPRGLPGPPGAPGPQGFQGPPGEPGEPGASGPMGPRG

PPGPPGKNGDDGEAGKPGRPGERGPPGPQGARGLPGPAGLPGMKGHRGFSGLDGAKGDAG

PAGPKGEPGSPGENGAPGQMGPRGLPGERGRPGPPGTAGARGNDGATGAAGPPGPTGPAG

PPGFPGAVGAKGEAGPQGARGSEGPQGVRGEPGPPGPAGAAGPAGNPGADGQPGAKGANG

APGIAGAPGFPGARGPSGPQGPSGPPGPKGNSGEPGAPGSKGDTGAKGEPGPTGVQGPPG

PAGEEGKRGARGEPGPAGLPGPPGERGGPGSRGFPGADGVAGPKGPAGERGSPGPAGPKG

SPGEAGRPGEAGLPGAKGLTGSPGSPGPDGKTGPPGPAGQDGRPGPPGPPGARGQAGVMG

FPGPKGAAGEPGKAGERGVPGPPGAVGPAGKDGEAGAQGPPGPAGPAGERGEQGPAGSPG

FQGLPGPAGPPGEAGKPGEQGVPGDLGAPGPSGARGERGFPGERGVQGPPGPAGPRGANG

APGNDGAKGDAGAPGAPGSQGAPGLQGMPGERGAAGLPGPKGDRGDAGPKGADGAPGKDG

VRGLTGPIGPPGPAGAPGDKGETGPSGPAGPTGARGAPGDRGEPGPPGPAGFAGPPGADG

QPGAKGEPGDAGAKGDAGPPGPAGPTGPPGPIGSVGAPGPKGARGSAGPPGATGFPGAAG

RVGPPGPSGNAGPPGPPGPAGKEGSKGPRGETGPAGRPGEVGPPGPPGPAGEKGSPGADG

PAGAPGTPGPQGIAGQRGVVGLPGQRGERGFPGLPGPSGEPGKQGPSGPSGERGPPGPMG

PPGLAGPPGESGREGAPGAEGSPGRDGAPGPKGDRGESGPAGPPGAPGAPGAPGPVGPAG

KSGDRGETGPAGPAGPVGPVGARGPAGPQGPRGDKGETGEQGDRGIKGHRGFSGLQGPPG

PPGSPGEQGPSGASGPAGPRGPPGSAGAPGKDGLNGLPGPIGPPGPRGRTGDAGPVGPPG

PPGPPGPPGPPSGGFDFSFLPQPPQEKAHDGGRYYRARQYDG-KGVGAGPGPMGLMGPRG

PPGAVGAPGPQGFQGPAGEPGEPGQTGPAGARGPPGPPGKAGEDGHPGKPGRPGERGVVG

PQGARGFPGTPGLPGFKGIRGHNGLDGLKGQPGAPGVKGEPGAPGENGTPGQTGARGLPG

ERGRVGAPGPAGARGNDGSVGPVGPAGPIGSAGPPGFPGAPGPKGELGPVGNPGPAGPAG

PRGEVGLPGVSGPVGPPGNPGANGLPGAKGAAGLPGVAGAPGLPGPRGIPGPAGAAGATG

ARGLVGEPGPAGSKGESGNKGEPGAAGPQGPPGPSGEEGKRGPNGEVGSAGPPGPPGLRG

NPGSRGLPGADGRAGVMGPPGSRGPTGPAGVRGPNGDSGRPGEPGLMGPRGFPGSPGNVG

PAGKEGPAGLPGIDGRPGPIGPAGARGEPGNIGFPGPKGPTGDPGKNGEKGHAGLAGARG

APGPDGNNGAQGPPGPQGVQGGKGEQGPAGPPGFQGLPGPAGTAGEVGKPGERGLPGEFG

LPGPAGPRGERGPPGESGAAGPAGPIGSRGPSGPPGPDGNKGEPGVLGAPGTAGPSGPSG

LPGERGAAGIPGGKGEKGETGLRGDVGSPGRDGARGAPGAVGAPGPAGANGDRGEAGPAG

PAGPAGPRGSPGERGEVGPAGPNGFAGPAGAAGQPGAKGERGTKGPKGENGPVGPTGPVG

AAGPAGPNGPPGPAGSRGDGGPPGATGFPGAAGRIGPPGPSGISGPPGPPGPAGKEGLRG

PRGDQGPVGRTGETGASGPPGFAGEKGPSGEPGTAGPPGTPGPQGLLGAPGFLGLPGSRG

ERGLPGVAGSVGEPGPLGIAGPPGARGPPGAVGNPGVNGAPGEAGRDGNPGSDGPPGRDG

QAGHKGERGYPGNPGPAGAAGAPGPQGAVGPAGKHGNRGEPGPAGSVGPAGAVGPRGPSG

PQGIRGDKGEPGDKGPRGLPGLKGHNGLQGLPGLAGHHGDQGAPGPVGPAGPRGPAGPSG

PAGKDGRTGQPGAVGPAGIRGSQGSQGPAGPPGPPGPPGPPGPSGGGYDFGYEGDFYRA

>CANIS

QMSYGYDEKSTGGISVPGPMGPSGPRGLPGPPGAPGPQGFQGPPGEPGEPGASGPMGPRG

PPGPPGKNGDDGEAGKPGRPGERGPPGPQGARGLPGTAGLPGMKGHRGFSGLDGAKGDAG

PAGPKGEPGSPGENGAPGQMGPRGLPGERGRPGAPGPAGARGNDGATGAAGPPGPTGPAG

PPGFPGAVGAKGEAGPQGARGSEGPQGVRGEPGPPGPAGAAGPAGNPGADGQPGAKGANG

APGIAGAPGFPGARGPSGPQGPSGPPGPKGNSGEPGAPGNKGDTGAKGEPGPTGIQGPPG

PAGEEGKRGARGEPGPTGLPGPPGERGGPGSRGFPGADGVAGPKGPAGERGSPGPAGPKG

SPGEAGRPGEAGLPGAKGLTGSPGSPGPDGKTGPPGPAGQDGRPGPPGPPGARGQAGVMG

FPGPKGAAGEPGKAGERGVPGPPGAVGPAGKDGEAGAQGPPGPAGPAGERGEQGPAGSPG

FQGLPGPAGPPGEAGKPGEQGVPGDLGAPGPSGARGERGFPGERGVQGPPGPAGPRGANG

APGNDGAKGDAGAPGAPGSQGAPGLQGMPGERGAAGLPGPKGDRGDAGPKGADGSPGKDG

VRGLTGPIGPPGPAGAPGDKGEAGPSGPAGPTGARGAPGDRGEPGPPGPAGFAGPPGADG

QPGAKGEPGDAGAKGDAGPPGPAGPTGPPGPIGNVGAPGPKGARGSAGPPGATGFPGAAG

RVGPPGPSGNAGPPGPPGPAGKEGGKGARGETGPAGRPGEVGPPGPPGPAGEKGSPGADG

PAGAPGTPGPQGIAGQRGVVGLPGQRGERGFPGLPGPSGEPGKQGPSGTSGERGPPGPMG

PPGLAGPPGESGREGAPGAEGSPGRDGSPGPKGDRGETGPAGPPGAPGAPGAPGPVGPAG

KNGDRGETGPAGPAGPIGPVGARGPAGPQGPRGDKGETGEQGDRGIKGHRGFSGLQGPPG

PPGSPGEQGPSGASGPAGPRGPPGSAGSPGKDGLNGLPGPIGPPGPRGRTGDAGPVGPPG

PPGPPGPPGPPSGGFDFSFLPQPPQEKAHDGGRYYRARQYDG-KGVGLGPGPMGLMGPRG

PPGASGAPGPQGFQGPAGEPGEPGQTGPAGARGPPGPPGKAGEDGHPGKPGRPGERGVVG

PQGARGFPGTPGLPGFKGIRGHNGLDGLKGQPGAPGVKGEPGAPGENGTPGQTGARGLPG

ERGRVGAPGPAGARGSDGSVGPVGPAGPIGSAGPPGFPGAPGPKGELGPVGNPGPAGPAG

PRGEVGLPGVSGPVGPPGNPGANGLTGAKGAAGLPGVAGAPGLPGPRGIPGPVGAAGATG

ARGLVGEPGPAGSKGESGNKGEPGSAGAQGPPGPSGEEGKRGPNGEAGSAGPSGPPGLRG

SPGSRGLPGADGPAGVMGPPGPRGATGPAGVRGPNGDSGRPGEPGLMGPRGFPGAPGNVG

PAGKEGPMGLPGIDGRPGPIGPAGARGEPGNIGFPGPKGPTGDPGKNGDKGHAGLAGARG

APGPDGNNGAQGPPGPQGVQGGKGEQGPAGPPGFQGLPGPAGTAGEVGKPGERGLPGEFG

LPGPAGPRGERGPPGESGAAGPSGPIGSRGPSGPPGPDGNKGEPGVLGAPGTAGASGPGG

LPGERGAAGIPGGKGEKGETGLRGEIGNPGRDGARGAPGAMGAPGPAGATGDRGEAGPAG

PAGPAGPRGTPGERGEVGPAGPNGFAGPAGAAGQPGAKGERGTKGPKGENGPVGPTGPIG

SAGPSGPNGPPGPAGSRGDGGPPGATGFPGAAGRTGPPGPSGITGPPGPPGAAGKEGLRG

PRGDQGPVGRTGETGASGPPGFTGEKGPSGEPGTAGPPGTPGPQGLLGAPGILGLPGSRG

ERGLPGVAGSVGEPGPLGIAGPPGARGPPGAVGAPGVNGAPGEAGRDGNPGNDGPPGRDG

QAGHKGERGYPGNIGPVGAVGAPGPHGPVGPTGKHGNRGEPGPAGSVGPVGAVGPRGPSG

PQGIRGDKGEPGEKGPRGLPGLKGHNGLQGLPGLAGQHGDQGAPGSVGPAGPRGPAGPSG

PAGKDGRTGQPGTVGPAGIRGSQGSQGPAGPPGPPGPPGPPGPSGGGYDFGYEGDFYRA

>AILUROPODA

QMSYGYDEKSTGGISVPGPMGPSGPRGLPGPPGAPGPQGFQGPPGEPGEPGASGPMGPRG

PPGPPGKNGDDGEAGKPGRPGERGPPGPQGARGLPGTAGLPGMKGHRGFSGLDGAKGDAG

PAGPKGEPGSPGENGAPGQMGPRGLPGERGRPGAPGPAGARGNDGATGAAGPPGPTGPAG

PPGFPGAVGAKGEAGPQGARGSEGPQGVRGEPGPPGPAGAAGPAGNPGADGQPGAKGANG

APGIAGAPGFPGARGPSGPQGPSGPPGPKGNSGEPGAPGNKGDTGAKGEPGPTGIQGPPG

PAGEEGKRGARGEPGPTGLPGPPGERGGPGSRGFPGADGVAGPKGPAGERGSPGPAGPKG

SPGEAGRPGEAGLPGAKGLTGSPGSPGPDGKTGPPGPAGQDGRPGPPGPPGARGQAGVMG

FPGPKGAAGEPGKAGERGVPGPPGAVGPAGKDGEAGAQGPPGPAGPAGERGEQGPAGSPG

FQGLPGPAGPPGEAGKPGEQGVPGDLGAPGPSGARGERGFPGERGVQGPPGPAGPRGANG

APGNDGAKGDAGAPGAPGSQGAPGLQGMPGERGAAGLPGPKGDRGDAGPKGADGSPGKDG

VRGLTGPIGPPGPAGAPGDKGEAGPSGPAGPTGARGAPGDRGEPGPPGPAGFAGPPGADG

QPGAKGEPGDAGAKGDAGPPGPAGPTGPPGPIGNVGAPGPKGARGSAGPPGATGFPGAAG

RVGPPGPSGNAGPPGPPGPAGKEGGKGPRGETGPAGRPGEVGPPGPPGPAGEKGSPGADG

PAGAPGTPGPQGIAGQRGVVGLPGQRGERGFPGLPGPSGEPGKQGPSGASGERGPPGPMG

PPGLAGPPGESGREGSPGAEGSPGRDGSPGPKGDRGETGPAGPPGAPGAPGAPGPVGPAG

KSGDRGETGPAGPAGPIGPVGARGPAGPQGPRGDKGETGEQGDRGIKGHRGFSGLQGPPG

PPGSPGEQGPSGASGPAGPRGPPGSAGSPGKDGLNGLPGPIGPPGPRGRTGDAGPVGPPG

PPGPPGPPGPPSGGFDFSFLPQPPQEKAHDGGRYYRARQYDG-KGVGLGPGPMGLMGPRG

PPGASGAPGPQGFQGPAGEPGEPGQTGPAGARGPPGPPGKAGEDGHPGKPGRPGERGVVG

PQGARGFPGTPGLPGFKGIRGHNGLDGLKGQPGAPGVKGEPGAPGENGTPGQTGARGLPG

ERGRVGAPGPAGARGSDGSVGPVGPAGPIGSAGPPGFPGAPGPKGELGPVGNPGPAGPAG

PRGEVGLPGVSGPVGPPGNPGANGLTGAKGAAGLPGVAGAPGLPGPRGIPGPVGAAGATG

ARGLVGEPGPAGSKGESGNKGEPGSVGPQGPPGPSGEEGKRGPNGEAGSAGPSGPPGLRG

SPGSRGLPGADGRAGVMGPPGPRGSTGPAGVRGPNGDSGRPGEPGLMGPRGFPGAPGNVG

PAGKEGPMGLPGIDGRPGPIGPAGARGEPGNIGFPGPKGPSGEPGKAGEKGHAGLAGARG

APGPDGNNGAQGPPGPQGVQGGKGEQGPAGPPGFQGLPGPAGTAGEVGKPGERGLPGEFG

LPGPAGPRGERGPPGESGAAGPSGPIGSRGPSGPPGPDGNKGEPGVLGAPGTAGPSGPGG

LPGERGAAGVPGGKGEKGETGLRGEVGNPGRDGARGAPGAVGAPGPAGATGDRGEAGPAG

PAGPAGPRGSPGERGEVGPAGPNGFAGPAGAAGQPGAKGERGTKGPKGENGPVGPTGPVG

SAGPSGPNGPPGPAGSRGDGGPPGATGFPGAAGRTGPPGPSGITGPPGPPGAAGKEGLRG

PRGDQGPVGRTGETGAHGPPGFAGEKGPSGEPGTAGPPGTAGPQGLLGAPGILGLPGSRG

ERGLPGVSGSVGEPGPLGIAGPPGARGPPGAVGAPGVNGAPGEAGRDGNPGNDGPPGRDG

QPGHKGERGYPGNIGPVGTVGAPGPHGPVGPTGKHGNRGEPGPAGAVGPVGAVGPRGPSG

PQGVRGDKGEPGDKGPRGLPGLKGHNGLQGLPGLAGQHGDQGAPGSVGPAGPRGPAGPSG

PAGKDGRTGHPGTVGPAGVRGSQGSQGPAGPPGPPGPPGPPGPSGGGYDFGYEGDFYRA

>FELIS

QMSYGYDEKSTGGISVPGPMGPSGPRGLPGPPGAPGPQGFQGPPGEPGEPGASGPMGPRG

PPGPPGKNGDDGEAGKPGRPGERGPPGPQGARGLPGTAGLPGMKGHRGFSGLDGAKGDAG

PAGPKGEPGSPGENGAPGQMGPRGLPGERGRPGAPGPAGARGNDGATGAAGPPGPTGPAG

PPGFPGAVGAKGEAGPQGARGSEGPQGVRGEPGPPGPAGAAGPAGNPGADGQPGAKGANG

APGIAGAPGFPGARGPSGPQGPSGPPGPKGNSGEPGAPGNKGDTGAKGEPGPTGIQGPPG

PAGEEGKRGARGEPGPTGLPGPPGERGGPGSRGFPGADGVAGPKGPAGERGSPGPAGPKG

SPGEAGRPGEAGLPGAKGLTGSPGSPGPDGKTGPPGPAGQDGRPGPPGPPGARGQAGVMG

FPGPKGAAGEPGKAGERGVPGPPGAVGPAGKDGEAGAQGPPGPAGPAGERGEQGPAGSPG

FQGLPGPAGPPGEAGKPGEQGVPGDLGAPGPSGARGERGFPGERGVQGPPGPAGPRGANG

APGNDGAKGDAGAPGAPGSQGAPGLQGMPGERGAAGLPGPKGDRGDAGPKGADGSPGKDG

VRGLTGPIGPPGPAGAPGDKGEAGPSGPAGPTGARGAPGDRGEPGPPGPAGFAGPPGADG

QPGAKGEPGDAGAKGDAGPPGPAGPTGPPGPIGNVGAPGPKGARGSAGPPGATGFPGAAG

RVGPPGPSGNAGPPGPPGPVGKEGGKGPRGETGPAGRPGEVGPPGPPGPAGEKGSPGADG

PAGAPGTPGPQGIAGQRGVVGLPGQRGERGFPGLPGPSGEPGKQGPSGPSGERGPPGPMG

PPGLAGPPGESGREGSPGAEGSPGRDGSPGPKGDRGETGPAGPPGAPGAPGAPGPVGPAG

KSGDRGETGPAGPAGPIGPVGARGPAGPQGPRGDKGETGEQGDRGIKGHRGFSGLQGPPG

PPGSPGEQGPSGASGPAGPRGPPGAAGSPGKDGLNGLPGPIGPPGPRGRTGDAGPVGPPG

PPGPPGPPGPPSGGFDFSFLPQPPQEKAHDGGRYYRARQYDPGKGVGLGPGPMGLMGPRG

PPGASGAPGPQGFQGPAGEPGEPGQTGPAGARGPPGPPGKAGEDGHPGKPGRPGERGVVG

PQGARGFPGTPGLPGFKGIRGHNGLDGLKGQPGAPGVKGEPGAPGENGTPGQTGARGLPG

ERGRVGAPGPAGARGSDGSVGPVGPAGPIGSAGPPGFPGAPGPKGELGPVGNPGPAGPAG

PRGEMGLPGVSGPVGPPGNPGANGLTGAKGAAGLPGVAGAPGLPGPRGIPGPVGAAGATG

ARGLVGEPGPAGSKGESGNKGEPGSAGPQGPPGPSGEEGKRGPNGEAGSAGPSGPPGLRG

SPGSRGLPGADGRAGVMGPPGPRGATGPAGVRGPNGDAGRPGEPGLMGPRGFPGAPGNVG

PAGKEGPMGLPGIDGRPGPIGPAGARGEPGNIGFPGPKGPTGDPGKNGDKGHAGLAGARG

APGPDGNNGAQGPPGPQGVQGGKGEQGPAGPPGFQGLPGPAGTAGEVGKPGERGLPGEFG

LPGPAGPRGERGPPGESGAAGPSGPIGSRGPSGPPGPDGNKGEPGVLGAPGTAGPSGPSG

LPGERGAAGIPGGKGEKGETGLRGEIGNPGRDGARGAPGAVGAPGPAGATGDRGEAGPAG

PAGPAGPRGSPGERGEVGPAGPNGFAGPAGAAGQPGAKGERGTKGPKGENGPVGPTGPVG

SAGPSGPNGPPGPAGSRGDGGPPGATGFPGAAGRTGPPGPSGITGPPGPPGAAGKEGLRG

PRGDQGPVGRTGETGASGPPGFAGEKGPSGEPGTAGPPGTPGPQGLLGAPGILGLPGSRG

ERGLPGVSGSVGEPGPLGISGPPGARGPSGAVGAPGVNGAPGEAGRDGNPGNDGPPGRDG

QPGHKGERGYPGNIGPVGAVGAPGPHGPVGPTGKHGNRGEPGPAGVVGPVGAVGPRGPTG

PQGIRGDKGEPGDKGPRGLPGLKGHNGLQGLPGLAGQHGDQGAPGSVGPAGPRGPAGPSG

PMGKDGRTGHPGSVGPAGVRGSQGSQGPAGPPGPPGPPGPPGPSGGGYDFGYEGDFYRA

>ORNITHORHYNCHUS

QMAYGYDEKAGGGMSVPGPMGPSGPRGLPGPPGSPXXXXXXXXXXXXXXXXXXGPMGPRG

PAGPPGKNGDDGEAGKPGRPGERGPPGPQGARGLPGTAGLPGMKGHRGFSGLDGAKGDSG

PAGPKGEPGSAGENGAPGQMGPRGLPGERGRPGPSGPAGARGNDGAPGAAGPPGPTGPAG

PPGFPGAVGAKGEAGAQGSRGSEGPQGARGEPGPPGPAGAAGPSGNPGSDGQPGAKGANG

APGIAGAPGFPGARGPSGPQGPSGGPGPKGNSGEPGAPGNKGDPGAKGEPGPVGVQGPPG

PSGEEGKRGSRGEPGPTGLPGPAGERGGPGSRGFPGADGVAGPKGPAGERGSPGPAGPKG

SPGEAGRPGEAGLPGAKXXXXXXXXXXXXXXXXXXXXXXXXXXXXXXXXXXXXXXXXXXX

XXXXXXXXGEPGKPGERGVPGPPGAVGAAGKDGEAGAQGPPGPAGPAGERGEQGPSGSPG

FQGLPGPSGPAGESGKPGEQGVPGDAGAPGPSGARGERGFPGERGVQGPAGPQGPRGSNG

APGNDGAKGDAGAPGAPGGQGPPGLQGMPGERGAAGLPGAKGDRGDAGPKGGDGAPGKDG

IRGLTGPIGPPGPAGNPGDKGESGPSGPAGPTGARGAPGDRGEPGPPGPAGFAGPPGADG

QPGAKGETGDSGAKGDAGPPGPAGPTGAPGPAGNVGAPGPKGARGSAGPPGATGFPGAAG

RVGPPGPSXXXXXXXXXXXXXXXXXXXXXXXXXXXXXXXXXXXXXXXXXXXXXXXXXXXX

XXXXXXXXXXXXXXXXXXXXXXXXXXXXXXXXXXXXXXXXXXXXXXXXXXXXXXXXXXXX

XXXXXXXXXXXXXXXXXXXXXXXXXXXXXXXXGDRGETGPAGPPGAPGAPGAPGPVGPAG

KNGDRGETGPSGPAGPAGPAGARGPSGPQGPRGDKGETGEQGDRGMKGHRGFSGLQGPPG

PPGSPGEQGPSGASGPAGPRXXXXXXXXXXXXXXXXXXXXXXXXXXXXXXXXXXXXXXXX

XXXXXXXXXXXXXXXXXXXXXXXXXXXXXXXXXXXXXXQYDGSKAADMGPGPMGLMGPRG

PPGASGAPGAQGFQGPPGEPGEPGQSGPAGSRGPAGPPGKSGEDGHPGKPGRSGERGVVG

PQGARGFPGTPGLPGFKGIRGHNGLDGQKGQPGTPGVKGEPGAPGENGSPGQSGARGLPG

ERGRIGGAGPTGARGSDGSVGPVGPAGPIGSAGPPGFPGAPGPKGELGAVGNTGPAGPAG

PRGELGLPGVSGPVGPAGNPGANGLAGAKGAAGLPGVAGAPGLPGPRGIPGPSGPSGPSG

PRGLVGEPGPAGSKGESGSKGEPGSAGAQGPPGPNGEEGKRGPNGEPGSTGPTGPPGLRG

VPGSRGLPGADGRAGGMGPAGNRGSAGPSGARGPSGDSGRPGEPGLVGPRGLPGFPGNVG

PAGKEGPVGLPGSEGRPGPTGPAGARGEPGNIGFPGPKGPNGEPGKSGERGHAGLAGSRG

APGPDGNNGAXXXXXXXXXXXXXXXXXXXXXXXXXXXXXXXXXXXXXXXXXXXGLPGEFG

LPGPAGPRGERGPPGESGAAGPTGPIGNRGPSGPPGPDGNKGEPGVAGAPGNAGPAGSGG

LPGERGAVGVPGGKGEKGEPGLRGEFGNPGRDGARGAPGAVGSPGPSGATGDRGEAGAAG

PAGPAGPRGSPGERGEVGPAGPNGFAGPPGAAGQAGAKGERGTKGPKGENGPTGPVGAVG

SAGPAGPNGLPGPTGGRGDGGPPGMTGFPGAAGRTGPAGPSGITGPSGPPGASGKEGPRG

PRGDQGPVGRTGELGAVGPPGFTGEKGPSGEPGTAGPPGTPGPQGLLGSPGILGLPGSRG

ERGLPGVSGGLGEPGPLGISGPSGARGPPGNVGNPGVNGAPGEAGRDXXXXXXXXXXXXX

XXXXXXXXXXXXXXXXXXXXXXXXXXXXXXXXXXXXXXXXXXXXXXXXXXXXXXXXXXXX

XXXXXXXXXXXXXXXXXXXXXXXXXXXXXXXXXXXXXXXXXXXXXXXXXXXXXXXXXXXX

XXXXXXXXXXXXXXXXXXXXXXXXXXXXXXXXXXXXXXXXXXXXXXXXXXXXXXXXXXX

>PONGO

QLSYGYDEKSTGGISVPGPMGPSGPRGLPGPPGAPGPQGFQGPPGEPGEPGASGPMGPRG

PPGPPGKNGDDGEAGKPGRPGERGPPGPQGARGLPGTAGLPGMKGHRGFSGLDGAKGDAG

PAGPKGEPGSPGENGAPGQMGPRGLPGERGRPGAPGPAGARGNDGATGAAGPPGPTGPAG

PPGFPGAVGAKGEAGPQGPRGSEGPQGVRGEPGPPGPAGAAGPAGNPGADGQPGAKGANG

APGIAGAPGFPGARGPSGPQGPGGPPGPKGNSGEPGAPGSKGDTGAKGEPGPVGVQGPPG

PAGEEGKRGARGEPGPTGLPGPPGERGGPGSRGFPGADGVAGPKGPAGERGSPGPAGPKG

SPGEAGRPGEAGLPGAKGLTGSPGSPGPDGKTGPPGPAGQDGRPGPPGPPGARGQAGVMG

FPGPKGAAGEPGKAGERGVPGPPGAVGPAGKDGEAGAQGPPGPAGPAGERGEQGPAGSPG

FQGLPGPAGPPGEAGKPGEQGVPGDLGAPGPSGARGERGFPGERGVQGPPGPAGPRGANG

APGNDGAKGDAGAPGAPGSQGAPGLQGMPGERGAAGLPGPKGDRGDAGPKGADGSPGKDG

VRGLTGPIGPPGPAGAPGDKGETGPSGPAGPTGARGAPGDRGEPGPPGPAGFAGPPGADG

QPGAKGEPGDAGAKGDAGPPGPAGPAGPPGPIGNVGAPGAKGARGSAGPPGATGFPGAAG

RVGPPGPSGNAGPPGPPGPAGKEGGKGPRGETGPAGRPGEVGPPGPPGPAGEKGSPGADG

PAGAPGTPGPQGIAGQRGVVGLPGQRGERGFPGLPGPSGEPGKQGPSGASGERGPPGPMG

PPGLAGPPGESGREGAPGAEGSPGRDGSPGAKGDRGETGPAGPPGAPGAPGAPGPVGPAG

KSGDRGETGPAGPAGPVGPVGARGPAGPQGPRGDKGETGEQGDRGIKGHRGFSGLQGPPG

PPXXXXXXXXXXXXXXXXXXGPPGSAGAPGKDGLNGLPGPIGPPGPRGRTGDAGPVGPPG

PPGPPGPPGPPSGGFDFSFLPQPPQEKAHDGGRYYRARQYDG-KGVGLGPGPMGLMXPRG

PPGAAGAPGPQGFQGPAGEPGEPGQTGPAGARGPAGPPGKAGEDGHPGKPGRPGERGVVG

PQGARGFPGTPGLPGFKGIRGHNGLDGLKGQPGAPGVKGEPGAPGENGTPGQTGARGLPG

ERGRVGAPGPAGARGSDGSVGPVGPAGPIGSAGPPGFPGAPGPKGELGAVGNAGPAGPAG

PRGEVGLPGLSGPVGPPGNPGANGLTGAKGAAGLPGVAGAPGLPGPRGIPGPVGAAGATG

ARGLVGEPGPAGSKGESGNKGEPGSAGPQGPPGPSGEEGKRGPNGEAGSAGPPGPPGLRG

SPGSRGLPGADGRAGVMGPPGSRGASGPAGVRGPSGDAGRPGEPGLMGPRGLPGSPGNIG

PAGKEGPVGLPGIDGRPGPIGPAGARGEPGNIGFPGPKGPTGDPGKNGDKGHAGLAGARG

APGPDGNNGAQGPPGPQGVQGGKGEQGPAGPPGFQGLPGPSGPAGEVGKPGERGLHGEFG

LPGPAGPRGERGPPGESGAAGPTGPIGSRGPSGPPGPDGNKGEPGVVGAVGTAGPSGPSG

LPGERGAAGIPGGKGEKGEPGLRGEIGNPGRDGARGAPGAVGAPGPAGATGDRGEAGAAG

PAGPAGPRGSPGERGEVGPAGPNGFAGPAGAAGQPGAKGERGTKGPKGENGVVGPTGPVG

AAGPAGPNGPPGPAGSRGDGGPPGMTGFPGAAGRTGPPGPSGISGPPGPPGPAGKEGLRG

PRGDQGPVGRTGEVGAVGPPGFAGEKGPSGEAGTAGPPGTPGPQGLLGAPGILGLPGSRG

ERGLPGVAGAVGEPGPLGIAGPPGARGPPGAVGSPGVNGAPGEAGRDGNPGNDGPPGRDG

QPGHKGERGYPGNIGPVGAAGAPGPHGPVGPAGKHGNRGETGPSGPVGPVGAVGPRGPSG

PQGIRGDKGEPGEKGPRGLPGLKGHNGLQGLPGLAGHHGDQGAPGSVGPAGPRGPAGPSG

PAGKDGRTGHPGTVGPAGIRGPQGHQGPAGPPGPPGPPGPPGVSGGGYDFGYDGDFYRA

>GORILLA

QLSYGYDEKSAGGISVPGPMGPSGPRGLPGPPGAPGPQGFQGPPGEPGEPGASGPMGPRG

PPGPPGKNGDDGEAGKPGRPGERGPPGPQGARGLPGTAGLPGMKGHRGFSGLDGAKGDAG

PAGPKGEPGSPGENGAPGQMGPRGLPGERGRPGAPGPAGARGNDGATGAAGPPGPTGPAG

PPGFPGAVGAKGEAGPQGPRGSEGPQGVRGEPGPPGPAGAAGPAGNPGADGQPGAKGANG

APGIAGAPGFPGARGPSGPQGPGGPPGPKGNSGEPGAPGSKGDTGAKGEPGPVGVQGPPG

PAGEEGKRGARGEPGPTGLPGPPGERGGPGSRGFPGADGVAGPKGPAGERGSPGPAGPKG

SPGEAGRPGEAGLPGAKGLTGSPGSPGPDGKTGPPGPAGQDGRPGPPGPPGARGQAGVMG

FPGPKGAAGEPGKAGERGVPGPPGAVGPAGKDGEAGAQGPPGPAGPAGERGEQGPAGSPG

FQGLPGPAGPPGEAGKPGEQGVPGDLGAPGPSGARGERGFPGERGVQGPPGPAGPRGANG

APGNDGAKGDAGAPGAPGSQGAPGLQGMPGERGAAGLPGPKGDRGDAGPKGADGSPGKDG

VRGLTGPIGPPGPAGAPGDKGESGPSGPAGPTGARGAPGDRGEPGPPGPAGFAGPPGADG

QPGAKGEPGDAGAKGDAGPPGPAGPAGPPGPIGNVGAPGAKGARGSAGPPGATGFPGAAG

RVGPPGPSGNAGPPGPPGPAGKEGGKGPRGETGPAGRPGEVGPPGPPGPAGEKGSPGADG

PAGAPGTPGPQGIAGQRGVVGLPGQRGERGFPGLPGPSGEPGKQGPSGASGERGPPGPMG

PPGLAGPPGESGREGAPGAEGSPGRDGSPGAKGDRGETGPAGPPGAPGAPGAPGPVGPAG

KSGDRGETGPAGPAGPVGPVGARGPAGPQGPRGDKGETGEQGDRGIKGHRGFSGLQGPPG

PPGSPGEQGPSGASGPAGPRGPPGSAGAPGKDGLNGLPGPIGPPGPRGRTGDAGPVGPPG

PPGPPGPPGPPSGGFDFSFLPQPPQEKAHDGGRYYRARQYDG-KGVGLGPGPMGLMGPRG

PPGAAGAPGPQGFQGPAGEPGEPGQTGPAGARGPAGPPGKAGEDGHPGKPGRPGERGVVG

PQGARGFPGTPGLPGFKGIRGHNGLDGLKGQPGAPGVKGEPGAPGENGTPGQTGARGLPG

ERGRVGAPGPAGARGSDGSVGPVGPAGPIGSAGPPGFPGAPGPKGELGAVGNAGPAGPSG

PRGEVGLPGLSGPVGPPGNPGANGLTGAKGAAGLPGVAGAPGLPGPRGIPGPVGAAGATG

ARGLVGEPGPAGSKGESGNKGEPGSAGPQGPPGPSGEEGKRGPNGEAGSAGPPGPPGLRG

SPGSRGLPGADGRAGVMGPPGSRGASGPAGVRGPNGDAGRPGEPGLMGPRGLPGSPGNIG

PAGKEGPVGLPGIDGRPGPIGPAGARGEPGNIGFPGPKGPTGDPGKNGDKGHAGLAGARG

APGPDGNNGAQGPPGPQGVQGGKGEQGPAGPPGFQGLPGPSGPAGEVGKPGERGLHGEFG

LPGPAGPRGERGPPGESGAAGPTGPIGSRGPSGPPGPDGNKGEPGVVGAVGTAGPSGPSG

LPGERGAAGIPGGKGEKGEPGLRGEIGNPGRDGARGAPGAVGAPGPAGATGDRGEAGAAG

PAGPAGPRGSPGERGEVGPAGPNGFAGPAGAAGQPGAKGERGAKGPKGENGVVGPTGPVG

AAGPAGPNGPPGPAGSRGDGGPPGMTGFPGAAGRTGPPGPSGISGPPGPPGPAGKEGLRG

PRGDQGPVGRTGEVGAVGPPGFAGEKGPSGEAGTAGPPGTPGPQGLLGAPGILGLPGSRG

ERGLPGVAGAVGESGPLGIAGPPGARGPPGAVGSPGVNGAPGEAGRDGNPGNDGPPGRDG

QPGHKGERGYPGNIGPVGAAGAPGPHGPVGPAGKHGNRGETGPSGPVGPAGAVGPRGPSG

PQGIRGDKGEPGEKGPRGLPGLKGHNGLQGLPGLAGHHGDQGASGSVGPAGPRGPAGPSG

PAGKDGRTGHPGTVGPAGIRGPQGHQGPAGPPGPPGPPGPPGVSGGGYDFGYDGDFYRA

>SAIMIRI

QLSYGYDEKSTGGISVPGPMXXXXXXXXXXXXXXXGPQGFQGPPGEPGEPGASGPMGPRG

PPGPPGKNGDDGEAGKPGRPGERGPPGPQGARGLPGTAGLPGMKGHRGFSGLDGAKGDAG

PAGPKGEPGSPGENGAPGQMGPRGLPGERGRPGAPGPAGARGNDGATGAAGPPGPTGPAG

PAGFPGAVGAKGEAGPQGPRGSEGPQGVRGEPGPPGPAGAAGPAGNPGADGQPGAKGANG

APGIAGAPGFPGARGPSGPQGPSGPPGPKGNSGEPGAPGSKGDTGAKGEPGPVGVQGPPG

PAGEEGKRGARGEPGPTGLPGPPGERGGPGSRGFPGADGVAGPKGPAGERGSPGPAGPKG

SPGEAGRPGEAGLPGAKGLTGSPGSPGPDGKTGPPGPAGQDGRPGPPGPPGARGQAGVMG

FPGPKGAAGEPGKAGERGVPGPPGAVGPAGKDGEAGAQGPPGPAGPAGERGEQGPAGSPG

FQGLPGPAGPPGEAGKPGEQGVPGDLGAPGPSGARGERGFPGERGVQGPPGPAGPRGANG

APGNDGAKGDAGAPGAPGSQGAPGLQGMPGERGAAGLPGPKGDRGDAGPKGADGSPGKDG

VRGLTGPIGPPGPAGAPGDKGETGPSGPAGPTGARGAPGDRGEPGPPGPAGFAGPPGADG

QPGAKGEPGDAGAKGDAGPPGPAGPAGPPGPIGNVGAPGPKGARGSAGPPGATGFPGAAG

RVGPPGPSGNAGPPGPPGPAGKEGGKGPRGETGPAGRPGEVGPPGPPGPAGEKGSPGADG

PAGAPGTPGPQGIAGQRGVVGLPGQRGERGFPGLPGPSGEPGKQGPSGASGERGPPGPMG

PPGLAGPPGESGREGAPGAEGSPGRDGSPGPKGDRGETGPAGPPGAPGAPGAPGPVGPAG

KSGDRGETGPAGPAGPVGPVGARGPAGPQGPRGDKGETGEQGDRGIKGHRGFSGLQGPPG

PPGSPGEQGPSGASGPAGPRGPPGSAGAPGKDGLNGLPGPIGPPGPRGRTGDAGPVGPPG

PPGPPGPPGPPSGGFDFSFLPQPPQEKAHDGGRYYRARXXXX?XX?X?XXXXXXXXXXXX

XXXXXXXXGPQGFQGPAGEPGEPGQTXXXXXXXXXXXXXXXXXXGHPGKPGRPGERGVVG

PQGARGFPGTPGLPGFKGIRGHNGLDGLKGQPGAPGVKGEPGAPGENGTPGQXGARGLPG

ERGRVGAPGPAGARGSDGSVGPVGPAGPIGSAGPPGFPGAPGPKGELGAIGNPGAAGPAG

PRGEVGLPGLSGPVGPPGNPGANGLTGAKGAXGLPGVAGAPGLPGPRGIPGPVGAAGATG

ARGLVXXXXXXXXXXXXXXXXXXGSAGPQGPPGPSGEEGKRGPNGEAGSAGPPGPPGLRG

SPGSRGLPGADGRAGVMGPAGSRGASGPAGVRGPSGDAGRPGEPGLMGPRXXXXXXXXXX

XXXXXXXXGLPGIDGRPGPIGPAGARGEPGSIGFPGPKGPXXXXXXXXXXXXXXXXXXXG

APGPDGNNGAQGPPGPQGVQGGKGEQGPAGPPGFQXXXXXXXXXXXXXXXXXXXXXXXXX

XXXXXXXXGERGPPGESGAAGPAGPIGSRGPSGPPGPDGNKGEPGVVGAAGTAGPSGPGG

LPGERGAAGIPGGKGEKGEPGLRGEIGNPGRDGARXXXXXXXXXXXXXXXXXXXXXXXXX

XXXXXXXXXXXGERGEVGPAGPNGFAGPAGAAGQPGAKGERGAKGPKGENGVVGPTGPVG

AAGPSGPNGPPGPAGSRGDGGPPGMTGFPGAAGRTGPPGPSGISGPPGPPGPAGKEGLRG

PRGDQGPVGRSGETGAVGPPGFAGEKGPSGEAGTXGPPGTPGPQGLLGAPGILGLPGSRG

ERGLPGVAGALGEPGPLGIAGPPGARGPPGAVGSPGVNGAPGEAGRDGNPGNDGPPGRDG

QPGHKGERGYPGNIGPVGAAGAPGPHGPVGPAGKHGNRGEXXXXXXXXXXXXXXXXXXXG

PQGIRGDKGEPGDKGPRGLPGLKGHNGLQGLPGLAXXXXXXXXXXXXXXXXPXGPAGPSG

PAGKDGRTGHPGTVGPAGIRGPQGHQGPAGPPGPPGPPGPPGVSGGGYDFGYDGDFFRA

>CRICETULUS

QMSYGYDEKSAG-VSVPGPMGPSGPRGLPGPPGAPGPQGFQGPPGEPGEPGASGPMGPRG

PPGPPGKNGDDXXXXXXXXXXXXXXXXXQGARGLPGTAGLPGMKGHRGFSGLDGAKGDAG

PAGPKGEPGSPGENGAPGQMGPRGLPGERGRPGAPGPAXXXXXXXXXXXXXXXXXXXXXX

XXXXXXXXXXXGEAGPQGARGSEGPQGVRGEPGPPGPAGAAGPAGNPGADGQPGAKGANG

APGIAGAPGFPGARGPSGPQGPSGAPGPKGNSGEPGAPGNKGDTGAKGEPGPAGVQGPPG

PAGEEGKRGARGEPGPTGLPGPPGERGGPGSRGFPGADGVAGPKGPAGERGSPGPAGPKG

SPGEAGRPGEAGLPGAKGLTGSPGSPGPDGKTGPPGPAGQDGRPGPPGPPGARGQAGVMG

FPGPKGTAGEPGKAGERGVPGPPGPVGPAGKDGEAGAQGAPGPAGPAGERGEQGPAGSPG

FQGLPGPAGPPGEAGKPGEQGVPGDLGAPGPSGARGERGFPGERGVQGPPGPAGPRGNNG

APGNDGAKGDTGAPGAPGSQGAPGLQGMPGERGAAGLPGPKGDRGDAGPKGADGSPGKDG

VRGLTGPIGPPGPAGAPGDKGETGPSGPAGPTGARGAPGDRGEPGPPGPAGFAGPPGADG

QPGAKGEPGETGTKGDSGPPGPAGPAGPPGPIXXXXXXXXXXXXXXXXXXGATGFPGAAG

RVGPPGPSGNAGPPGPPGPVGKEGGKGPRGETGPAGRPGEVGPPGPPGPAGEKGAPGADG

PAGSPGTPGPQGIAGQRGVVGLPGQRGERGFPGLPGPSGEPGKQGPSGSSGERGPPGPMG

PPGLAGPPGESGREGSPGAEGSPGRDGAPGPKGDRGETGPAGPPGAPGAPGAPGPVGPAG

KSGDRGETGPAGPAGPIGPAGARGPAGPQGPRGDKGETGEQGDRGIKGHRGFSGLQGPPG

SPGSPGEQGPSGASGPAGPRGPPGSAGAPGKDGLNGLPGPIGPPGPRGRTGDSGPAGPPG

PPGPPGPPGPPSGGYDFSFLPQPPQEKSHDGGRYYRARXXXX?XX?X?XXXXXGLMGPRG

PPGAVGAPGPQGFQGPAGEPGEPGQTGPAGSRGPAGPPGKAGEDGHPGKAGRPGERGVVG

PQGARGFPGTPGLPGFKGIRGHNGLDGLKGQPGAQGVKGEPGAPGENGTPGQAGARGLPG

ERGRVGAPGPAGARGSDGSVGPVGPAGPIGSAGPPGFPGAPGPKGELGPVGNPGPSGPAG

PRGEVGLPGLSGPVGPPGNPGANGLTGAKGAXGLPGVAGAPGLPGPXXXXXXXXXXXXXX

XXXXXGEPGPAGSKGETGNKGEPGSAGAQGPPGPSGEEGKRGSPGEPGSAGPGGPPGLRG

SPGSRGLPGADGRAGVMGPPGNRGSTGPAGGRGPNGDPGRPGEPGLMGPRGLPGSPGNVG

PSGKEGPVGLPGIDGRPGPIGPAGARGEAGNIGFPGPKGPSXXXXXXXXXXXXXXXXXXG

APGPDGNNGAQGPPGPQGVQGGKGEQGPAGPPGFQGLPGPSGTAGEVGKPGERGLPGEFG

LPGPAGPRGERGPPGESGAAGPSGPIGSRGPSGAPGPDGNKGEAGAVGAPGNAGASGPGG

LPGERGAAGVPGGKGEKGETGLRGEIGNPGRDGARXXXXXXXXXXXXXXXXXXGEAGAAG

PSGPAGPRGSPGERGEVGPAGPNGFAGPAGAAGQPGAKGEKGTKGPKGENGVVGPAGPVG

AAGPSXXXXXXXXXXXXXXXXXXGMTGFPGAAGRTGPPGPSGITGPPGPPGAAGKEGTRG

PRGDQGPVGRTGETGASGPPGFTGEKGPSGEPGXXGPPGSPGPQGFLGPPGILGLPGSRG

ERGLPGVAGALGEPGPLGIAGPPGARGPPGAVGSPGVNGAPGEAGRDGNPGSDGAPGRDG

QPGHKGERGYPGNIGPTGAAGAPGPHGSVGPAGKHGNRGEPXXXXXXXXXXXXXXXXXXG

PQGIRGDKGEPGDKGARGLPGFKGHNGLQGLPGLAXXXXXXXXXXXXXXXXXXGPAGPSG

PVGKDGRSGHPGPVGPAGVRGSQGSQGPXGPPGPPGPPGPPGVSGGGYDFGFEGDFYRA

>HETEROCEPHALUS

QMSYGYDEKSVG-AAVPGPMXXXXXXXXXXXXXXXGPQGFQGPPGEPGEPGASGPMGPRG

PPGPPGKNGDDGEAGKPGRPGERGPPGPQGARGLPGTAGLPGMKGHRXXXXXXXXXXXXX

XXXXXGEPGSPGENGAPGQMGPRGLPGERGRPGPPGPAXXXXXXXXXXXXXXXXXXXXXX

XXXXXXXXXXXGESGPQGARGSEGPQGARGEPGPPGPAGAAGPAGNPGADGQPGAKGANG

APGIAGAPGFPGARGPSGPQGPSGAPGPKGNSGEPGAPGNKGDTGAKGEPGPVGVQGPPG

PAGEEGKRGARGEPGPAGLPGPPGERGGPGSRGFPGADGVAGPKGPSGERGAPGPAGPKG

SPGEAGRPGEAGLPGAKGLTGSPGSPGPDGKTGPPGPAGQDGRPGPPGPPGARGQAGVMG

FPGPKGAAGEPGKAGERGVPGPPGAVGPAGKDGEAGAQGAPGPAGPAGERGEQGPAGSPG

FQGLPGPAGPPGEAGKPGEQXXXXXXXXXXXXXXXGERGFPGERGVQGPPGPAGPRGNNG

APGNDGAKGDAGAPGAPGSQGAPGLQGMPGERGAAGLPGPKGDRGDAGPKGADGTPGKDG

PRGLTGPIGPPGPAGASGDKGESGPSGPAGPTGARGAPGDRGEPGPPGPAGFAGPPGADG

QPGAKGEPGDAGAKGDAGPPGPAGPAGPPGPIXXXXXXXXXXXXXXXXXXGATGFPGAAG

RVGPPGPSGNAGPPGPPGPGGKEGAKGVRGETGPAGRPGEAGPPGPPGPAGEKGSPGADG

PAGAPGTPGPQGIAGQRGVVGLPGQRGERGFPGLPGPSGEPGKQGPSGSSGERGPPGPMG

PPGLAGPPGESGREGSPGAEGSPGRDGSPGPKGERGETGPAGPPGAPGAPGAPGPVGPAG

KSGDRGETGPAGPAGPIGPAGARGPAGPQGPRGDKGETGEQGDRGIKGHRGFSGLQGPPG

PPGSPGEQGPSGASGPAGPRGPPGSAGSPGKDGLNGLPGPIGPPGPRGRTGDAGPXGPAG

PPGPPGPPGPPSGGYDLSFLPQPSQEKAGDGXXXXXXRXXXX?XX?X?XXXXXGLMGPRG

PPGAVGAPGPQGFQGPAGEPGEPGQTXXXXXXXXXXXXXXXXXXGHPGKPGRPGERGVVG

PQGARGFPGTPGLPGFKGPRXXXXXXXXXXXXXXXXXXGEPGAPGENGTPGQAXXXXXXX

XXXXXXXXXXXGARGSDGSVGPVGPAGPIGAAGPPGFPGAPGPKGELGPVGNTGPSGPAG

PRGEVGLPGLSGPVGPPXXXXXXXXXXXXXXXGLPGVAGAPGLPGPRGIPGPPGAAGATG

ARGLVGDPGPAGSKGETGNKGEPGSAGPQGPPGPSGEEGKRGSNGEAGSAGPPGPSGLRG

SPGSRGLPGADGRAGVMGPPGSRGASGPAGVRGPNGDAGRPGEPGLMGPRGLPGSPGNVG

PAGKEGPVGLPGIDGRPGPIGPAGARGEAGNIGFPGPKGPXXXXXXXXXXXXXXXXXXXG

APGPDGNNGAQGPPGPQGVQGGKGEQGPAGPPGFQGLPGPSGPTGEVGKPGERGLPGEFG

LPGPAGPRGERGPPGESGAVGPSGPIGSRGPSGPPGPDGNKGEPGVAGAPGTAGASGPGG

LPGERGAVGIPGGKGEKXXXXXXXXXXXXXXXXXXGAPGAIGAPGPAGATGDRXXXXXXX

XXXXXXXXXXXGERGEVGPAGPNGFAGPAGAAGQPGAKGERGTKGPKGENGGVGPTGPVG

AAGPSGPNGPPGPAGSRGDGGPPGMTGFPGAAGRTGPPGPXGITGPPGPPGPAGKEGLRG

PRGDQGPAGRAGDTGAGGPPGFAGEKGPSGEPGTXGPPGTPGPQGLLGAPGILGLPGSRG

ERGLPGIAGTLGEPGPLGIAGPPGARGPPGNVGNPGVSGAPGEAGRDGNPGNDGPPGRDG

QPGHKGERGYPGNIGPTGTAGAPGPHGPVGPAGKHGNRGEPGPAGSVGPVGAVGPRGPSG

PQGIRGDKGEAGDKGARGLPGMKGHNGLQGLPGLAXXXXXXXXXXXXXXXXXXGPAGPSG

PAGKDGHSGQPGAVGPAGVRGSQGSQGPAXXXXXXXXXXXXXXXXXXXXXXXXXXXXXX

>PAPIO

QLSYGYDEKSTGGISVPGPMGPSGPRGLPGPPGAPGPQGFQGPPGEPGEPGASGPMGPRG

PPGPPGKNGDDGEAGKPGRPGERGPPGPQGARGLPGTAGLPGMKGHRGFSGLDGAKGDAG

PAGPKGEPGSPGENGAPGQMGPRGLPGERGRPGAPGPAGARGNDGATGAAGPPGPTGPAG

PPGFPGAVGAKGEAGPQGARGSEGPQGVRGEPGPPGPAGAAGPAGNPGADGQPGAKGANG

APGIAGAPGFPGARGPSGPQGPGGPPGPKGNSGEPGAPGSKGDTGAKGEPGPVGVQGPPG

PAGEEGKRGARGEPGPTGLPGPPGERGGPGSRGFPGADGVAGPKGPAGERGSPGPAGPKG

SPGEAGRPGEAGLPGAKGLTGSPGSPGPDGKTGPPGPAGQDGRPGPPGPPGARGQAGVMG

FPGPKGAAGEPGKAGERGVPGPPGAVGPAGKDGEAGAQGPPGPAGPAGERGEQGPAGSPG

FQGLPGPAGPPGEAGKPGEQGVPGDLGAPGPSGARGERGFPGERGVQGPPGPAGPRGANG

APGNDGAKGDAGAPGAPGSQGAPGLQGMPGERGAAGLPGPKGDRGDAGPKGADGSPGKDG

VRGLTGPIGPPGPAGAPGDKGETGPSGPAGPTGARGAPGDRGEPGPPGPAGFAGPPGADG

QPGAKGEPGDAGAKGDAGPPGPAGPAGPPGPIGNVGAPGPKGARGSAGPPGATGFPGAAG

RVGPPGPSGNAGPPGPPGPAGKEGGKGPRGETGPAGRPGEVGPPGPPGPAGEKGSPGADG

PAGAPGTPGPQGIAGQRGVVGLPGQRGERGFPGLPGPSGEPGKQGPSGASGERGPPGPMG

PPGLAGPPGESGREGAPGAEGSPGRDGSPGPKGDRGETGPAGPPGAPGAPGAPGPVGPAG

KSGDRGETXXXXXXXXXXXXXXXXXXXXXXXXXXXXXXXXXXXXXXXXXXXXXXXXXXXX

XXGSPGEQGPSGASGPAGPRGPPGSAGTPGKDGLNGLPGPIGPPGPRGRTGDAGPVXXXX

XXXXXXXXXXXXXXXXXXXXXXXXXXXXXXXXXXXXXRQYDG-KGVGLGPGPMGLMGPRG

PPGAAGAPGPQGFQGPAGEPGEPGQTGPAGSRGPAGPPGKAGEDGHPGKPGRPGERGVVG

PQGARGFPGTPGLPGFKGIRGHNGLDGLKGQPGAPGVKGEPGAPGENGTPGQTGARGLPG

ERGRVGAPGPAGARGSDGSVGPVGPAGPIGSAGPPGFPGAPGPKGELGAVGNAGPAGPAG

PRGEVGLPGLSGPVGPPGNPGANGLTGAKGAAGLPGVAGAPGLPGPRGIPGPVGAAGATG

ARGLVGEPGPAGSKGESGNKGEPGSAGPQGPPGPSGEEGKRGPNGEAGSAGPPGPPGLRG

GPGSRGLPGADGRAGVMGPPGSRGASGPAGVRGPNGDAGRPGEPGLMGPRGLPGSPGNIG

PAGKEGPVGLPGIDGRPGPIGPAGARGEPGNIGFPGPKGPTGDPGKNGDKGHAGLAGARG

APGPDGNNGAQGPPGPQGVQGGKGEQGPAGPPGFQGLPGPSGPAGEVGKPGERGLPGDFG

LPGPAGARGERGPPGESGAAGPTGPIGSRGPSGPPGPDGNKGEPGVVGAAGTAGPSGPSG

LPGERGAAGIPGGKGEKGEPGLRGEIGNPGRDGARGAPGAVGAPGPAGATGDRGEAGAAG

PAGPAGPRGSPGERGEVGPAGPNGFAGPAGAAGQPGAKGERGAKGPKGENGVVGPTGPVG

AAGPSGPNGPPGPAGSRGDGGPPGMTGFPGAAGRTGPPGPSGISGPPGPPGPAGKEGLRG

PRGDQGPVGRTGEVGAVGPPGFAGEKGPSGEAGTXGPPGTPGPQGLLGAPGILGLPGSRG

ERGLPGVAGALGEPGPLGIAGPPGARGPPGAVGSPGVNGAPGEAGRDGNPGNDGPPGRDG

QPGHKGERGYPGNNGPVGAAGAPGPHGPVGPAGKHGNRGETGPSGPVGPAGAVGPRGPSG

PQGIRGDKGEPGDKGPRGLPGLKGHNGLQGLPGLAGHHGDQGAPGSVGPAGPRGPAGPSG

PAGKDGRTGHPGTVGPAGIRGPQGHQGPAGPPGPPGPPGPPGVSGGGYDFGYDGDFYRA

>MICROCEBUS

QMSYGYDEKSTG-ISVPGPMGPSGPRGLPGPPGAPGPQGFQGPPGEPGEPGASGPMGPRG

PPGPPGKNGDDGEAGKPGRPGERGPPGPQGARGLPGTAGLPGMKGHRXXXXXXXXXXXXX

XXXXXXXXXXXXXXXXXXXXGPRGLPGERGRPGASGPAGARGNDGATGAAGPPGPTGPAG

PPGFPGAVGAKGEAGPQGARGSEGPQGVRGEPGPPGPAGAAGPAGNPGADGQPGAKGANG

APGIAGAPGFPGARGPSGPQGPSGPPGPKGNSGEPGAPGNKGDAGAKGEPGPAGVQGPPG

PAGEEGKRGARXXXXXXXXXXXXXXXXXXXXXXXXXXXXXXXXXGPAGERGSPGPAGPKG

APGEAGRPGEAGLPGAKGLTGSPGSPGPDGKTGPPGPAGQDGRPGPPGPPGARGQAGVMG

FPGPKGAAGEPGKAGERGVPGPPGAVGPAGKDGEAGAQGAPGPAGPAGERGEQGPAGSPG

FQGLPGPAGPPGESGKPGEQGVPGDLGAPGPSGARGERGFPGERGVQGPPGPAGPRGSNG

APGNDGAKGDAGAPGAPGSQGAPGLQGMPGERGAAGLPGPKGDRGDAGPKGADGSPGKDG

VRGLTGPIGPPGPAGAPGDKGETGPSGPAGPTGARGAPGDRGEPGPPGPAGFAGPPXXXX

XXXXXXXXXXXXXXXXXXXXXXXXXXXXXXXXXXXXXXXXXXXXXXXXXXXXXXXXXXXX

XXXXXXXXXXXXXXXXXXXXXXXXXXXXXXXXXXXXXXXXXXXXXXXXXXXXXXXXXXXX

XXXXXXXXXXXXXXXXXXXXXXXXXXXXXXXXXXXXXXXXXXXXXXXXXXXXXXXXXXXX

XXXXXXXXXXXXXXXXXXXXXXXXXXXXXXXXXXXXXXXXXXXXXXXXXXXXXXXXXXXX

XXXXXXXXXXXXXXXXXXXXGARGPAGPQGPRGDKGETGEQGDRGIKGHRGFSGLQGPPG

PPGSPGEQGPSGASGPAGPRGPPGSAGAAGKDGLNGLPGPIGPPGPRGRTGDAGPVXXXX

XXXXXXXXXXXXXXXXXXXXXXXXXXXXXXXXXXXXXRQYDS-KGVGLGPGPMGLMGPRG

PPGAAGAPGPQGFQGPAGEPGEPGQTGPAGSRGPAGPPGKAGEDGHPGKPGRSGERGVVG

PQXXXXXXXXXXXXXXXXXXGHNGPDGLKGQPGAPGVKGEPGSPGENGTPGQTGARGLPG

ERGRVGAPGPAGARGSDGSVGPVGPAGPIGSAGPPGFPGAPGPKGELGPVGNPGPAGPAG

PRGEVGLPGLSGPVGPPGNPGANGLTGAKGAAGLPGVAGAPGLPGPRGIPGPAGAAGATG

ARGLVGEPGPAGSKGEGGNKGEPGSAGPQGPPGPSGEEGKRGPNGEPGSAGPAGPPGLRG

TPGSRGLPGADGRAGVMGPPGNRGASGPAGGRGPSGDSGRPGEPGLMGPRGLPGSPGNVG

PAGKEGPVGLPGIDGRPGPIGPAGARGEPGNIGFPGPKGPTGDPGKAGDRGHAGLAGARG

APGPDGNNGAQGPPGPQGVQGGKGEQGPAGPPGFQXXXXXXXXXXXXXXXXXXXXXXXXX

XXXXXXXXGERGPPGESGAAGPTGPIGSRGPSGPPGPDGNKGEPGAVGAPGTAGASGPGG

LPGERGAAGIPGGKGEKGESGLRGEIGNPGRDGARGAPGAVGAPGPAGATGDRGEAGAAG

PAGPAGPRGSPGERGEVGPAGPNGFAGPAGAAGQAGAKGERGAKGPKGENGGVGATGPAG

PAGPSGPNGPXGPAGGRGDGGPPGVTGFPGAAGRTGPPGPSGITGPPGPPGAAGKEGLRG

PRGDQGPVGRTGETGASGPPGFAGEKGPSGESGTAGPPGTPGPQGLLGAPGILGLPGSRG

ERGLPGVAGSVXXXXXXXXXXXXXXXXXXXXXXXXXXXXXXXEAGRDGNPGNDGPPGRDG

QAGHKGERGYPGNIGPVGAAGAPGPHGSVGPAGKHGNRGEPGPAGSVGPVGAVGPRGPSG

PQGVRGDKGEAGDKGPRGLPGLKGHAGLQGLPGLAGHHGDQGAPGSVGPAGPRGPAGPSG

PVGKDGRSGHPGTVGPAGIRGPQGHQGPAGPPGPPGPPGPPGAGGGGYDFGFDGDFYRA

>DIPODOMYS

XXXXGYDEKSAG-VSVPGPMGPSGPRGLPGPPGAPGPQGFQGPPGEPGEPGASGPMGPRG

PPGPPGKNGDDGEAGKPGRPGERGPSGPQGARGLPGTAGLPGMKGHRGFSGLDGAKGDAG

PAGPKGEPGSPGENGAPGQMGPRGLPGERGRPGAPGPAGARGNDGATGAAGPPGPTGPAG

PPGFPGAVGAKGEAGPQGARGSEGPQGVRGEPGPPGPAGAAGPAGNPGADGQPGAKGANG

APGIAGAPGFPGARGPSGPQGPSGAPGPKGNSGEPGAPGNKGDTGAKGEPGPAGVQGPPG

PAGEEGKRGARGEPGPAGLPGPPGERGGPGSRGFPGADGVAGPKXXXXXXGSPGPAGPKG

SPGEAGRPGEAGLPGAKGLTGSPGSPGPDGKTGPPGPAGQDGRPGPPGPPGARGQAGVMG

FPGPKGAAGEPGKAGERGVPGPPGAVGPAGKDGEAGAQGPPGPSGPAGERGEQGPAGSPG

FQGLPGPAGPPGEAGKPGDQGVPGDLGAPGPSGARGERGFPGERGVQGPPGPAGPRGSNG

APGNDGAKGDTGAPGAPGSQGAPGLQGMPGERGAAGLPGPKGDRGDAGPKGADGSPGKDG

VRGLTGPIGPPGPAGAPGDKGESGPSGPAGPTGARGAPGDRGEPGPPGPAGFAGPPGADG

QPGAKGEPGDSGAKGDAGPPGPAGPAGPPGPIGNVGAPGPKGARGSAGPPGATGFPGAAG

RVGPPGPSGNAGPPGPPGPVGKEGGKGPRGETGPAGRPGEVGPPGPPGPAGEKGSPGADG

PAGSPGTPGPQGIAGQRGVVGLPGQRGERGFPGLPGPSGEPGKQGPSGASGERGPPGPMG

PPGLAGPPGESGREGSPGAEGXPGRDGSPGPKGDRGETGPAGPPGAPGAPGAPGPVGPAG

KSGDRGETGPAGPAGPIGPVGARGPAGPQGPRGDKGETGETGERGIKGHRGFSGLQGPPG

PPGSPGEQGPSGASGPAGPRGPPGSAGAAGKDGLNGLPGPIGPPGPRGRTGDAGPVGPPG

PPGPPGPPGPPSGGFDFSFMPQPPQEKA?DGGRYYRARQYDG-KGASLGPGPMGLMGPRG

PPGASGAPGPQGFXGPAGEPGEPXQTGPAGARGPPGAPGKAGEDGHPGKPGRPGERGVVG

PQGARGFPGTPGLPGFKGIRGHNGLDGLKGQPGAPGIKGEPGAPGENGTPGQSGARGLPG

ERGRVGAPGPAGARGSDGSVGPVGPAGPIGSAGPPGFPGAPGPKGELGPVGSPGASGPAG

PRGEVGLPGLSGPVGPPGNPGANGLTGSKGAAGLPGVAGAPGLPGPRGIPGPVGAAGATG

PRGLVGEPGPAGSKGETGNKGEPGAAGPQGLPGPSGEEGKRGSNGEPGSAGPAGPPGLRG

NPGSRGLPGADGRAGVMGPPGNRGSSGPAGVRGPNGDSGRPGEPGLMGPRGLPGSPGSVG

PTGKEGPVGLPGIDGRPGPIGPAGARGEAGNIGFPGPKGPTGEPGKHGDKGHPGLAGARG

APGPDGNNGAQGPPGPQGVQGGKGEQGPAGPPGFQGLPGPSGSAGEVGKPGERGLPGEFG

LPGPAGPRGERGPPGESGAAGPSGPIGSRGPSGPPGPDGNKXXXXXXXXXXXXXXXXXXX

XXXXXXXXXXPGGKGEKGETGLRGEIGTPGRDGARXAPAAVGAPAPAGATXXXGEAGAAG

PAGPAGPRGSPGERGEVGPAGPNGFAGPAGAAGQPGAKGERGTKGPKGENGVVGPSGPVG

AAGPSGPNGPPGPVGGRGDGGPPGMTGFPGAAGRTGPPGPSXXXXXXXXXXXXXXXXXXX

XXXXXXXXXXXXXXXXXXXXXXXXXXXXXXXXXXXGPPGTPGPQGLLGAPGILGLPGSRG

ERGLPGISGALGEPGPLGIAGPPGARGPPGAVGSPGVNGAPGEAGRDGNPGSDGPPGRDG

QPGHKGERGYPGNIGPTGAAGAPGPQGSVGPAGKYGNRGEPGPAGSIGPVGAVGPRGPSG

PQGIRGEKGEVGDKGHRGLPGLKGHNGLQGLPGLAGPHGDQGSPGTVGPAGPRGPAGPTG

PVGKDGRSGQPGAVGPAGVRGTQGSQGPAGPPGPPGPPGPPGISGGGYDF??DGDFYRA

>CAVIA

QMSYGYDEKSVG-AAVPGPMGPSGPRGLPGPPGAPGPQGFQGPPGEPGEPGASGPMGPRG

PPGAPGKNGDDGEPGKPGRPGERGPPGPQGARGLPGTAGLPGMKGHRGFSGLDGAKGDAG

PAGPKGEPGSPGENGAPGQMGPRGLPGERGRPGPPGPAGARGNDGATGAAGPPGPTGPAG

PPGFPGAVGAKGESGPQGARGSEGPQGARGEPGPPGPAGAAGPAXXXXXXXXXXXXXXXG

APGIAGAPGFPGARGPSGPQGPSGPPGPKGNSGEPGAPGSKGDTGAKGEPGPVGIQGPPG

PAGEEGKRGARGEPGPAGLPGPPGERGGPGSRGFPGADGVAGPKGPAGERGSPGPAGPKG

SPGEAGRPGEAGLPGAKGLTGSPGSPGPDGKTGPPGPAGQDGRPGPAGPPGARGQAGVMG

FPGPKGAAGEPGKAGERGVPGPPGAVGPAGKDGEAGAQGPPGPAGPAGERGEQGPAGSPG

FQGLPGPAGPPGEAGKPGEQGVPGDLGAPGPSGARGERGFPGERGVQGPPGPAGPRGSNG

APGNDGAKGDAGAPGAPGSQGAPGLQGMPGERGAAGLPGPKGDRGDAGPKGADGTPGKDG

PRGLTGPIGPPGPAGASGDKGETGPSGPPGPTGARGAPGDRGEPGPPGPAGFAGPPGADG

QPGAKGEPGDAGAKGDAGPPGPAGPAGPPGPIGNVGAPGSKGARGSPGPPGATGFPGAAG

RVGPPGPSGNAGPPGPPGPAGKEGAKGVRGETGPAGRPGEAGPPGPPGPAGEKGSPGADG

PAGAPGTPGPQGIAGQRGVVGLPGQRGERGFPGLPGPSGEPGKQGPSGASGERGPPGPAG

PPGLAGPPGESGREXXXXXXXXXXXXXXXXXXGDRGETGPAGPPGAPGAPGAPGPVGPAG

KNGDRGETGPAGPAGPIGPAGARGPAGPQGPRGDKGETGEQGDRGIKGHRGFSGLQGPPG

PPGSPGEQGPSGASGPAGPRGPPGSAGSPGKDGLNGLPGPIGPPGPRGRTGDAGPAGPPG

PPGPPGPPGPPSGGYDLSFLPQPPQEKSGDGGRYYRARQYDG-KGVGLGPGPMGLMGPRG

PPGAVGAPGPQGFQGPAGEPGEPGQTGPAGSRGPAGPPGKAGEDGHPGKPGRPGERGVVG

PQGARGFPGTPGLPGFKGPRGHNGMDGLKGQAGAPGVKGEPGAPGENGTPGQAGARGLPG

ERGRVGAPGPTGARGSDGSVGPVGPAGPIGAAGPPGFPGAPGAKGELGPVGNPGPSGPAG

PRGEVGLPGLSGPVGPPGNPGANGLPGSKGATGLPGVAGAPGLPGPRGIPGPVGAAGATG

ARGLVGDPGPAGSKGESGNKGEPGSAGAQGPPGPSGEEGKRGPNGEVGSAGPPGPPGLRG

SPGSRGLPGADGRSGVMGPPGSRGATGPAGVRGPNGDTGRPGEPGLMGPRGLPGSPGNAG

PAGKEGPMGLPGIDGRPGPIGPAGPRGEAGNIGFPGPKGPTGDPGKSGDKGHPGLAGARG

APGPDGNNGAQGPPGPQGVQGGKGEQGPAGPPGFQGLPGPSGPAGEVGKPGERGLPGEFG

LPGPAGARGERGPPGESGAVGPAGPIGNRGPSGPPGPDGNKGEPGVVGAPGTAGASGPGG

LPGERGAAGIPGGKGEKGETGHRGEPGNTGRDGARGAPGAIGAPGPAGATGDRXXXXXXX

XXXXXXXRGSPGERGEVGPAGPNGFAGPAGAAGQPGAKGERGAKGPKGENGVVGPTGPVG

AAGPSGPNGPPGPAGSRGDGGPPGMTGFPGAAGRTGPPGPSGITGPPGPPGPAGKEGLRG

PRGDQGPVGRTGDTGAGGPPGFAGEKGPSGEPGTAGPPGTPGPQGLLGAPGILGLPGSRG

ERGLPGIAGASGEPGPLGIAGPPGARGPPGNVGSPGVNGPPGEAGRDGNPGNDGPPGRDG

QPGHKGERGYPGNIGPVGAAGAPGPHGPVGPTGKHGNRGEPGPAGSVGPVGAVGPRGPSG

PQGIRGDKGEVGDKGPRGLPGLKGHNGLQGLPGLAGQHGDQGSPGPVGPAGPRGPAGPSG

PAGKDGHAGQPGPVGPAGVRGSQGSQGPAGPPGPPGPPGPAGASGGGYDFGFDGDFYRA

>ORYCTOLAGUS

QMSYGYDEKSAG-VSVPGPMGPSGPRGLPGPPGSPGPQGFQGPPGEPGEPGASGPMGPRG

PPGAPGKNGDDGEAGKPGRPGERGPPGPQGARGLPGTAGLPGMKGHRGFSGLDGAKGDAG

PAGPKGEPGSPGENGAPGQMGPRGLPGERGRPGAPGPAGARGNDGATGAAGPPGPTGPAG

PPGFPGAVGAKGEAGPQGARGSEGPQGVRGEPGPPGPAGAAGPAGNPGADGQPGAKGANG

APGIAGAPGFPGARGPSGPQGPSGPPGPKGNSGEPGAPGNKGDTGAKGEPGPTGVQGPPG

PAGEEGKRGARGEPGPTGLPGPPGERGGPGSRGFPGADGVAGPKGPAGERGAPGPAGPKG

SPGEAGRPGEAGLPGAKGLTGSPGSPGPDGKTGPPGPAGQDGRPGPPGPPGARGQAGVMG

FPGPKGAAGEPGKAGERGVPGPPGAVGPAGKDGEAGAQGPPGPAGPAGERGEQGPAGSPG

FQGLPGPAGPPGEAGKPGEQGVPGDLGAPGPSGARGERGFPGERGVQGPPGPAGPRGSNG

APGNDGAKGDAGAPGAPGSQGAPGLQGMPGERGAAGLPGPKGDRGDAGPKGADGSPGKDG

VRGLTGPIGPPGPAGAPGDKGETGPSGPAGPTGARGAPGDRGEPGPPGPAGFAGPPGADG

QPGAKGEPGDAGAKGDAGPAGPAGPAGPPGPIGNVGAPGPKGARGSPGPPGATGFPGAAG

RVGPPGPSGNAGPPGPPGPVGKEGGKGPRGETGPAGRPGEVGPPGPPGPAGEKGSPGADG

PAGAPGTPGPQGIAGQRGVVGLPGQRGERGFPGLPGPSGEPGKQGPSGASGERGPPGPMG

PPGLAGPPGESGREGSPGAEGSPGRDGAPGPKGDRGETGPAGPPGAPGAPGAPGPVGPAG

KSGDRGETGPAGPAGPIGPAGARGPAGPQGPRGDKGETGEQGDRGIKGHRGFSGLQGPPG

PPGSPGEQGPSGASGPAGPRGPPGSAGAPGKDGLNGLPGPIGPSGPRGRTGDAGPVXXXX

XXXXXXXXXXXXXXXXXXXXXXXXXXXXXXXXXXXXXRQFDG-KG-G-GPGPMGLMGPRG

PPGAAGAPGPQGFQGPAGEPGEPGQTGPAGARGPPGPPGKAGEDGHPGKPGRPGERGVMG

PQGARGFPGTPGLPGFKGIRGHNGLDGLKGQPGAPGVKGEPGAPGENGTPGQTGARGLPG

ERGRVGAPGPAGARGSDGSVGPVGPAGPIGSAGPPGFPGAPGPKGELGPVGNPGPSGPAG

PRGEVGLPGVSGPVGPPGNPGANGLTGAKGAAGLPGVAGAPGLPGPRGIPGPVGAAGATG

ARGLVGEPGPAGTKGESGNKGEPGSAGPQGPPGPSGEEGKRGSPGEPGSAGPAGPPGLRG

SPGSRGLPGADGRAGVMGPPGSRGSTGPAGVRGPNGDSGRPGEPGLMGPRGLPGSPGNVG

PAGKEGPVGLPGIDGRPGPIGPAGARGEPGNIGFPGPKGPTGDPGKNGDKGHPGLAGARG

APGPDGNNGAQGPPGPQGVQGGKGEQGPAGPPGFQGLPGPSGTAGEVGKPGERGLPGEFG

LPGPAGPRGERGAPGESGAAGPPGPIGSRGPSGPPGPDGNKGEPGVVGAPGTAGASGPGG

LPGERGAAGIPGGKGEKGETGLRGEIGNPGRDGARGAPGAVGAPGPAGATGDRGEAGAAG

PAGPAGPRGSPGERGEVGPAGPNGFAGPAGAAGQPGAKGEKGTKGPKGENGVVGPAGPVG

AAGPSGPNGPPGPAGGRGDGGPPGMTGFPGAAGRTGPPGPSGITGPPGPPGAAGKEGLRG

PRGDQGPVGRTGETGASGPPGFPGEKGPSGEAGTAGPPGTPGPQGLLGAPGILGLPGSRG

ERGLPGVAGALGEPGPLGIAGPPGARGPPGAVGSPGVNGAPGEAGRDGNPGSDGPPGRDG

QPGHKGERGYPGNAGPVGAAGAPGPQGSVGPTGKHGNRGEPGPAGSIGPVGAAGPRGPSG

PQGIRGDKGEPGDKGPRGLPGLKGHNGLQGLPGLAGQHGDQGAPGAVGPAGPRGPAGPTG

PAGKDGRSGHPGTVGPAGIRGSQGSQGPAGPPGPPGPPGPPGASGGGYDFGYDGDFYRA

>ICTIDOMYS

QMSYGYDEKSAG-VSVPGPMGPSGPRGLPGPPGAPGPQGFQGPPGEPGEPGASGPMGPRG

PPGAPGKNGDDGEAGKPGRPGDRGPPGPQGARGLPGTAGLPGMKGHRGFSGLDGAKGDAG

PAGPKGEPGSPGENGAPGQMGPRGLPGERGRPGAPGPAGARGNDGATGAAGPPGPTGPAG

PPGFPGAVGAKGEAGPQGARGSEGPQGVRGEPGPPGPAGAAGPAGNPGADGQPGAKGANG

APGIAGAPGFPGARGPSGPQGPSGPPGPKGNSGEPGAPGNKGDPGAKGEPGPTGVQGPPG

PAGEEGKRGARGEPGPAGLPGPPGERGGPGSRGFPGADGVAGPKGPAGERGSPGPAGPKG

SPGEAGRPGEAGLPGAKGLTGSPGSPGPDGKTGPPGPAGQDGRPGPPGPPGARGQAGVMG

FPGPKGAAGEPGKTGERGVPGPPGAVGPAGKDGEAGAQGPPGPAGPAGERGEQGPAGSPG

FQGLPGPAGPPGEAGKPGEQGVPGDLGAPGPSGARGERGFPGERGVQGPPGPAGPRGSNG

APGNDGAKGDAGAPGAPGSQGAPGLQGMPGERGAAGLPGPKGDRGDAGPKGADGSPGKDG

VRGLTGPIGPPGPAGAPGDKGETGPSGPAGPTGARGAPGDRGEAGPPGPAGFAGPPGADG

QPGAKGEPGDAGAKGDAGPPGPAGPAGPPGPIGNVGAPGPKGARGSAGPPGATGFPGAAG

RVGPPGPSGNAGPPGPPGPAGKEGGKGPRGETGPAGRVGEVGPPGPPGPAGEKGSPGADG

PAGAPGTPGPQGIAGQRGVVGLPGQRGERGFPGLPGPSGEPGKQGPSGASGERGPPGPMG

PPGLAGPPGESGREGSPGAEGSPGRDGSPGPKGDRGETGPAGPPGAPGAPGAPGPVGPAG

KSGDRGETGPAGPAGPIGPAGARGPAGPQGPRGDKGETGEQGDRGIKGHRGFSGLQGPPG

PPGSPGEQGPSGASGPAGPRGPPGSAGSPGKDGLNGLPGPIGPPGPRGRTGDAGPVGPPG

PPGPPGPPGPPSGGFDFSFMPQPPQEKAGDG-RYYRARQYDG-KGVGMGPGPMGLMGPRG

PPGAAGAPGPQGFQGPAGEPGEPGQTGPAGARGPPGAPGKAGEDGHPGKPGRPGERGVVG

PQGARGFPGTPGLPGFKGIRGHNGLDGLKGQPGAQGVKGEPGAPGENGTPGQAGARGLPG

ERGRVGAPGPAGARGSDGSVGPVGPAGPIGSAGPPGFPGAPGPKGELGPVGNPGPSGPAG

PRGEVGLPGLSGPVGPPGNPGANGLTGAKGAAGLPGVAGAPGLPGPRGIPGPVGAAGATG

ARGLVGEPGPAGSKGESGNKGEPGSAGPQGPPGPSGEEGKRGPNGEPGSAGPAGPPGLRG

NPGSRGLPGADGRAGVMGPPGNRGATGPAGVRGPNGDSGRPGEPGLMGPRGLPGSPGNVG

PAGKEGPVGLPGIDGRPGPIGPAGARGEAGNIGFPGPKGPTGDPGKSGDKGHPGLAGARG

APGPDGNNGAQGPPGPQGVQGGKGEQGPAGPPGFQGLPGPSGTAGEVGKPGERGLPGEFG

LPGPAGPRGERGPPGESGAVGPAGPIGSRGPSGPPGPDGNKGEPGVVGAPGTAGASGPGG

LPGERGAAGIPGGKGEKGEPGLRGEIGNPGRDGARGAPGAVGAPGPAGATGDRGEAGAAG

PAGPPGPRGSPGERGEVGPAGPNGFAGPAGAAGQPGAKGERGTKGPKGENGVVGPAGPVG

AAGPSGPNGPPGPAGGRGDGGPPGMTGFPGAAGRTGPPGPSGITGPPGPPGAAGKEGLRG

PRGDQGPVGRTGETGASGPPGFAGEKGPAGEPGTAGPPGTPGPQGLLGAPGILGLPGSRG

ERGLPGIAGALGEPGPLGIAGPPGARGPPGAVGSPGVNGAPGEAGRDGNPGSDGPPGRDG

QPGHKGERGYPGNIGPAGAAGAPGPHGTVGPAGKHGNRGEPGPAGSVGPVGAVGPRGPSG

PQGVRGDKGEPGDKGPRGLPGLKGHNGLQGLPGLAGQHGDQGSPGPVGPAGPRGPAGPSG

PVGKDGRSGHPGSVGPAGVRGSQGSQGPAGPPGPPGPPGPPGVSGGGYDFGYEGDFYRA

>MONODELPHIS

QMSYGYDEKSGGGMSVPGPMGPSGPRGLPGPPGNPGPQGFQGPPGEPGEPGASGPMGPRG

PAGPPGKNGDDGEAGKPGRPGERGPPGPQGARGLPGTAGLPGMKGHRGFSGLDGAKGDSG

PAGPKGEPGSPGENGAPGQMGPRGLPGERGRPGPPGPAGARGNDGATGAAGPPGPTGPAG

PPGFPGAVGAKGEAGPQGSRGSEGPQGVRGEPGPPGPAGAAGPSGNPGADGQPGAKGANG

APGIAGAPGFPGARGPSGPQGPSGAPGPKGNSGEPGAPGNKGDPGAKGEPGPVGVQGPPG

PAGEEGKRGSRGEPGPSGLPGPAGERGGPGSRGFPGADGVAGPKGAPGERGAPGPAGPKG

SPGEAGRPGEAGLPGAKGLTGSPGSPGPDGKTGPPGPAGQDGRPGPPGPPGARGQAGVMG

FPGPKGAAGEPGKAGERGVPGPPGAVGAAGKDGEAGAQGPPGPAGPAGERGEQGPAGSPG

FQGLPGPAGPPGEAGKPGEQGVPGDAGAPGPSGARGERGFPGERGVQGPPGPQGPRGSNG

APGNDGAKGDAGAPGAPGGQGPPGLQGMPGERGAAGLPGAKGDRGDAGPKGADGAAGKDG

VRGLTGPIGPPGPAGPTGDKGESGPSGPVGPTGARGAPGERGEPGPPGPAGFAGPPGADG

QPGAKGEPGDAGAKGDAGPPGPAGPTGAPGPAGNVGAPGPKGARGNAGPPGATGFPGAAG

RVGPPGPSGNAGPPGPPGPAGKEGGKGPRGETGPIGRPGEVGPPGPPGPSGEKGSPGADG

PAGAPGTPGPQGIAGQRGVVGLPGQRGERGFPGLPGPSGEPGKQGPSGISGERGPPGPAG

PPGLAGPPGESGREGSPGAEGSPGRDGSPGPKGDRGETGPAGPPGAPGAPGAPGPVGPAG

KSGDRGETGPAGPAGPVGPTGARGPSGPQGPRGDKGETGEQGDRGMKGHRGFSGLQGPPG

PPGSPGEQGPSGASGPAGPRGPPGSAGASGKDGLNGLPGPIGPPGPRGRTGDAGPAGPPG

PPGPAGPPGPPSGGFDFSFLPQPPQEKAHDSGRYYRARXXXX?XX?X?XXXXXXXXXXXX

XXXXXXXXXXXXXXXXXXXXXXXXXXXXXXXXXXXXXXXXXXXXXXXXXXXXXXXXXXXX

XXXXXXXXXXXXXXXXXXXXXXXXXXXXXXXXXXXXXXXXXXXXXXXXXXXXXXXXXXXX

XXXXXXXXXXXXXXXXXXXXXXXXXXXPIGSAGPPGFPGAPGPKGELGPVGNPGPAGPAG

PRGELGLPGMTGPVGPAGNPGANGLTGAKGAAGLPGVAGAPGLPGPRGIPGPAGAAGASG

PRGLAGEPGPAGSKGESGNKGEPGSAGPQGPPGPNGEEGKRGPNGEPGSSGPAGPPGLRG

VPGSRGLPGADGRAGGMGPPGNRGSSGPAGVRGPNGDAGRPGEPGLMGPRGLPGSPGNSG

PTGKEGPAGLPGADGRPGPTGPAGNRGEPGNIGFPGPKGPTGDPGKSGEKGHAGLAGARG

APGPDGNNGAQGPPGPAGVQGGKGEQGPAGPPGFQGLPGPSGPAGEGGKVGERGLAGEFG

LPGPAGPRGERGPPGESGAVGPTGSIGSRGPSGPPGPDGNKGEPGVVGAPGNAGPAGSGG

VPGERGAAGVPGGKGDKGETGPRGEFGNPGRDGARGAPXXXXXXXXXXXXXXXXXXXXXX

XXXXXXXXXXXXXXXXXXXXXXXXXXXXXXXXXXXXXXXXXXXXXXXXXXXXXXXXXXXX

XXXXXXXXXXXXXXXXXXXXXXXXXXXXXXXXXXXXXXXXXXXXXXXXXXXXXXXXXXXX

XXXXXXXXXXXXXXXXXXXXXXXXXXXXXXXXXXXGPPGSSGPQGLLGAPGILGLPGSRG

ERGLPGVSGSLGEPGPLGIAGPPGARGPPGAVGSPGVNGAPGEAGRDGNPGNDGPPGRDG

LSGHKGERGYPGNPGAVGNAGAPGPHGTVGPAGKAGNRGEPXXXXXXXXXXXXXXXXXXX

XXXXXXXXXXXXXXXXXXXXXXXXXXXXXXXXXXXXXXXXXXXXXXXXXXXXXXXXXXXX

XXXXXXXXXXXXXXXXXXXXXXXXXXXXXXXXXXXXXXXXXXXXXXXXXXXXXXXXXXX

>TRICHECHUS

XXXXXXXXXXXX?XXXXXPXGPSGPRGLPGPPGAPGPQGFQGPPGEPGEPGASXXXXXXX

XXXXXXXXXXXGEAGKPGRPGERGPPGPQGARGLPGTAGLPGMKGHRGFSGLDGAKGDAG

PAGPKGEPGSPGENGAPGQMGPRGLPGERGRPGXXXXXGARGNDGATGAAGPPGPTGPAG

PPGFPGAVGAKGEAGPQGSRGSEGPQGVRGEPGPPGPAGAAGPAGNPGADGQPGAKGANG

APGIAGAPGFPGARGPSGPQGPSGAPGPKGNSGEPGAPGSKGDAGAKGEPGPTGIQGPPG

PAGEEGKRGARGEPGPTGLPGPPGERGGPGSRGFPGADGVAGPKGPAGERGSPGPAGPKG

SPGEAGRPGEAGLPGAKGLTGSPGSPGPDGKTGPPGPAGQDGRPGPPGSPGARGQAGVMG

FPGPKGAAGEPGKAGERGVPGPAGAVXXXXXXXXXXXXXXXXXXGPAGERGEQGPAGSPG

FQGLPGPAGPPGEAGKPGEQGVPGDLGAPGPSGARGERGFPGERGVQGPPGPAGPRGSNG

APGNDGAKGDAGAPGAPGSQGAPGLQGMPGERGAAGLPGPKGDRGDAGPKGADGSPGKDG

ARGLTGPIGPPGPAGAPGDKXXXXXXXXXXXXXXXXXXGDRGEPGPPGPAGFAGPPGADG

QPGAKGEPGDAGAKGDAGPPGPAGPTGAPGPIGNVGAPGTKGARGSAGPPGATGFPGAAG

RVGPPGPSGNAGPPGPPGPAGKEGSKGPRGETGPAGRPGEVGPPGPPGVTGEKGSPGADG

PAGAPGTPGPQGIGGQRGVVGLPGQRGERGFPGLPGPSGEPGKQGPSGPGGERGPPGPVG

PPGLAGPPGESGREGSPGAEGSPGRDGSPGPKGDRGESGPAGPPGAPGAPGAPGPVGPAG

KSGDRGETGPAGPAGPVGPAGVRGPAGPQGPRGDKGETGEQGDRGIKGHRGFSGLQGPPG

PPGSPGEQGPSGASGPAGPRGPPGSAGSPGKDGLSGLPGPIGPPGPRGRTGDAGPVGPPG

PPGPPGPPGPPSAGFDFSFLPQPPQEKAHDGGRYYRARXXXX?XX?X?XXXXXGLMGPRG

PPGATGPPXXXXXXXXXXXXXXXXXXGPAGSRGPPGPPGKAGEDGHPGKPGRPGERGVVG

PQGARGFPGTPGLPGFKGIRGHNGLDGLKGQPGAPGVKXXXXXXXXXXXXXXXGARGLPG

ERGRVGGPGPAGARGSDGSVGPVGPAXXXXXXXXXXXXXXXXXXGELGPVGNPGPAGPAG

PRGEVGLSGVSGPVGPPGNPGANGLAGAKGAAGLPGVAGAPGLPGPRGIPGPPGSAGATG

ARGLVXXXXXXXXXXXXXXXXXXGSAGPQGPPGPSGEEGKRGPNGEAGSTGPAGPPGLRV

GXXXXXXXXXXXXXXXXGLPGSRGATGPAGVRGPSGDAGRPGEPGVMGPRGLPGSPGNVG

PAGKEGPAGLPGIDGRLGPAGPTGARGEPGNIGFPGPKGPXGDPGKNGEKGHAGLAGPRG

APGPDGNNGAQGPPGPQGVQGGKGEQGPAGPPGFQGLPGPAGTAGEAGKPGERXXXXXXX

XXXXXXXXGERGPPGPSGATGPAGPTGSRGPSGPPGPDGNKGEPGVVGAPGTAGPSGPSG

LPGERGASGIPGGKGEKXXXXXXXXXXXXXXXXXXGPPGAVGAPGPAGATGDRXXXXXXX

XXXXXXXXXXXGERGEVGPAGPNGFAGPAGAAGQPGAKGERGTKGPKGENGPVGPTGPVG

AVGPAGPNGPPGPAGSRGDGGPPGATGFPGAAGRTGPPGPXGITGPPGPPGPAGKEGLRG

PRGDQGPVGRTGETGASGPLGFTGEKGPPGEPGAAGPPGTPGPQGLLGPPGILGLPGTRG

ERGLPGVAGAVGEPGPLGIAGPAGARGPPGAVGSPGVNGAPGEAGRDGNPGSDGPPGRDG

LPGHKGDRGYPGNAGPVGTAGAPGPHGSVGPAGKQGSRGEPGPAGSVGPVGAVGPRGPXG

PQGIRGDKGEPGEKGPRGLPGLKGHNGLQGLPGLAGQHGDQGSPGTVGPAGPRGPAGPSG

PVGKDGRPGHSGPVGPAGVRGSQGSQGPXXXXXXXXXXXXXXXXXXXXXXXXXXXXXXX

>LOXODONTA

QLSYGYDEKSAGGISVPGPMGPSGPRGLPGPPGAPGPQGFQGPPGEPGEPGASGPMGPRG

PPGPPGKNGDDGEAGKPGRPGERGPPGPQGARGLPGTAGLPGMKGHRGFSGLDGAKGDAG

PAGPKGEPGSPGENGAPGQMGPRGLPGERGRPGAPGPAGARGNDGATGAAGPPGPTGPAG

PPGFPGAVGAKGEAGPQGARGSEGPQGVRGEPGPPGPAGAAGPAGNPGADGQPGAKGANG

APGIAGAPGFPGARGPAGPQGPSGAPGPKGNSGEPGAPGSKGDAGAKGEPGPVGIQGPPG

PAGEEGKRGARGEPGPTGLPGPPGERGGPGSRGFPGADGVAGPKGPAGERGSPGPAGPKG

SPGEAGRPGEAGLPGAKGLTGSPGSPGPDGKTGPPGPAGQDGRPGPPGPPGARGQAGVMG

FPGPKGAAGEPGKAGERGVPGPPGAVGAAGKDGEAGAQGPPGPAGPAGERGEQGPAGSPG

FQGLPGPAGPPGEAGKPGEQGVPGDLGAPGPSGARGERGFPGERGVQGPPGPAGPRGSNG

APGNDGAKGDAGAPGAPGSQGAPGLQGMPGERGAAGLPGPKGDRGDAGPKGADGSPGKDG

PRGLTGPIGPPGPAGAPGDKGEAGPSGPAGPTGARGAPGDRGEPGPPGPAGFAGPPGADG

QPGAKGEPGDAGAKGDAGPPGPAGPTGAPGPIGNVGAPGPKGARGSAGPPGATGFPGAAG

RVGPPGPSGNAGPPGPPGPAGKEGGKGPRGETGPAGRPGEVGPPGPPGPAGEKGSPGADG

PAGAPGTPGPQGIGGQRGVVGLPGQRGERGFPGLPGPSGEPGKQGPSGSSGERGPPGPAG

PPGLAGPPGESGREGAPGAEGSPGRDGSPGPKGDRGETGPSGPPGAPGAPGAPGPVGPAG

KSGDRGETGPAGPAGPAGPAGVRGPAGPQGPRGDKGETGEQGDRGIKGHRGFSGLQGPPG

PPGSPGEQGPSGASGPAGPRGPPGSAGAPGKDGLNGLPGPIGPPGPRGRTGDAGPVGPPG

PPGPPGPPGPPSGAFDFSFLPQPPQEKAHDGGRYYRARQYDA-KGIGLGPGPMGLMGPRG

PPGATGPPGSPGFQGPPGEPGEPGQTGPAGSRGPAGPPGKAGEDGHPGKPGRPGERGVVG

PQGARGFPGTPGLPGFKGIRGHNGLDGLKGQPGAPGVKGEPGAPGENGTPGQIGARGLPG

ERGRVGGPGPAGARGSDGSVGPVGPAGPIGSAGPPGFPGAPGPKGELGPVGNPGPSGPAG

PRGEAGLPGVSGPVGPPGNPGANGLAGAKGAAGLPGVAGAPGLPGPRGIPGPVGAAGATG

ARGLVGEPGPAGSKGESGSKGEPGSAGPQGPPGPSGEEGKRGSSGEAGSAGPAGPPGLRG

GPGSRGLPGADGRAGVMGPPGSRGASGPAGVRGPSGDSGRPGEPGVMGPRGLPGSPGNVG

PAGKEGPAGLPGIDGRPGPIGPAGARGEPGNIGFPGPKGPAGDPGKNGDKGHAGLAGPRG

APGPDGNNGAQGPPGLQGVQGGKGEQGPAGPPGFQGLPGPSGTAGEAGKPGERGLPGEFG

LPGPAGPRGERGPPGQSGAAGPTGPIGSRGPSGPPGPDGNKGEPGVVGAPGTAGPSGPGG

LPGERGAAGIPGGKGEKGETGLRGDTGNTGRDGARGAPGAVGAPGPAGATGDRGEAGPAG

SAGPAGPRGSPGERGEVGPAGPNGFAGPAGAAGQAGAKGERGTKGPKGENGPVGPTGPVG

AAGPAGPNGPPGPAGSRGDGGPPGATGFPGAAGRTGPPGPAGITGPPGPPGAAGKEGLRG

PRGDQGPVGRTGETGASGPPGFAGEKGSSGEPGTAGPPGTPGPQGLLGPPGILGLPGSRG

ERGLPGVAGAVGEPGPLGIAGPPGARGPPGAVGSPGVNGAPGEAGRDGNPGSDGPPGRDG

LPGHKGERGYPGNAGPVGTAGAPGPQGPLGPAGKHGNRGEPGPAGSVGPVGAVGPRGPSG

PQGARGDKGEAGDKGPRGLPGFKGHNGLQGLPGLAGQHGDQGSPGSVGPAGPRGPAGPSG

PVGKDGRPGHAGAVGPAGVRGSQGSQGPSGPPGPPGPPGPPGPSGGGYDFGYDGDFYRA

>CALLITHRIX

QLSYGYDEKSTGGISVPGPMGPSGPRGLPGPPGSPGPQGFQGPPGEPGEPGASGPMGPRG

PPGPPGKNGDDGEAGKPGRPGERGPPGPQGARGLPGTAGLPGMKGHRGFSGLDGAKGDAG

PAGPKGEPGSPGENGAPGQMGPRGLPGERGRPGPPGPAGARGNDGATGAAGPPGPTGPAG

PAGFPGAVGAKGEAGPQGPRGSEGPQGVRGEPGPPGPAGAAGPAGNPGADGQPGAKGANG

APGIAGAPGFPGARGPSGPQGPSGPPGPKGNSGEPGAPGSKGDTGAKGEPGPVGVQGPPG

PAGEEGKRGARGEPGPTGLPGPPGERGGPGSRGFPGADGVAGPKGPAGERGSPGPAGPKG

SPGEAGRPGEAGLPGAKGLTGSPGSPGPDGKTGPPGPAGQDGRPGPPGPPGARGQAGVMG

FPGPKGAAGEPGKAGERGVPGPPGAVGPAGKDGEAGAQGPPGPAGPAGERGEQGPAGSPG

FQGLPGPAGPPGEAGKPGEQGVPGDLGAPGPSGARGERGFPGERGVQGPPGPAGPRGANG

APGNDGAKGDAGAPGAPGSQGAPGLQGMPGERGAAGLPGPKGDRGDAGPKGADGSPGKDG

VRGLTGPIGPPGPAGAPGDKGETGPSGPAGPTGARGAPGDRGEPGPPGPAGFAGPPGADG

QPGAKGEPGDAGAKGDAGPPGPAGPAGPPGPIGNVGAPGPKGARGSAGPPGATGFPGAAG

RVGPPGPSGNAGPPGPPGPAGKEGGKGPRGETGPAGRPGEVGPPGPPGPAGEKGSPGADG

PAGAPGTPGPQGIAGQRGVVGLPGQRGERGFPGLPGPSGEPGKQGPSGTSGERGPPGPMG

PPGLAGPPGESGREGAPGAEGSPGRDGSPGPKGDRGETGPAGPPGAPGAPGAPGPVGPAG

KSGDRGETGPAGPAGPIGPVGSRGPAGPQGPRGDKGETGEQGDRGIKGHRGFSGLQGPPG

PPGSPGEQGPSGASGPAGPRGPPGSAGAPGKDGLNGLPGPIGPPGPRGRTGDAGPVGPPG

PPGPPGPPGPPSGGLDFSFLPQPXXXXAHDGGRYYRARQYDG-KGVGLGPGPMGLMGPRG

PPGAAGAPGPQGFQGPAGEPGEPGQTGPAGARGPPGPPGKAGEDGHPGKPGRPGERGVVG

PQGARGFPGTPGLPGFKGIRGHNGLDGLKGQPGAPGVKGEPGAPGENGTPGQTGARGLPG

ERGRVGAPGPAGARGSDGSVGPVGPAGPIGSAGPPGFPGAPGPKGELGAIGNPGIAGPAG

PRGEVGLPGLSGPVGPPGNPGANGLTGAKGAAGLPGVAGAPGLPGPRGIPGPVGAAGATG

ARGLVGEPGPAGSKGESGNKGEPGSAGPQGPPGPSGEEGKRGPNGEAGSAGPPGPPGLRG

SPGSRGLPGADGRAGVMGPAGSRGATGPAGVRGPNGDAGRPGEPGLMGPRGLPGSPGNIG

PAGKEGPVGLPGIDGRPGPIGPAGARGEPGSIGFPGPKGPTGDPGKNGDKGHAGLAGARG

APGPDGNNGAQGPPGPQGVQGGKGEQGPAGPPGFQGLPGPSGPAGELGKPGERGLPGEFG

LPGPAGPRGERGPPGESGAAGPTGPIGSRGPSGPPGPDGNKGEPGVVGAAGTAGPSGPSG

LPGERGAAGIPGGKGEKGEPGLRGEIGNPGRDGARGAPGAVGAPGPAGATGDRGEAGAAG

PAGPAGPRGSPGERGEVGPAGPNGFAGPAGAAGQPGAKGERGAKGPKGENGVVGPTGPVG

AAGPXXXXXPPGPAGSRGDGGPPGMTGFPGAAGRTGPPGPSGISGPPGPPGPAGKEGLRG

PRGDQGPVGRTGETGAVGPPGFAGEKGPSGEAGTAGPPGTPGPQGLLGAPGILGLPGSRG

ERGLPGVAGAVGEPGPLGIAGPPGARGPPGAVGSPGVNGAPGEAGRDGNPGNDGPPGRDG

QPGHKGERGYPGNIGPVGAAGAPGPHGPVGPAGKHGNRGETGPSGPVGPAGAVGPRGPSG

PQGIRGDKGEPGDKGPRGLPGLKGHNGLQGLPGLAGHHGDQGAPGSVGPAGPRGPAGPSG

PAGKDGRTGHPGTVGPAGIRGPQGHQGPAGPPGPPGPPGPPGVSGGGYDFGYDGDFFRA

>MACROPUS

QMSYGYDEKSGG-ISVPGPMXXXXXXXXXXXXXXXXXXXXXXXXXXXXXXXXXXXXXXXX

XXXXXXXXXXXGEAGKPGRPGERGPPGPQGARGLPGTAGLPGMKGHRGFSGLDGAKGDSG

PAGPKGEPGSPGENGAPGQMGPRGLPGERGRPGPPGPAGARGNDGATGAAGPPGPTGPAG

PPGFPGAVGAKGEAGPQGARGSEGPQGVRGEPGPPGPAGAAGPSGNPGADGQPGAKGANG

APGIAGAPGFPGARGPSGPQGPSGAPGPKGNSGEPGAPGNKGDAGAKGEPGPVGVQGPPG

PAGEEGKRGSRGEPGPTGLPGPAGERGGPGSRGFPGADGVAGPKGAPGERGAPGPAGPKG

SPGESGRPGEAGLPGAKGLTGSPGSPGPDGKTGPPGPAGQDGRPGPPGPPGARGQAGVMG

FPGPKGAAGEPGKAGERGVPGPPGAVGAAGKDGEAGAQGPPGPAGPAGERGEQGPAGSPG

FQGLPGPAGPPGEAGKPGEQGVPGDAGAPGPSGARGERGFPGERGVQGPPGPQGPRGANG

APGNDGAKGDAGAPGAPGSQGPPGLQGMPGERGAAGLPGAKGDRGDAGPKGADGAPGKDG

VRGLTGPIGPPGPAGPSGDKGESGPSGPVGPTGARGAPGERGEPGPPGPAGFAGPPGADG

QPGAKGEPGDAGAKGDAGPPGPAGPTGAPGPAGNVGAPGPKGARGSAGPPGATGFPGAAG

RVGPPGPSGNTGPPGPPGPAGKEGGKGPRGETGPVGRPGEVGPPGPPGPSGEKGSPGADG

PAPVPXXXXXXXXXXXXXXXXXXXXXXXXXXXXXXXXXXXXXXXXXSGVSGERGPPGPAG

PPGLAGPPGESGREGAPGAEGSPGRDGAPGAKGDRGETGPAGPPGAPGAPGAPGPVGPAG

KAGDRGETXXXXXXXXXXXXXXXXXXGPQGPRGDKGETGEQGDRGIKGHRGFSGLQGPPG

PPGSPGEQGPSGASGPAGPRXXXXXXXXXXXXXXXXXXXXXXXXXXXXXXXXXXXXGPPG

PPGPPGPPGPPSGGFDFSFLPQPPQEKAHDSGRYYRARQYDASKGIDMGPGPMGLMGPRG

PRGASGPPGAQGFQGPAGEPGEPGQTGPAGARGPPGPPGKSGEDGHPGKPGRPGERGIVG

PQXXXXXXXXXXXXXXXXXXXXXXXXXXXXXXXXXXXXGEPGAPGENGTPGQAGARGLPG

ERGRIGGAGPAGARGSDGSVGPVGPAGPIGSAGPPGFPGAPGPKGELGPVGNPGPAGPAG

PRGELGLPGMTGPVGPAGNPGANGLTGAKGAAGLPGVAGAPGLPGPRGIPGPAGAAGASG

PRGLAGEPGPAGAKGESGNKGEPGAAGPQGPPGPSGEEGKRGPNGEPGSTGPTGPPGLRG

VPGSRGLPGADGRAGGMGPPGNRGSSGPAGARGPNGDAGRPGEPGLMGPRGLPGSPGNPG

PTGKEGPAGLPGPDGRPGPTGPAGNRGEPGNIGFPGPKGPNGEPGKSGEKGHAGLAGARG

APGPDGNNGAQGPPGPAGVQGGKGEQGPAGPPGFQGLPGPSGPAGEGGKVGERGLPGEFG

LPGPAGPRXXXXXXXXXXXXXXXXXXXXXXXXXXXXXXXXXGEPGVVGAPGSAGPAGSGG

VPGERGAAGVPGGKGEKGETGLRGDFGNPGRDGARGAPGAMGAPGPAGATGERGEAGPAG

PVGPTGARGAPGDRGEAGPAGPNGFAGPPGAAGQAGAKGERGTKGPKGENGVVGPTGPVG

AAGPAGPNGPPGPVGGRGDGGPPGATGFPGAAGRTGAPGPAGITGPPGPPGASGKEGPRG

PRGDQGPLGRAGETGAVGPPGFAGEKGPPGEAGATGPPGSSGPQGLLGAPGILGLPGSRG

ERGLPGVSGALGEPGPLGIAGPPGARGPPGAVGNPGVNGAPGEAGRDGNPGNDGPPGRDG

LAGHKGERGYPGNAGAVGNAGAPGPHGTVGPAGKPGNRGEPGPVGSVGPAGPFGARGPSG

PQGPRGDKGEVGDKGPRGLNGLKGHNGFQGLPGLAGQHGDQGAPGSIGPAGPRGPAGPSG

PAGKDGRPGQAGAVGPAGIRGSQGSQGPAGPPGPPGLPGPPGPSGGGYDFGYDGDFYRA

>PROCAVIA

QLSYGYDEKSAGGISVPGPMGPSGPRGLPGPPGAPGPQGFQGPPGEPGEPGASGPMGPRG

PPGPPGKNGDDGEAGKPGRPGERGPPGPQGARGLPGTAGLPGMKGHRGFSGLDGAKGDAG

PAGPKGEPGSPGENGAPGQMGPRGLPGERGRPGPPGPAGARGNDGAAGAAGPPGPTGPAG

PPGFPGAVGAKGEGGPQGPRGSEGPQGVRGEPGPPGPAGAAGPAGNPGADGQPGAKGANG

APGIAGAPGFPGARGPSGPQGPSGAPGPKGNSGEPGAPGSKGDAGAKGEPXXXXXXXXXX

XXXXXXXXXXXXXXXXXXXXXXXXXXXXXXXXXXXXXXXXXXXXXXXXXXXXXXXXXXXX

XXXXXXXXXXXXXXXXXXXXXXXXXXXXXXXXXXXXXXXXXXXXXXXXXXXXXXXXXXXX

XXXXXXXXGEPGKAGERGVPGPAGAVGAPGKDGEAGAQGPPGPAGPAGERGEQGPAGSPG

FQGLPGPAGPPGEAGKPGEQGVPGDLGAPGPSGARGERGFPGERGVQGPPGPAGPRGSNG

APGNDGAKGDAGAPGAPGSQGAPGLQGMPGERGAAGLPGPKGDRGDAGPKGADGXXXXXX

XXXXXXXXXXXXXXXXXXXXXXXXXXXXXXXXXXXXXXXXXXXXXXXXXXXXXXXXXXXX

XXXXXXXXXXXXXXXXXXXXXXXXXXXXXXXXGNVGAPGPKGARGSAGPPGATGFPGAAG

RVGPPGPSGNAGPPGPPGPAGKEGGKGPRGETGPAGRPGEVGPPGPPGPAGEKGSPGADG

PAGAPGTPGPQGIGGQRGVVGLPGQRGERGFPGLPGPSGEPGKQGPSGPNGERGPPGPMG

PPGLAGPPGESGREXXXXXXXXXXXXXXXXXXXXXXXXXXXXXXXXXXXXXXXXXXXXXX

XXXXXXXXGPAGPAGPVGPAGARGPAGPQGPRGDKGETGEQGDRGIKGHRGFSGLQGPPG

PPGSPGEQGPSGASGPAGPRGPPGSAGSPGKDGLNGLPGPIGXXXXXXXXXXXXXXXXXX

XXXXXXXXXXXXXXXXXXXXXXXXXXXXXXXXXXXXXRQYDG-KG-A-GPGPMGLMGPRG

PPGASGPPGPPGFQGPAGEPGEPGQTGPAGSRGPPGPPGKAGEDGHPGKPGRPGERGVVG

PQGARGFPGTPGLPGFKGIRGHNGLDGLKGQPGAPGVKGEPGAPGENGTPGQTGARGLPG

ERGRVGAAGPSGARGSDGSVGPVGPAGPIGAAGPPGFPGAPGPKGELGPVGNPGPTGPAG

PRGEVGLPGVSGPVGPPGNPGANGLAGAKGAAGLPGVAGAPGLPGPRGIPGPVGAAGATG

ARGLVGEPGPPGSKGESGSKGEPGSAGAQGPPGPSGEEGKRGPNGEGGATGPPGPPGLRG

SPGSRGLPGADGRAGVMGPPGSRGASGPAGVRGPSGDAGRPGEPGLMGPRGLPGSPGNVG

PAGKEGPAGLPGIDGRPGPIGPAGARGEPGNIGFPGPKGPTGDPGKAGEKGHAGLAGPRG

APGPDGNNGAQGPPGPQGVQGGKGEQGPAGPPGFQGLPGPAGPAGEAGKPGERXXXXXXX

XXXXXXXXGERGPPGQSGAAGPTGPIGSRGPSGPPGPDGNKGEPGVVGAPGTAGPSGPSG

LPGERGAAGIPGGKGEKGETGLRGDAGNTGRDGARGAPGAVGAPGPAGATGDRGEAGPAG

PAGPAGPRGSPGERGEVGPAGPNGFAGPAGAAGQPGAKGERGTKGPKGENGPVGPPGPVG

AAGPAGPNGPPGPAGGRGDGGPPGATGFPGAAGRTGPPGPSXXXXXXXXXXXXXXXXXXX

XXXXXGPSGRTGETGASGPPGFAGEKGPPGEPGTAGPPGSPGPQGLLGAPGILGLPGSRG

ERGLPGVAGAVGEPGPLGIAGPAGARGPPGNVGSPGVNGAPGEAGRDGNPGNDGPPGRDG

LPGHKGERGYPGNIGPVGAAGAPGPQGAVGPAGKHGNRGEPGPVGSVGPVGPVGPRGPSG

TQGIRGDKGEPGDKGPRGLPGLKGHNGLQGLPGLAGQHGDQGAPGSVGPAGPRGPAGPTG

PAGKDGRSGHPGPVGPAGVRGSQGSQGPSGPPGPPGPPGPPGASGGGYDLGYDGDFYRA

>DASYPUS

QFSYGYDEKSAGGVSVPGPMGPSGPRGLPGPPGSPGPQGFQGPPGEPGEPGSSGPMGPRG

PPGPPGKNGDDGEAGKPGRPGERGPPGPQGARGLPGTAGLPGMKGHRGFSGLDGAKGDAG

PAGPKGEPGSPGENGAPGQMXXXXXXXXXXXXXXXXXXXXXXXXXXXXXXXXXXXXXXXX

XXXXXXXXXXXGEAGPQGARGSEGPQGVRGEPGPPGPAGAAGPAGNPGADGQPGAKGANG

APGIAGAPGFPGARGPSGPQGPSGAPGPKGNSGEPGAPGNKGDTGAKGEPGPTGIQGPPG

PAGEEGKRGARGEPGPTGLPGAPGERGGPGSRGFPGADGIAGPKGPAGERGSPGPAGPKG

SPGEAGRPGEAGLPGAKGLTGSPGSPGPDGKTGPPGPAGQDGRPGPAGPPGARGQAGVMG

FPGPKGAAXXXXXXXXXXXXXXXXXXXXXXXXXXXXXXXXXXXXXXXXXXXXXXXXXXXX

XXGLPGPAGPPGEAGKPGEQGVPGDLGAPGPSGARGERGFPGERGVQGPPGPAGPRGANG

APGNDGAKGDAGAPGAPGSQGAPGLQGMPGERGAAGLPGPKGDRXXXXXXXXXXXXXXXX

XXXXXXXXXXXXXXXXXXXXGETGPSGPAGPTGARGAPGDRGEPGPPGPAGFAGPPGADG

QPGAKGEPGDAGAKGDAGPPGPAGAAGPPGPIGNVGAPGPKGARGSAGPPGATGFPGAAG

RVGPPGPSGNAGPPGPPGPVGKEGGKGPRGETGPAGRPGEVGPPGPPGPSGEKGSPGADG

PAGAPGTPGPQGIAGQRGVVGLPGQRGERGFPGLPGPSGEPGKQGPSGSSGERGPPGPMG

PPGLAGPPGEAGREGSPGAEGSPGRDGSPGPKGDRGETGPAGPPGAPGAPGAPGPVGPAG

KSGDRGETGPSGPAGPAGPAGARGPSGPQGPRGDKGETGEQGDRGIKGHRGFSGLQGPAG

PPXXXXXXXXXXXXXXXXXXGPPGSAGTPGKDGLNGLPGPIGPPGPRGRTGDAGPVGPPG

PPGPPGPPGPPSGGFDFSFLPQPPQEKGHDGGRYYRARQYDG-KGVGLGPGPMGLMGPRG

PPGASGAPGPQGFQGPAGEPGEPGQTGPAGARGPAGPPGKAGEDGHPGKPGRPGERGVVG

PQGARGFPGTPGLPGFKGIRGHNGLDGLKGQAGAPGVKGEPGAPGENGTPGQTGARGLPG

ERGRVGAPGPAGARGSDGSVGXXGPAGPIGSAGPPGFPGAPGPKGELGPVGNPGPAGPAG

PRGEQGLPGVSGPVGPPXXXXXXXXXXXXXXXGLPGVAGAPGLPGPRGIPGPVGAVGATG

ARGLVGEPGPAGSKGESGNKGEPGSAGPQGPPGPSGEEGKRGANGEAGSTGPSGPPGLRG

GPGSRGLPGADGRAGVMGPAGSRGASGPAGVRGPNGDPGRPGEPGLMGPRGLPGSPGNVG

PAGKEGPVGLPGIDGRPGPVGPAGPRGEAGNIGFPGPKGPTGDPGKVGEKGHAGLAGNRG

APGPDGNNGAQGPPGLQGVQGGKGEQGPAGPPGFQGLPGPAGTTGEVGKPGERGLHGEFG

LPGPAGPRGERGPPGESGAAGPVGSIGSRGPSGPPGPDGNKGEPGVVGAPGTAGPSGSGG

LPGERGGAGIPGGKGEKGETGLRGEVGTTGRDGARGAPGAIGAPGPAGATGDRGEAGAAG

PAGPSGPRGTPGERGEVGPAGPNGFAGPAGAAGQPGAKGERGTKGPKGENGIAGPTGPVG

AAGPSGPNGAPGPAGGRGDGGPPGVTGFPGAAGRTGPPGPSGITGPPGPPGAAGKEGLRG

PRGDQGPVGRTGETGAGGPPGFAGEKGPSGEPGTAGPPGTAGPQGLLGAPGILGLPGSRG

ERGLPGVAGAVGEPGPLGISGPPGARGPSGAVGSPGVNGAPGETGRDGNPGNDGPPGRDG

LPGHKGERGYAGNAGPVGAAGAPGPHGSVGPAGKHGNRGEPGPVGPVGPVGAVGPRGPSG

PQGVRGDKGEPGEKGPRGLPGLKGHNGLQGLPGLAGQHGDQGSPGPVGPAGPRGPAGPSG

PAGKDGRTGHPGAVGPAGIRGSQGSQGPSGPAGPPGPPGPPGASGGGYDFGYEGDFYRA

>MUSTELA

QMSYGYDEKSTGGISVPGPMGPSGPRGLPGPPGAPGPQGFQGPPGEPGEPGASGPMGPRG

PPGPPGKNGDDGEAGKPGRPGERGPPGPQGARGLPGTAGLPGMKGHRGFSGLDGAKGDAG

PAGPKGEPGSPGENGAPGQMGPRGLPGERGRPGAPGPAGARGNDGATGAAGPPGPTGPAG

PPGFPGAVGAKGEAGPQGARGSEGPQGVRGEPGPPGPAGAAGPAGNPGADGQPGAKGANG

APGIAGAPGFPGARGPSGPQGPSGPPGPKGNSGEPGAPGNKGDTGAKGEPGPTGIQGPPG

PAGEEGKRGARGEPGPTGLPGPPGERGGPGSRGFPGADGVAGPKGPAGERGSPGPAGPKG

SPGEAGRPGEAGLPGAKGLTGSPGSPGPDGKTGPPGPAGQDGRPGPPGPPGARGQAGVMG

FPGPKGAAGEPGKAGERGVPGPPGAVGPAGKDGEAGAQGAPGPAGPAGERGEQGPAGSPG

FQGLPGPAGPPGEAGKPGEQGVPGDLGAPGPSGARGERGFPGERGVQGPPGPAGPRGANG

APGNDGAKGDAGAPGAPGSQGAPGLQGMPGERGAAGLPGPKGDRGDAGPKGADGSPGKDG

VRGLTGPIGPPGPAGAPGDKGEAGPSGPAGPTGARGAPGDRGEPGPPGPAGFAGPPGADG

QPGAKGEPGDAGAKGDAGPPGPAGPTGPPGPIGNVGAPGPKGARGSAGPPGATGFPGAAG

RVGPPGPSGNAGPPGPPGPAGKEGGKGPRGETGPAGRPGEVGPPGPPGPAGEKGSPGADG

PAGAPGTPGPQGIAGQRGVVGLPGQRGERGFPGLPGPSGEPGKQGPSGASGERGPPGPMG

PPGLAGPPGESGREGSPGAEGSPGRDGSPGPKGDRGETGPAGPPGAPGAPGAPGPVGPAG

KSGDRGETGPAGPAGPIGPVGARGPTGPQGPRGDKGETGEQGDRGIKGHRGFSGLQGPPG

PPGSPGEQGPSGASGPAGPRGPPGSAGSPGKDGLNGLPGPIGPPGPRGRTGDAGPVGPPG

PPGPPGPPGPPSGGFDFSFLPQPPQEKAHDGGRYYRARQYDG-KGVGLGPGPMGLMGPRG

PPGASGAPGPQGFQGPAGEPGEPGQTGPAGARGPPGPPGKAGEDGHPGKPGRPGERGVVG

PQGARGFPGTPGLPGFKGIRGHNGLDGLKGQPGAPGVKGEPGAPGENGTPGQTGARGLPG

ERGRVGAPGPAGARGSDGSVGPVGPAGPIGSAGPPGFPGAPGPKGELGPVGNPGPAGPAG

PRGEVGLPGVSGPVGPPGNPGANGLTGAKGAAGLPGVAGAPGLPGPRGIPGPVGAAGATG

ARGLVGEPGPAGSKGESGNKGEPGSAGPQGPPGPSGEEGKRGPNGEAGSAGPSGPPGLRG

SPGSRGLPGADGRAGVMGPPGPRGATGPAGVRGPNGDSGRPGEPGLMGPRGFPGAPGNIG

PAGKEGPMGLPGIDGRPGPIGPAGARGEPGNIGFPGPKGPTGDPGKPGEKGHAGLAGARG

APGPDGNNGAQGPPGPQGVQGGKGEQGPAGPPGFQGLPGPAGTAGEVGKPGERGLPGEFG

LPGPAGPRGERGPPGESGAAGPSGPIGSRGPSGPPGPDGNKGEPGVLGAPGTAGPSGPGG

LPGERGAAGVPGGKGEKGETGLRGEVGNPGRDGARGAPGAVGAPGPAGATGDRGEAGPAG

PAGPAGPRGSPGERGEVGPAGPNGFAGPAGAAGQPGAKGERGTKGPKGENGPVGPTGPVG

SAGPSGPNGPPGPAGSRGDGGPPGATGFPGAAGRTGPPGPSGITGPPGPPGAAGKEGLRG

PRGDQGPVGRTGETGAHGPPGFAGEKGPSGEPGTAGPPGTPGPQGLLGAPGILGLPGSRG

ERGLPGVSGSVGEPGPLGIAGPPGARGPPGAVGAPGVNGAPGEAGRDGNPGNDGPPGRDG

QPGHKGERGYPGNIGPVGAVGAPGPHGPVGPTGKHGNRGEPGPAGSVGPVGAAGPRGPSG

PQGVRGDKGEPGDKGPRGLPGLKGHNGLQGLPGLAGQHGDQGAPGSVGPAGPRGPAGPSG

PAGKDGRTGHPGTVGPAGIRGSQGSQGPAGPPGPPGPPGPPGPSGGGYDFGYEGDFYRA

>PAN

QLSYGYDEKSTGGISVPGPMGPSGPRGLPGPPGAPGPQGFQGPPGEPGEPGASGPMGPRG

PPGPPGKNGDDGEAGKPGRPGERGPPGPQGARGLPGTAGLPGMKGHRGFSGLDGAKGDAG

PAGPKGEPGSPGENGAPGQMGPRGLPGERGRPGAPGPAGARGNDGATGAAGPPGPTGPAG

PPGFPGAVGAKGEAGPQGPRGSEGPQGVRGEPGPPGPAGAAGPAGNPGADGQPGAKGANG

APGIAGAPGFPGARGPSGPQGPGGPPGPKGNSGEPGAPGSKGDTGAKGEPGPVGVQGPPG

PAGEEGKRGARGEPGPTGLPGPPGERGGPGSRGFPGADGVAGPKGPAGERGSPGPAGPKG

SPGEAGRPGEAGLPGAKGLTGSPGSPGPDGKTGPPGPAGQDGRPGPPGPPGARGQAGVMG

FPGPKGAAGEPGKAGERGVPGPPGAVGPAGKDGEAGAQGPPGPAGPAGERGEQGPAGSPG

FQGLPGPAGPPGEAGKPGEQGVPGDLGAPGPSGARGERGFPGERGVQGPPGPAGPRGANG

APGNDGAKGDAGAPGAPGSQGAPGLQGMPGERGAAGLPGPKGDRGDAGPKGADGSPGKDG

VRGLTGPIGPPGPAGAPGDKGESGPSGPAGPTGARGAPGDRGEPGPPGPAGFAGPPGADG

QPGAKGEPGDAGAKGDAGPPGPAGPAGPPGPIGNVGAPGAKGARGSAGPPGATGFPGAAG

RVGPPGPSGNAGPPGPPGPAGKEGGKGPRGETGPAGRPGEVGPPGPPGPAGEKGSPGADG

PAGAPGTPGPQGIAGQRGVVGLPGQRGERGFPGLPGPSGEPGKQGPSGASGERGPPGPMG

PPGLAGPPGESGREGAPGAEGSPGRDGSPGAKGDRGETGPAGPPGAPGAPGAPGPVGPAG

KSGDRGETGPAGPAGPVGPVGARGPAGPQGPRGDKGETGEQGDRGIKGHRGFSGLQGPPG

PPGSPGEQGPSGASGPAGPRGPPGSAGAPGKDGLNGLPGPIGPPGPRGRTGDAGPVGPPG

PPGPPGPPGPPSAGFDFSFLPQPPQEKAHDGGRYYRARQYDG-KGVGLGPGPMGLMGPRG

PPGAAGAPGPQGFQGPAGEPGEPGQTGPAGARGPAGPPGKAGEDGHPGKPGRPGERGVVG

PQGARGFPGTPGLPGFKGIRGHNGLDGLKGQPGAPGVKGEPGAPGENGTPGQTGARGLPG

ERGRVGAPGPAGARGSDGSVGPVGPAGPIGSAGPPGFPGAPGPKGELGAVGNAGPAGPAG

PRGEVGLPGLSGPVGPPGNPGANGLTGAKGAAGLPGVAGAPGLPGPRGIPGPVGAAGATG

ARGLVGEPGPAGSKGESGNKGEPGSAGPQGPPGPSGEEGKRGPNGEAGSAGPPGPPGLRG

SPGSRGLPGADGRAGVMGPAGSRGASGPAGVRGPNGDAGRPGEPGLMGPRGLPGSPGNIG

PAGKEGPVGLPGIDGRPGPIGPAGARGEPGNIGFPGPKGPTGDPGKNGDKGHAGLAGARG

APGPDGNNGAQGPPGPQGVQGGKGEQGPAGPPGFQGLPGPSGPTGEVGKPGERGLHGEFG

LPGPAGPRGERGPPGESGAAGPTGPIGSRGPSGPPGPDGNKGEPGVVGAVGTAGPSGPSG

LPGERGAAGIPGGKGEKGEPGLRGEIGNPGRDGARGAPGAVGAPGPAGATGDRGEAGAAG

PAGPAGPRGSPGERGEVGPAGPNGFAGPAGAAGQPGAKGERGAKGPKGENGVVGPTGPVG

AAGPAGPNGPPGPAGSRGDGGPPGMTGFPGAAGRTGPPGPSGISGPPGPPGPAGKEGLRG

PRGDQGPVGRTGEVGAVGPPGFAGEKGPSGEAGTAGPPGTPGPQGLLGAPGILGLPGSRG

ERGLPGVAGAVGEPGPLGIAGPPGARGPPGAVGSPGVNGAPGEAGRDGNPGNDGPPGRDG

QPGHKGERGYPGNIGPVGAAGAPGPHGPVGPAGKHGNRGETGPSGPVGPAGAVGPRGPSG

PQGIRGDKGEPGEKGPRGLPGLKGHNGLQGLPGLAGHHGDQGAPGSVGPAGPRGPAGPSG

PAGKDGRTGHPGTVGPAGIRGPQGHQGPAGPPGPPGPPGPPGVSGGGYDFGYDGDFYRA

>NOMASCUS

QLSYGYDEKSAGGISVPGPMGPSGPRGLPGPPGAPGPQGFQGPPGEPGEPGASGPMGPRG

PPGPPGKNGDDGEAGKPGRPGERGPPGPQGARGLPGTAGLPGMKGHRGFSGLDGAKGDAG

PAGPKGEPGSPGENGAPGQMGPRGLPGERGRPGAPGPAGARGNDGATGAAGPPGPTGPAG

PPGFPGAVGAKGEAGPQGPRGSEGPQGVRGEPGPPGPAGAAGPAGNPGADGQPGAKGANG

APGIAGAPGFPGARGPSGPQGPGGPPGPKGNSGEPGAPGSKGDTGAKGEPGPVGVQGPPG

PAGEEGKRGARGEPGPTGLPGPPGERGGPGSRGFPGADGVAGPKGPAGERGSPGPAGPKG

SPGEAGRPGEAGLPGAKGLTGSPGSPGPDGKTGPPGPAGQDGRPGPPGPPGARGQAGVMG

FPGPKGAAGEPGKAGERGVPGPPGAVGPAGKDGEAGAQGPPGPAGPAGERGEQGPAGSPG

FQGLPGPAGPPGEAGKPGEQGVPGDLGAPGPSGARGERGFPGERGVQGPPGPAGPRGANG

APGNDGAKGDAGAPGAPGSQGAPGLQGMPGERGAAGLPGPKGDRGDAGPKGADGSPGKDG

VRGLTGPIGPPGPAGAPGDKGETGPSGPAGPTGARGAPGDRGEPGPPGPAGFAGPPGADG

QPGAKGEPGDAGAKGDAGPPGPAGPAGPPGPIGNVGAPGAKGARGSAGPPGATGFPGAAG

RVGPPGPSGNAGPPGPPGPAGKEGGKGPRGETGPAGRPGEVGPPGPPGPAGEKGSPGADG

PAGAPGTPGPQGIAGQRGVVGLPGQRGERGFPGLPGPSGEPGKQGPSGASGERGPPGPMG

PPGLAGPPGESGREGAPGAEGSPGRDGSPGPKGDRGETGPAGPPGAPGAPGAPGPVGPAG

KSGDRGETGPAGPAGPVGPVGARGPAGPQGPRGDKGETGEQGDRGIKGHRGFSGLQGPPG

PPGSPGEQGPSGASGPAGPRGPPGSAGAPGKDGLNGLPGPIGPPGPRGRTGDAGPVGPPG

PPGPPGPPGPPSAGFDFSFLPQPPQEKAHDGGRYYRARQYDG-KGVGLGPGPMGLMGPRG

PPGAAGAPGPQGFQGPAGEPGEPGQTGPAGARGPAGPPGKAGEDGHPGKPGRPGERGVVG

PQGARGFPGTPGLPGFKGIRGHNGLDGLKGQPGAPGVKGEPGAPGENGTPGQTGARGLPG

ERGRVGAPGPAGARGSDGSVGPVGPAGPIGSAGPPGFPGAPGPKGELGAVGNAGPAGPAG

PRGEVGLPGLSGPVGPPGNPGANGLTGAKGAAGLPGVAGAPGLPGPRGIPGPVGAAGATG

ARGLVGEPGPAGSKGESGNKGEPGSAGPQGPPGPSGEEGKRGPNGEAGSAGPPGPPGLRG

SPGSRGLPGADGRAGVMGPPGSRGASGPAGVRGPNGDAGRPGEPGLMGPRGLPGSPGNIG

PAGKEGPVGLPGIDGRPGPIGPAGARGEAGNIGFPGPKGPTGDPGKSGDKGHAGLAGARG

APGPDGNNGAQGPPGPQGVQGGKGEQGPAGPPGFQGLPGPSGPAGEVGKPGERGLHGEFG

LPGPAGPRGERGPPGESGAAGPTGPIGSRGPSGPPGPDGNKGEPGVVGAVGTAGPSGPSG

LPGERGAAGIPGGKGEKGEPGLRGEIGNPGRDGARGAPGAVGAPGPAGATGDRGEAGAAG

PAGPAGPRGSPGERGEVGPAGPNGFAGPAGAAGQPGAKGERGAKGPKGENGVVGPTGPVG

AAGPAGPNGPPGPAGSRGDGGPPGMTGFPGAAGRTGPPGPSGISGPPGPPGPAGKEGLRG

PRGDQGPVGRTGEVGAVGPPGFAGEKGPSGEAGTAGPPGTPGPQGLLGAPGILGLPGSRG

ERGLPGVAGAVGEPGPLGIAGPPGARGPPGAVGSPGVNGAPGEAGRDGNPGNDGPPGRDG

QPGHKGERGYPGNIGPVGAAGAPGPHGPVGPAGKHGNRGETGPSGPVGPAGAVGPRGPSG

PQGIRGDKGEPGDKGPRGLPGLKGHNGLQGLPGLAGHHGDQGAPGSVGPAGPRGPAGPSG

PAGKDGRTGHPGTVGPAGIRGPQGHQGPAGPPGPPGPPGPPGVSGGGYDFGYDGDFYRA

>MACACA

QLSYGYDEKSTGGISVPGPMGPSGPRGLPGPPGAPGPQGFQGPPGEPGEPGASGPMGPRG

PPGPPGKNGDDGEAGKPGRPGERGPPGPQGARGLPGTAGLPGMKGHRGFSGLDGAKGDAG

PAGPKGEPGSPGENGAPGQMGPRGLPGERGRPGAPGPAGARGNDGATGAAGPPGPTGPAG

PPGFPGAVGAKGEAGPQGPRGSEGPQGVRGEPGPPGPAGAAGPAGNPGADGQPGAKGANG

APGIAGAPGFPGARGPSGPQGPGGPPGPKGNSGEPGAPGSKGDTGAKGEPGPVGVQGPPG

PAGEEGKRGARGEPGPTGLPGPPGERGGPGSRGFPGADGVAGPKGPAGERGSPGPAGPKG

SPGEAGRPGEAGLPGAKGLTGSPGSPGPDGKTGPPGPAGQDGRPGPPGPPGARGQAGVMG

FPGPKGAAGEPGKAGERGVPGPPGAVGPAGKDGEAGAQGPPGPAGPAGERGEQGPAGSPG

FQGLPGPAGPPGEAGKPGEQGVPGDLGAPGPSGARGERGFPGERGVQGPPGPAGPRGANG

APGNDGAKGDAGAPGAPGSQGAPGLQGMPGERGAAGLPGPKGDRGDAGPKGADGSPGKDG

VRGLTGPIGPPGPAGAPGDKGETGPSGPAGPTGARGAPGDRGEPGPPGPAGFAGPPGADG

QPGAKGEPGDAGAKGDAGPPGPAGPAGPPGPIGNVGAPGPKGARGSAGPPGATGFPGAAG

RVGPPGPSGNAGPPGPPGPAGKEGGKGPRGETGPAGRPGEVGPPGPPGPAGEKGSPGADG

PAGAPGTPGPQGIAGQRGVVGLPGQRGERGFPGLPGPSGEPGKQGPSGASGERGPPGPMG

PPGLAGPPGESGREGAPGAEGSPGRDGSPGAKGDRGETGPAGPPGAPGAPGAPGPVGPAG

KSGDRGETGPAGPAGPVGPVGARGPAGPQGPRGDKGETGEQGDRGIKGHRGFSGLQGPPG

PPGSPGEQGPSGASGPAGPRGPPGSAGTPGKDGLNGLPGPIGPPGPRGRTGDAGPVGPPG

PPGPPGPPGPPSGGFDFSFLPQPPQEKAHDGGRYYRARQYDG-KGVGLGPGPMGLMGPRG

PPGAAGAPGPQGFQGPAGEPGEPGQTGPAGSRGPAGPPGKAGEDGHPGKPGRPGERGVVG

PQGARGFPGTPGLPGFKGIRGHNGLDGLKGQPGAPGVKGEPGAPGENGTPGQTGARGLPG

ERGRVGAPGPAGARGSDGSVGPVGPAGPIGSAGPPGFPGAPGPKGELGAVGNAGPAGPAG

PRGEVGLPGLSGPVGPPGNPGANGLTGAKGAAGLPGVAGAPGLPGPRGIPGPVGAAGATG

ARGLVGEPGPAGSKGESGNKGEPGSAGPQGPPGPSGEEGKRGPNGEVGSAGPPGPPGLRG

SPGSRGLPGADGRAGVMGPPGSRGASGPAGVRGPNGDAGRPGEPGLMGPRGLPGSPGNIG

PAGKEGPVGLPGIDGRPGPIGPAGARGEPGNIGFPGPKGPTGDPGKNGDKGHAGLAGARG

APGPDGNNGAQGPPGPQGVQGGKGEQGPAGPPGFQGLPGPSGPAGEVGKPGERGLPGEFG

LPGPAGARGERGPPGESGAAGPTGPIGSRGPSGPPGPDGNKGEPGVVGAAGTAGPSGPSG

LPGERGAAGIPGGKGEKGEPGLRGEIGNPGRDGARGAPGAVGAPGPAGATGDRGEAGAAG

PAGPAGPRGSPGERGEVGPAGPNGFAGPAGAAGQPGAKGERGAKGPKGENGVVGPTGPVG

AAGPSGPNGPPGPAGSRGDGGPPGMTGFPGAAGRTGPPGPSGISGPPGPPGPSGKEGLRG

PRGDQGPVGRTGEVGAVGPPGFAGEKGPSGEAGTAGPPGTPGPQGLLGAPGILGLPGSRG

ERGLPGVAGVVGEPGPLGIAGPPGARGPPGAVGSPGVNGAPGEAGRDGNPGNDGPPGRDG

QPGHKGERGYPGNNGPVGAAGAPGPHGPVGPAGKHGNRGETGPSGPVGPAGAVGPRGPSG

PQGIRGDKGEPGDKGPRGLPGLKGHNGLQGLPGLAGHHGDQGAPGSVGPAGPRGPAGPSG

PAGKDGRTGHPGTVGPAGIRGPQGHQGPAGPPGPPGPPGPPGVSGGGYDFGYDGDFYRA

>OTOLEMUR

QMSYGYDEKSAG-VSVPGPMGPSGPRGLPGPPGAPGPQGFQGPPGEPGEPGSAGPMGPRG

PPGPPGKNGDDGEAGKPGRPGERGPPGPQGARGLPGTAGLPGMKGHRGFSGLDGAKGDAG

APGPKGEPGSPGENGAPGQMGPRGLPGERGRPGPSGPAGARGNDGATGAAGPPGPTGPAG

PPGFPGAAGAKGEAGPQGARGSEGPQGVRGEPGPPGPAGAAGPAGNPGADGQPGAKGANG

APGIAGAPGFPGARGPSGPQGPSGPPGPKGNSGEPGAPGNKGDTGAKGEPGPAGVQGPPG

PAGEEGKRGARGEPGPTGLPGPPGERGGPGSRGFPGADGVAGPKGPAGERGSPGPAGPKG

SPGEAGRPGEAGLPGAKGLTGSPGSPGPDGKTGPPGPAGQDGRPGPPGPPGARGQAGVMG

FPGPKGAAGEPGKAGERGVPGPTGAVGAPGKDGEAGAQGPPGPAGPAGERGEQGPAGSPG

FQGLPGPAGPPGEAGKPGEQGVPGDLGAPGPSGARGERGFPGERGVQGPPGPAGPRGGNG

APGNDGAKGDAGAPGAPGSQGAPGLQGMPGERGAAGLPGPKGDRGDAGPKGADGSPGKDG

ARGLTGPIGPPGPAGAPGDKGESGPSGPAGPTGARGAPGDRGEPGPPGPAGFAGPPGADG

QPGAKGEPGDAGAKGDAGPAGPAGPAGPPGPVGNVGAPGPKGARGSAGPPGATGFPGAAG

RVGPPGPSGNAGPPGPPGPAGKEGSKGPRGETGPAGRTGEVGPPGPPGPAGEKGSPGADG

PAGAPGTPGPQGIAGQRGVVGLPGQRGERGFPGLPGPSGEPGKQGPSGASGERGPPGPMG

PPGLAGPPGESGREGAPGAEGSPGRDGAPGPKGDRGETGPAGPPGAPGAPGAPGPVGPAG

KSGDRGETGPSGPAGPVGPAGARGPAGPQGPRGDKGETGEQGERGIKGHRGFSGLQGPPG

PPGSPGEQGPSGASGPAGPRGPPGSAGAAGKDGLNGLPGPIGPPGPRGRTGDAGPVGPAG

PPGPPGPPGPPSGGFDFSFLPQPPQEKAQDSGRYYRARQYDG-KAAGLGPGPMGLMGPRG

PPGASGAPGPQGFQGPAGEPGEPGQTGPAGARGPAGAPGKAGEDGHPGKPGRPGERGVVG

PQGARGFPGTPGLPGFKGIRGHSGPDGLKGQAGLPGAKGEPGSPGENGTPGQTGARGLPG

ERGRVGAPGPSGARGSDGSVGPVGPAGPVGSAGPPGFPGAPGPKGELGPVGNPGPAGPAG

PRGEVGLPGLSGPVGPPGNPGANGLTGAKGAAGLPGVAGAPGLPGPRGIPGPVGAAGATG

ARGLVGEPGPAGSKGESGNKGEPGSAGPQGPPGPSGEEGKRGSNGEPGSAGPSGPPGLRG

SPGSRGLPGADGRGGVMGPPGNRGQSGPAGVRGPSGDSGRPGEPGLMGPRGLPGSPGNVG

PAGKEGPAGLPGVDGRPGPVGPAGARGEPGNIGFPGPKGPSGDPGKAGDKGHPGLAGARG

APGPDGNNGAQGPPGPQGVQGGKGEQGPAGPPGFQGLPGPSGPAGEVGKPGERGLHGEFG

LPGPAGPRGERGPPGESGAAGPSGPIGSRGPSGPPGPDGNKGEPGVVGAPGTAGPSGPSG

LPGERGAAGMPGGKGEKGETGPRGEMGTTGRDGARGAPGAVGAPGPAGATGDRGEAGAAG

PAGPAGPRGSPGERGEVGPAGPNGFAGPAGAAGQPGAKGERGAKGPKGENGAVGPAGAVG

PAGPSGPNGPPGPAGGRGDGGPPGMTGFPGAAGRTGPPGPSGMSGPPGPPGPSGKEGLRG

PRGDQGPVGRSGETGPSGPPGFAGEKGPSGEAGAAGPPGTPGPQGLLGAPGILGLPGSRG

ERGLPGVAGAVGEPGPLGVAGPPGARGPSGGVGNPGVNGAPGEAGRDGNPGNDGPPGRDG

QPGHKGERGYPGNVGPAGAVGAPGSHGPVGPAGKHGNRGEPGAVGPVGPTGAVGPRGPSG

AQGVRGDKGEPGDKGPRGLPGLKGHGGLQGLPGLAGHHGDQGAPGSVGPAGPRGPAGPSG

PVGKDGRNGHPGTVGPAGVRGPQGHQGPAGPPGPPGPPGPPGASGGGYDFGYDGDFYRA

>RATTUS

QMSYGYDEKSAG-VSVPGPMGPSGPRGLPGPPGAPGPQGFQGPPGEPGEPGASGPMGPRG

PPGPPGKNGDDGEAGKPGRPGERGPPGPQGARGLPGTAGLPGMKGHRGFSGLDGAKGDTG

PAGPKGEPGSPGENGAPGQMGPRGLPGERGRPGPPGSAGARGNDGAVGAAGPPGPTGPTG

PPGFPGAAGAKGEAGPQGARGSEGPQGVRGEPGPPGPAGAAGPAGNPGADGQPGAKGANG

APGIAGAPGFPGARGPSGPQGPSGAPGPKGNSGEPGAPGNKGDTGAKGEPGPAGVQGPPG

PAGEEGKRGARGEPGPSGLPGPPGERGGPGSRGFPGADGVAGPKGPAGERGSPGPAGPKG

SPGEAGRPGEAGLPGAKGLTGSPGSPGPDGKTGPPGPAGQDGRPGPAGPPGARGQAGVMG

FPGPKGTAGEPGKAGERGVPGPPGAVGPAGKDGEAGAQGAPGPAGPAGERGEQGPAGSPG

FQGLPGPAGPPGEAGKPGEQGVPGDLGAPGPSGARGERGFPGERGVQGPPGPAGPRGNNG

APGNDGAKGDTGAPGAPGSQGAPGLQGMPGERGAAGLPGPKGDRGDAGPKGADGSPGKDG

VRGLTGPIGPPGPAGAPGDKGETGPSGPAGPTGARGAPGDRGEPGPPGPAGFAGPPGADG

QPGAKGEPGDTGVKGDAGPPGPAGPAGPPGPIGNVGAPGPKGSRGAAGPPGATGFPGAAG

RVGPPGPSGNAGPPGPPGPVGKEGGKGPRGETGPAGRPGEVGPPGPPGPAGEKGSPGADG

PAGSPGTPGPQGIAGQRGVVGLPGQRGERGFPGLPGPSGEPGKQGPSGASGERGPPGPMG

PPGLAGPPGESGREGSPGAEGSPGRDGAPGAKGDRGETGPAGPPGAPGAPGAPGPVGPAG

KNGDRGETGPAGPAGPIGPAGARGPAGPQGPRGDKGETGEQGDRGIKGHRGFSGLQGPPG

SPGSPGEQGPSGASGPAGPRGPPGSAGSPGKDGLNGLPGPIGPPGPRGRTGDSGPAGPPG

PPGPPGPPGPPSGGYDFSFLPQPPQEKSQDGGRYYRARQYSD-KGVSAGPGPMGLMGPRG

PPGAVGAPGPQGFQGPAGEPGEPGQTGPAGSRGPAGPPGKAGEDGHPGKPGRPGERGVVG

PQGARGFPGTPGLPGFKGIRGHNGLDGLKGQPGAQGVKGEPGAPGENGTPGQAGARGLPG

ERGRVGAPGPAGARGSDGSVGPVGPAGPIGSAGPPGFPGAPGPKGELGPVGNPGPAGPAG

PRGEAGLPGLSGPVGPPGNPGANGLTGAKGATGLPGVAGAPGLPGPRGIPGPVGAAGATG

PRGLVGEPGPAGSKGETGNKGEPGSAGAQGPPGPSGEEGKRGSPGEPGSAGPAGPPGLRG

SPGSRGLPGADGRAGVMGPPGNRGSTGPAGVRGPNGDAGRPGEPGLMGPRGLPGSPGNVG

PAGKEGPVGLPGIDGRPGPIGPAGPRGEAGNIGFPGPKGPSGDPGKPGEKGHPGLAGARG

APGPDGNNGAQGPPGPQGVQGGKGEQGPAGPPGFQGLPGPSGTAGEVGKPGERGLPGEFG

LPGPAGPRGERGPPGESGAAGPSGPIGIRGPSGAPGPDGNKGEAGAVGAPGSAGASGPGG

LPGERGAAGIPGGKGEKGETGLRGEIGNPGRDGARGAPGAIGAPGPAGASGDRGEAGAAG

PSGPAGPRGSPGERGEVGPAGPNGFAGPAGSAGQPGAKGEKGTKGPKGENGIVGPTGPVG

AAGPSGPNGPPGPAGSRGDGGPPGMTGFPGAAGRTGPPGPSGITGPPGPPGAAGKEGLRG

PRGDQGPVGRTGEIGASGPPGFAGEKGPSGEPGTTGPPGTAGPQGLLGAPGILGLPGSRG

ERGQPGIAGALGEPGPLGIAGPPGARGPPGAVGSPGVNGAPGEAGRDGNPGSDGPPGRDG

QPGHKGERGYPGNIGPTGAAGAPGPHGSVGPAGKHGNRGEPGPAGSVGPVGAVGPRGPSG

PQGIRGDKGEPGDKGARGLPGLKGHNGLQGLPGLAGLHGDQGAPGPVGPAGPRGPAGPSG

PIGKDGRSGHPGPVGPAGVRGSQGSQGPAGPPGPPGPPGPPGVSGGGYDFGFEGGFYRA

>MUS

QMSYGYDEKSAG-VSVPGPMGPSGPRGLPGPPGAPGPQGFQGPPGEPGEPGGSGPMGPRG

PPGPPGKNGDDGEAGKPGRPGERGPPGPQGARGLPGTAGLPGMKGHRGFSGLDGAKGDAG

PAGPKGEPGSPGENGAPGQMGPRGLPGERGRPGPPGTAGARGNDGAVGAAGPPGPTGPTG

PPGFPGAVGAKGEAGPQGARGSEGPQGVRGEPGPPGPAGAAGPAGNPGADGQPGAKGANG

APGIAGAPGFPGARGPSGPQGPSGPPGPKGNSGEPGAPGNKGDTGAKGEPGATGVQGPPG

PAGEEGKRGARGEPGPSGLPGPPGERGGPGSRGFPGADGVAGPKGPSGERGAPGPAGPKG

SPGEAGRPGEAGLPGAKGLTGSPGSPGPDGKTGPPGPAGQDGRPGPAGPPGARGQAGVMG

FPGPKGTAGEPGKAGERGLPGPPGAVGPAGKDGEAGAQGAPGPAGPAGERGEQGPAGSPG

FQGLPGPAGPPGEAGKPGEQGVPGDLGAPGPSGARGERGFPGERGVQGPPGPAGPRGNNG

APGNDGAKGDTGAPGAPGSQGAPGLQGMPGERGAAGLPGPKGDRGDAGPKGADGSPGKDG

ARGLTGPIGPPGPAGAPGDKGEAGPSGPPGPTGARGAPGDRGEAGPPGPAGFAGPPGADG

QPGAKGEPGDTGVKGDAGPPGPAGPAGPPGPIGNVGAPGPKGPRGAAGPPGATGFPGAAG

RVGPPGPSGNAGPPGPPGPVGKEGGKGPRGETGPAGRPGEVGPPGPPGPAGEKGSPGADG

PAGSPGTPGPQGIAGQRGVVGLPGQRGERGFPGLPGPSGEPGKQGPSGSSGERGPPGPMG

PPGLAGPPGESGREGSPGAEGSPGRDGAPGAKGDRGETGPAGPPGAPGAPGAPGPVGPAG

KNGDRGETGPAGPAGPIGPAGARGPAGPQGPRGDKGETGEQGDRGIKGHRGFSGLQGPPG

SPGSPGEQGPSGASGPAGPRGPPGSAGSPGKDGLNGLPGPIGPPGPRGRTGDSGPAGPPG

PPGPPGPPGPPSGGYDFSFLPQPPQEKSQDGGRYYRARQYSD-KGVSSGPGPMGLMGPRG

PPGAVGAPGPQGFQGPAGEPGEPGQTGPAGPRGPAGSPGKAGEDGHPGKPGRPGERGVVG

PQGARGFPGTPGLPGFKGVRGHSGMDGLKGQPGAQGVKGEPGAPGENGTPGQAGARGLPG

ERGRVGAPGPAGARGSDGSVGPVGPAGPIGSAGPPGFPGAPGPKGELGPVGNPGPAGPAG

PRGEVGLPGLSGPVGPPGNPGTNGLTGAKGATGLPGVAGAPGLPGPRGIPGPAGAAGATG

ARGLVGEPGPAGSKGESGNKGEPGSVGAQGPPGPSGEEGKRGSPGEAGSAGPAGPPGLRG

SPGSRGLPGADGRAGVMGPPGNRGSTGPAGIRGPNGDAGRPGEPGLMGPRGLPGSPGNVG

PSGKEGPVGLPGIDGRPGPIGPAGPRGEAGNIGFPGPKGPSGDPGKPGERGHPGLAGARG

APGPDGNNGAQGPPGPQGVQGGKGEQGPAGPPGFQGLPGPSGTTGEVGKPGERGLPGEFG

LPGPAGPRGERGTPGESGAAGPSGPIGSRGPSGAPGPDGNKGEAGAVGAPGSAGASGPGG

LPGERGAAGIPGGKGEKGETGLRGDTGNTGRDGARGIPGAVGAPGPAGASGDRGEAGAAG

PSGPAGPRGSPGERGEVGPAGPNGFAGPAGAAGQPGAKGEKGTKGPKGENGIVGPTGSVG

AAGPSGPNGPPGPVGSRGDGGPPGMTGFPGAAGRTGPPGPSGIAGPPGPPGAAGKEGLRG

PRGDQGPVGRTGETGASGPPGFVGEKGPSGEPGTAGAPGTAGPQGLLGAPGILGLPGSRG

ERGLPGIAGALGEPGPLGISGPPGARGPPGAVGSPGVNGAPGEAGRDGNPGSDGPPGRDG

QPGHKGERGYPGSIGPTGAAGAPGPHGSVGPAGKHGNRGEPGPAGSVGPVGAVGPRGPSG

PQGIRGDKGEPGDKGHRGLPGLKGYSGLQGLPGLAGLHGDQGAPGPVGPAGPRGPAGPSG

PVGKDGRSGQPGPVGPAGVRGSQGSQGPAGPPGPPGPPGPPGVSGGGYDFGFEGDFYRA

>HOMO

QLSYGYDEKSTGGISVPGPMGPSGPRGLPGPPGAPGPQGFQGPPGEPGEPGASGPMGPRG

PPGPPGKNGDDGEAGKPGRPGERGPPGPQGARGLPGTAGLPGMKGHRGFSGLDGAKGDAG

PAGPKGEPGSPGENGAPGQMGPRGLPGERGRPGAPGPAGARGNDGATGAAGPPGPTGPAG

PPGFPGAVGAKGEAGPQGPRGSEGPQGVRGEPGPPGPAGAAGPAGNPGADGQPGAKGANG

APGIAGAPGFPGARGPSGPQGPGGPPGPKGNSGEPGAPGSKGDTGAKGEPGPVGVQGPPG

PAGEEGKRGARGEPGPTGLPGPPGERGGPGSRGFPGADGVAGPKGPAGERGSPGPAGPKG

SPGEAGRPGEAGLPGAKGLTGSPGSPGPDGKTGPPGPAGQDGRPGPPGPPGARGQAGVMG

FPGPKGAAGEPGKAGERGVPGPPGAVGPAGKDGEAGAQGPPGPAGPAGERGEQGPAGSPG

FQGLPGPAGPPGEAGKPGEQGVPGDLGAPGPSGARGERGFPGERGVQGPPGPAGPRGANG

APGNDGAKGDAGAPGAPGSQGAPGLQGMPGERGAAGLPGPKGDRGDAGPKGADGSPGKDG

VRGLTGPIGPPGPAGAPGDKGESGPSGPAGPTGARGAPGDRGEPGPPGPAGFAGPPGADG

QPGAKGEPGDAGAKGDAGPPGPAGPAGPPGPIGNVGAPGAKGARGSAGPPGATGFPGAAG

RVGPPGPSGNAGPPGPPGPAGKEGGKGPRGETGPAGRPGEVGPPGPPGPAGEKGSPGADG

PAGAPGTPGPQGIAGQRGVVGLPGQRGERGFPGLPGPSGEPGKQGPSGASGERGPPGPMG

PPGLAGPPGESGREGAPGAEGSPGRDGSPGAKGDRGETGPAGPPGAPGAPGAPGPVGPAG

KSGDRGETGPAGPAGPVGPVGARGPAGPQGPRGDKGETGEQGDRGIKGHRGFSGLQGPPG

PPGSPGEQGPSGASGPAGPRGPPGSAGAPGKDGLNGLPGPIGPPGPRGRTGDAGPVGPPG

PPGPPGPPGPPSAGFDFSFLPQPPQEKAHDGGRYYRARQYDG-KGVGLGPGPMGLMGPRG

PPGAAGPPGPQGFQGPAGEPGEPGQTGPAGARGPAGPPGKAGEDGHPGKPGRPGERGVVG

PQGARGFPGTPGLPGFKGIRGHNGLDGLKGQPGAPGVKGEPGAPGENGTPGQTGARGLPG

ERGRVGAPGPAGARGSDGSVGPVGPAGPIGSAGPPGFPGAPGPKGELGAVGNAGPAGPAG

PRGEVGLPGLSGPVGPPGNPGANGLTGAKGAAGLPGVAGAPGLPGPRGIPGPVGAAGATG

ARGLVGEPGPAGSKGESGNKGEPGSAGPQGPPGPSGEEGKRGPNGEAGSAGPPGPPGLRG

SPGSRGLPGADGRAGVMGPPGSRGASGPAGVRGPNGDAGRPGEPGLMGPRGLPGSPGNIG

PAGKEGPVGLPGIDGRPGPIGPAGARGEPGNIGFPGPKGPTGDPGKNGDKGHAGLAGARG

APGPDGNNGAQGPPGPQGVQGGKGEQGPPGPPGFQGLPGPSGPAGEVGKPGERGLHGEFG

LPGPAGPRGERGPPGESGAAGPTGPIGSRGPSGPPGPDGNKGEPGVVGAVGTAGPSGPSG

LPGERGAAGIPGGKGEKGEPGLRGEIGNPGRDGARGAPGAVGAPGPAGATGDRGEAGAAG

PAGPAGPRGSPGERGEVGPAGPNGFAGPAGAAGQPGAKGERGAKGPKGENGVVGPTGPVG

AAGPAGPNGPPGPAGSRGDGGPPGMTGFPGAAGRTGPPGPSGISGPPGPPGPAGKEGLRG

PRGDQGPVGRTGEVGAVGPPGFAGEKGPSGEAGTAGPPGTPGPQGLLGAPGILGLPGSRG

ERGLPGVAGAVGEPGPLGIAGPPGARGPPGAVGSPGVNGAPGEAGRDGNPGNDGPPGRDG

QPGHKGERGYPGNIGPVGAAGAPGPHGPVGPAGKHGNRGETGPSGPVGPAGAVGPRGPSG

PQGIRGDKGEPGEKGPRGLPGLKGHNGLQGLPGLAGHHGDQGAPGSVGPAGPRGPAGPSG

PAGKDGRTGHPGTVGPAGIRGPQGHQGPAGPPGPPGPPGPPGVSGGGYDFGYDGDFYRA

>OCHOTONA

QMSYGYDEKSAG-VSVPGPMGPSGPRGLPGPPGSPGPQGFQGPPGEPGEPGASGPMGPRG

PPGPPGKNGDDGEAGKPGRPGERGPPGPQGARGLTGTAGLPGMKGHRGFSGLDGAKGDAG

PAGPKGEPGSPGENGAPGQMGPRGLPGERGRPGPPGTAGARGNDGATGAAGPPGPTGPAG

PPGFPGAVGAKGEAGPQGARGSEGPQGIRGEPGPPGPAGAAGPAGNPGADGQPGAKGANG

APGIAGAPGFPGARGPSGPQGPSGPPGPKGNSGEPGAPGNKGDTGAKGEPGPAGVQGPPG

PAGEEGKRGARGEPGPAGLPGPPGERGGPGSRGFPGADGVAGPKGPAGERGAPGPAGPKG

SPGEAGRPGEAGLPGAKGLTGSPGSPGPDGKTGPPGPAGQDGRPGPPGPPGARGQAGVMG

FPGPKGAAGEPGKAGERGVPGPPGAVGAPGKDGEAGAQGPPGPAGPAGERGEQGPAGSPG

FQGLPGPAGPPGEAGKPGEQGVPGDLGAPGPSGARGERGFPGERGVQGPPGPAGPRGSNG

APGNDGAKGDAGAPGAPGSQGAPGLQGMPGERGAAGLPGPKGDRGDAGPKGADGSPGKDG

VRGLTGPIGPPGPAGAPGDKGETGPSGPAGPTGARGAPGDRGEPGPPGPAGFAGPPGADG

QPGAKGEPGDAGAKGDAGPPGPAGPAGPPGPIGNVGAPGPKGARGSAGPPGATGFPGAAG

RVGPPGPSGNAGPPGPPGPAGKEGGKGPRGETGPAGRPGEVGPPGPPGPAGEKGSPGADG

PAGAPGTPGPQGITGQRGVVGLPGQRGERGFPGLPGPSGEPGKQGPSGASGERGPPGPMG

PPGLAGPPGESGREGSPGAEGSPGRDGSPGPKGDRGETGPAGPPGAPGAPGAPGPVGPAG

KSGDRGETGPAGPAGPIGPAGARGPAGPQGPRGDKGETGEQGDRGIKGHRGFSGLQGPPG

PPGSPGEQGPSGASGPAGPRGPPGSAGAPGKDGLNGLPGPIGPPGXXXXXXXXXXXXXXX

XXXXXXXXXXXXXXXXXXXXPQPPQEKAHDGGRYYRARXXXX?XX?X?XXXXMGLMGPRG

PPGAAGAPGPQGFQGPAGEPGEPGQTGPAGARGPPGAPGKAGEDGHPGKPGRPGERGIMG

PQGARGFPGTPGLPGFKGIRGHNGLDGLKGQPGAPGVKGEPGAPGENGTPGQTGARGLPG

ERGRVGAPGPAGARGSDGSVGPVGPAGPIGSAGPPGFPGAPGPKGELGPVGNPGPSGPAG

PRGEVGLPGVSGPVGPPGNPGTNGLTGAKGAAGLPGVAGAPGLPGPRGLPGPVGAAGATG

ARGLVGEPGPAGSKGESGNKGEPGSAGPQGPPGPSGEEGKRGSTGEPGSAGPPGPPGLRG

SPGSRGLPGADGRAGVMGPPGSRGSTGPAGVRGPNGDSGRPGEPGLVGPRGLPGSPGNVG

PAGKEGPVGLPGIDGRPGPIGPAGARGEPGNIGFPGPKGPSGDAGKSGDKGHPGLAGARG

APGPDGNNGAQGPPGPQGVQGGKGEQGPAGPPGFQGLPGPSGPAGEVGKPGERGLPGEFG

LPGPAGARGERGPPGESGAAGPPGPIGSRGPSGPPGPDGNKGEPGAVGAPGNAGASGPGG

LPGERGAAGIPGGKGEKGETGLRGEVGNPGRDGARGAPGAVGAPGPAGATGDRGEAGAAG

PAGPAGPRGSPGERGEVGPAGPNGFAGPAGAAGQPGAKGERGTKGPKGENGVVGPTGPVG

AAGPSGPNGPPGPVGGRGDGGPPGMTGFPGAAGRTGPPGPSGITGPPGPPGAAGKEGLRG

PRGDQGPVGRTGEPGAAGPPGFAGEKGPSGEAGTAGPPGTPGPQGLLGPPGILGLPGTRG

ERGLPGVAGALGEPGPLGVAGPPGARGPPGAVGSPGVNGAPGEAGRDGNPGSDGPPGRDG

QPGHKGERGYPGNAGPAGAAGAPGPQGSVGPTGKHGNRGEPGPAGSVGPVGAVGPRGPSG

PQGIRGDKGEPGDKGPRGLPGLKGHNGLQGLPGLAGQHGDQGAPGAVGPAGPRGPAGPTG

PAGKDGRSGHPGTVGPAGVRGSQGSQGPAGPPGPPGPPGPPGASGGGYDFGYDGDFYRA

>SARCOPHILUS

QMSYGYDEKSGGGMSVPGPMGPSGPRGLPGPPGSPGPQGFQGPPGEPGEPGASGPMGPRG

PAGPPGKNGDDGEAGKPGRPGERGPPGPQGARGLPGTAGLPGMKGHRGFSGLDGAKGDSG

PAGPKGEPGSPGENGAPGQMGPRGLPGERGRPGPPGPAGARGNDGATGAAGPPGPTGPAG

PPGFPGAVGAKGEAGPQGSRGSEGPQGVRGEPGPPGPAGSPGPSGNPGADGQPGAKGANG

APGIAGAPGFPGARGPSGPQGPSGAPGPKGNSGEPGTPGNKGDPGAKGEPGPVGVQGPPG

PAGEEGKRGSRGEPGPAGLPGPAGERGGPGSRGFPGADGVAGPKGAPGERGAPGPAGPKG

SPGESGRPGEAGLPGAKGLTGSPGSPGPDGKTGPPGPAGQDGRPGPPGPPGARGQAGVMG

FPGPKGAAGEPGKAGERGVPGPPGAVGPAGKDGEAGAQGAPGPAGPAGERGEQGPAGSPG

FQGLPGPAGPPGEAGKPGEQGVPGDAGAPGPSGARGERGFPGERGVQGPPGPQGPRGANG

APGNDGAKGDAGAPGAPGGQGPPGLQGMPGERGAAGLPGAKGDRGDAGPKGADGAPGKDG

VRGLTGPIGPPGPAGPSGDKGESGPSGPAGPTGARGAPGERGEPGPPGPAGFAGPPGADG

QPGAKGEPGDAGAKGDAGPPGPAGPTGAPGPAGNVGAPGPKGARGSAGPPGATGFPGAAG

RVGPPGPSGNAGPPGPPGPAGKEGGKGPRGETGPIGRPGEVGPPGPPGPSGEKGSPGADG

PAGAPGTPGPQGIAGQRGVVGLPGQRGERGFPGLPGPSGEPGKQGPSGVSGERGPPGPAG

PPGLAGPPGESGREGSPGAEGSPGRDGAPGPKGDRGETGPAGPPGAPGAPGAPGPVGPAG

KAGDRGETGPSGPAGPAGPTGARGPAGPQGPRGDKGETGEQGDRGMKGHRGFSGLQGPPG

PPGSPGEQGPSGASGPAGPRGPPGSAGAAGKDGLNGLPGPIGPPGPRGRTGDAGPAGPPG

PPGPPGPPGPPSGGFDFSFLPQPPQEKAHDSGRYYRARQYDA-KGIDMGPGPMGLMGPRG

PPGASGPPGAQGFQGPAGEPGEPGQTGPAGARGPPGPPGKSGEDGHPGKPGRPGERGIVG

PQGARGFPGTPGLPGFKGIRGHNGLDGLKGQAGAPGVKGEPGAPGENGTPGQAGARGLPG

ERGRIGGPGPAGARGSDGSVGPVGPAXXXXXXGPPGFPGAPGPKGELGPVGNPGPAGPAG

PRGELGLPGMTGPVGPAGNPGANGLTGAKGAAGLPGVAGAPGLPGPRGIPGPAGAAGASG

PRGLAGEPGPAGSKGESGNKGEPGSAGPQGPPGPNGEEGKRGPNGEPGSTGPMGPPGLRG

VPGSRGLPGADGRAGGMGPPGNRGPSGPAGARGPNGDAGRPGEPGLMGPRGLPGSPGNVG

PTGKEGPAGLPGIDGRPGPTGPAGNRGEPGNIGFPGPKGPNGDPGKAGEKGHAGLAGARG

APGPDGNNGAQGPPGPTGVQGGKGEQGPAGPPGFQGLPGPSGPAGEGGKVGERGLPGEFG

LPGPAGPRGERGPPGESGAVGPTGSIGSRGPSGPPGPDGNKGEPGVVGAPGNAGPAGSGG

VPGERGAAGVPGGKGEKGETGPRGEFGNPGRDGARGAPGAMGAPGPAGATGERGEAGPAG

PVGPTGNRGAPGDRGEAGPAGPNGFAGPPGAAGQAGAKGERGTKGPKGENGIVGPTGPVG

AAGPAGPNGPPGPVGGRGDGGPPXXXXXXXXXXXXXXXXXXXXXGMTGFPGAAGKEGPRG

PRGDQGPLGRAGETGAVGPPGFAGEKGPPGEAGASGPPGSSGPQGLLGAPGILGLPGSRG

ERGLPGVSGSLGEPGPLGISGPPGARGPPGAVGSPGVNGAPGEAGRDGNPGNDGPPGRDG

LAGHKGERGYPGNPGAVGNAGAPGPHGTVGPAGKPGNRGEPGPVGSVGPVGPFGARGPSG

PQGPRGDKGEVGDKGPRGMNGFKGHNGFQGLPGLSGQHGDQGAPGSTGPAGPRGPAGPSG

PPGKDGRPGHAGAVGPAGIRGSQGSQGPAGPPGPPGLPGPPGPSGGGYDFGYEGDFYRA

>CHOLOEPUS_ENSEMBL_COL1A2

XXXX?XXXXXXXXXXXXXXXXXXXXXXXXXXXXXXXXXXXXXXXXXXXXXXXXXXXXXXXXXXXXXXXXXXXXXXXXXXXXXXXGARGFPGTPGLPGFKGIRGYNGLDGLKGQPGAAGVKGEPGAPGENGTPGQTGARGLPGERGRVGAPGPAGSRGSDGSVGPVGPAGPIGSAGPPGFPGAPGPKGELGPVGNTGPSGPAGPRGEQGLPGVSGPVGPPGNPGANGLTGAKGAAGLPGVAGAPGLPGPRGIPGPVGASGATGARGLVGEPGPAGSKGESGGKGEPGSAGPQGPPGSSGEEGKRGPSGESGSTGPTGPPGLRGGPGSRGLPGADGRAGVIGPAGARGASGPAGVRGPSGDTGRPGEPGLMGARGLPGSPGNVGPAGKEGPAGLPGIDGRPGPIGPAGARGEAGNIGFPGPKXXXXXXXXXXXXGHAGLAGNRGAPGPDGNNGAQGPPGLQGVQGGKXXXXXXXXXXXXXXXXXXXXXXXXXXXXXXXXXXXXXXXXXXXXXGERGPPGESGAVGPSGAIGSRGPSGPPGPDGNKGEPGVVGAPGTAGPAGSGGLPGERGAAGIPGGKGEKGETGLRGEVGTTGRDGARXXXXXXXXXXXXXXXXXXGEAGAAGPAGPAGPRGSPGERGEVGPAGPNGFAGPAGAAGQPGAKGERGTKGPKGENGIVGPTGPVGSAGPAGPNGPAGPAGSRGDGGPPGVTGFPGAAGRTGPPGPXXXXXXXXXXXXXXXXXXXXXXXXXXXXXXXXXXXXXXXXXXXXXXXXXGEPGTAGPPGTAGPQGLLGAPGILGLPGSRGERGLPGVAGAVXXXXXXXXXXXXXXXXXXXXXXXXXXXXXXXXXXXXGNPGSDGPPGRDGLPGHKGERGYAGNPGPVGAAGAPGPHGAVGPAGKHGNRGEPGPVGSAXXXXXXXXXXXXGPQGIRGDKGEPGDKGPRGLPGLKGHNGLQGLPGLAGQHGDQGAPGPVGPAGPRGPSGPSGPPGKDGRTGHPGAVGPAGIRGSQGSQGPSGPPGPPGPPGPPGASGGGYDFGYEGDFYRA
