# Supplementary figures and images for: Collagen Sequence Analysis of the Extinct Giant Ground Sloths Lestodon and Megatherium
Source: PLoS One. 2015 Nov 5;10(11):e0139611. doi: 10.1371/journal.pone.0139611 (PMC4634953; doi:10.1371/journal.pone.0139611)

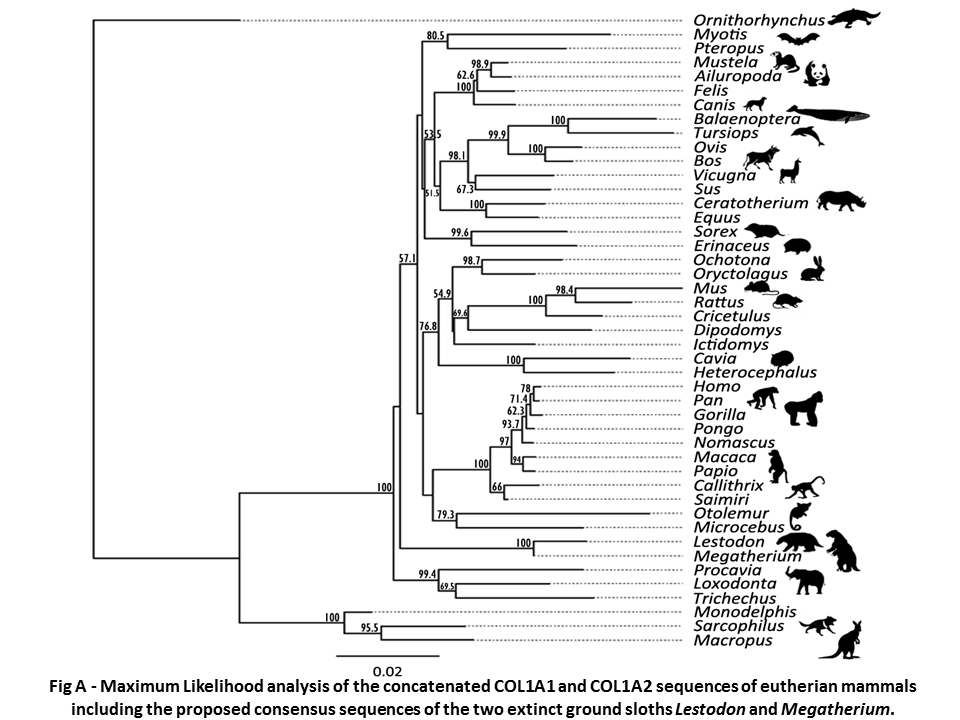

Supplement: S3 File — Supplementary figures (Figures A-P) including additional Maximum Likelihood phylogenetic tree (Figure A) and tandem mass spectra for new unique peptide sequences (Figures B-P). (ZIP) [file pone.0139611.s003.zip › S3 File/Slide1.TIF]

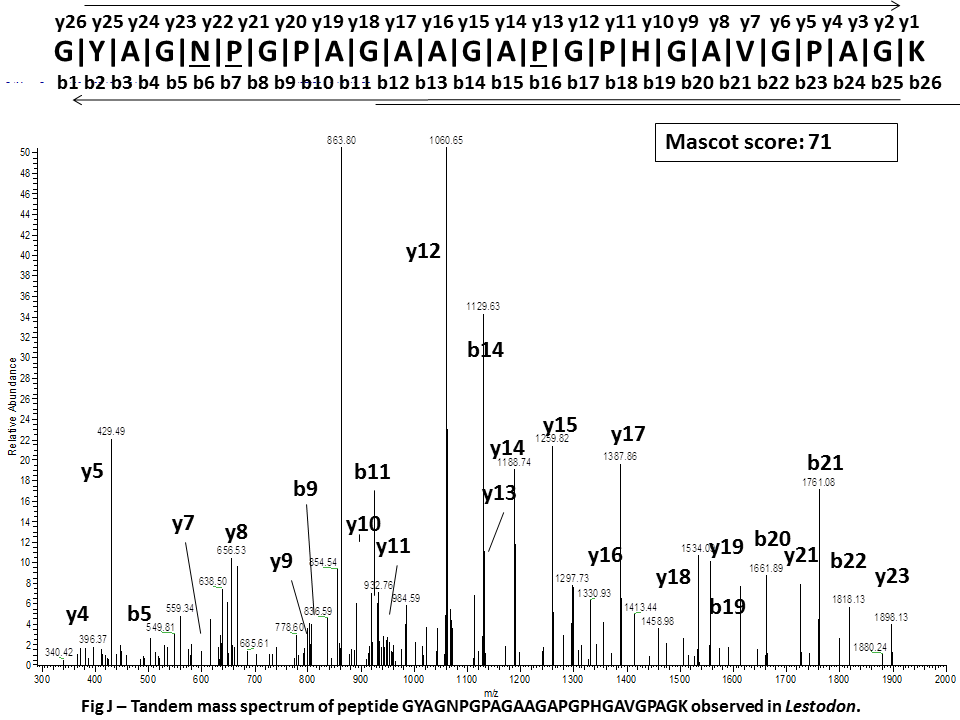

Supplement: S3 File — Supplementary figures (Figures A-P) including additional Maximum Likelihood phylogenetic tree (Figure A) and tandem mass spectra for new unique peptide sequences (Figures B-P). (ZIP) [file pone.0139611.s003.zip › S3 File/Slide10.TIF]

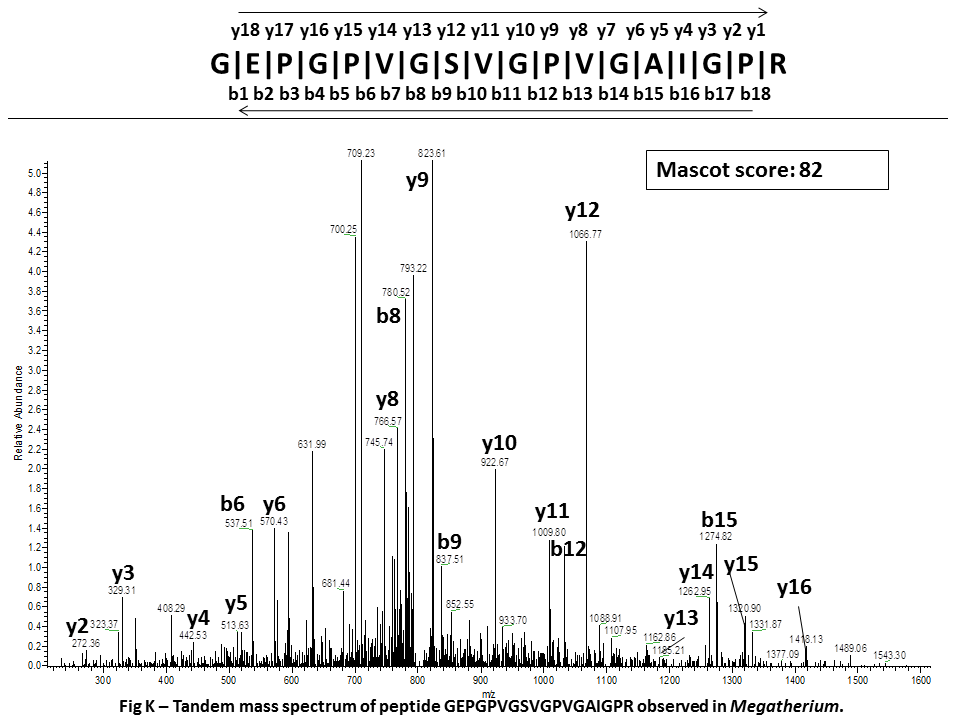

Supplement: S3 File — Supplementary figures (Figures A-P) including additional Maximum Likelihood phylogenetic tree (Figure A) and tandem mass spectra for new unique peptide sequences (Figures B-P). (ZIP) [file pone.0139611.s003.zip › S3 File/Slide11.TIF]

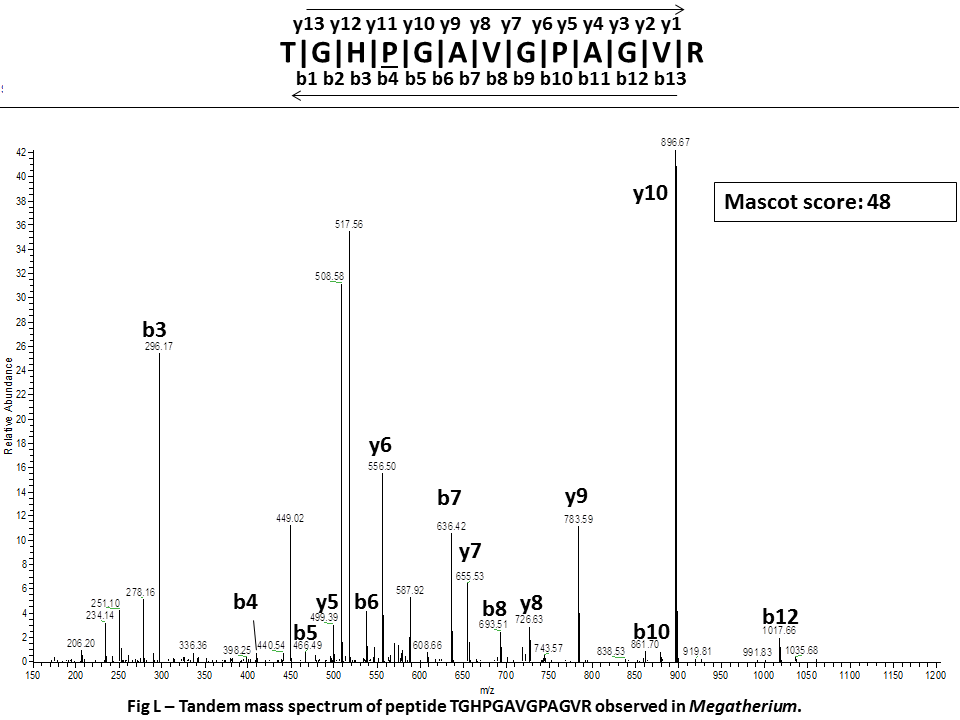

Supplement: S3 File — Supplementary figures (Figures A-P) including additional Maximum Likelihood phylogenetic tree (Figure A) and tandem mass spectra for new unique peptide sequences (Figures B-P). (ZIP) [file pone.0139611.s003.zip › S3 File/Slide12.TIF]

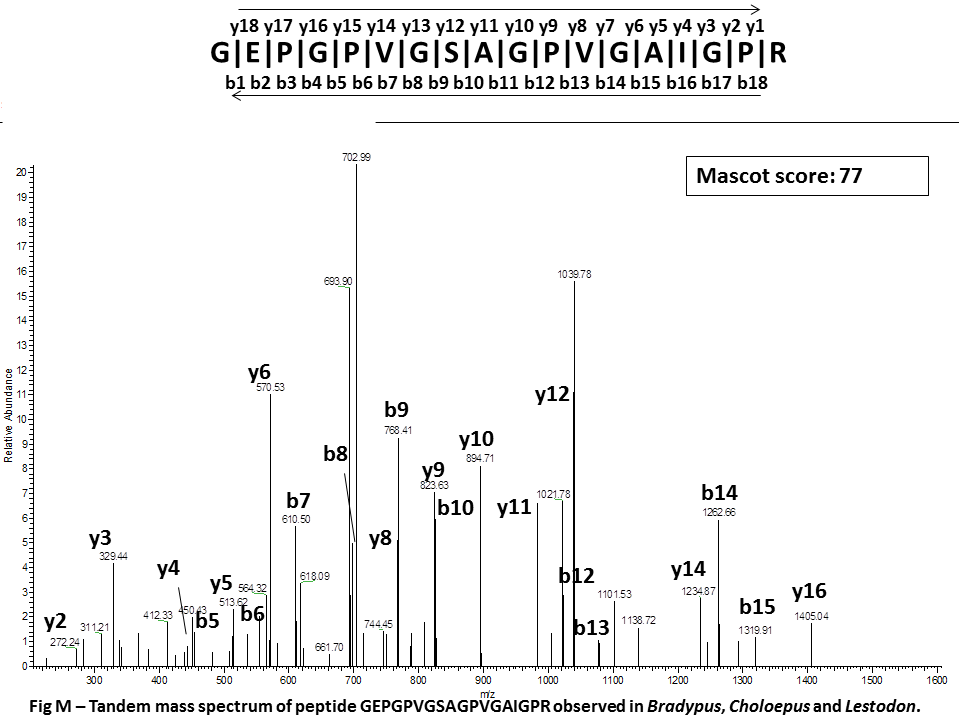

Supplement: S3 File — Supplementary figures (Figures A-P) including additional Maximum Likelihood phylogenetic tree (Figure A) and tandem mass spectra for new unique peptide sequences (Figures B-P). (ZIP) [file pone.0139611.s003.zip › S3 File/Slide13.TIF]

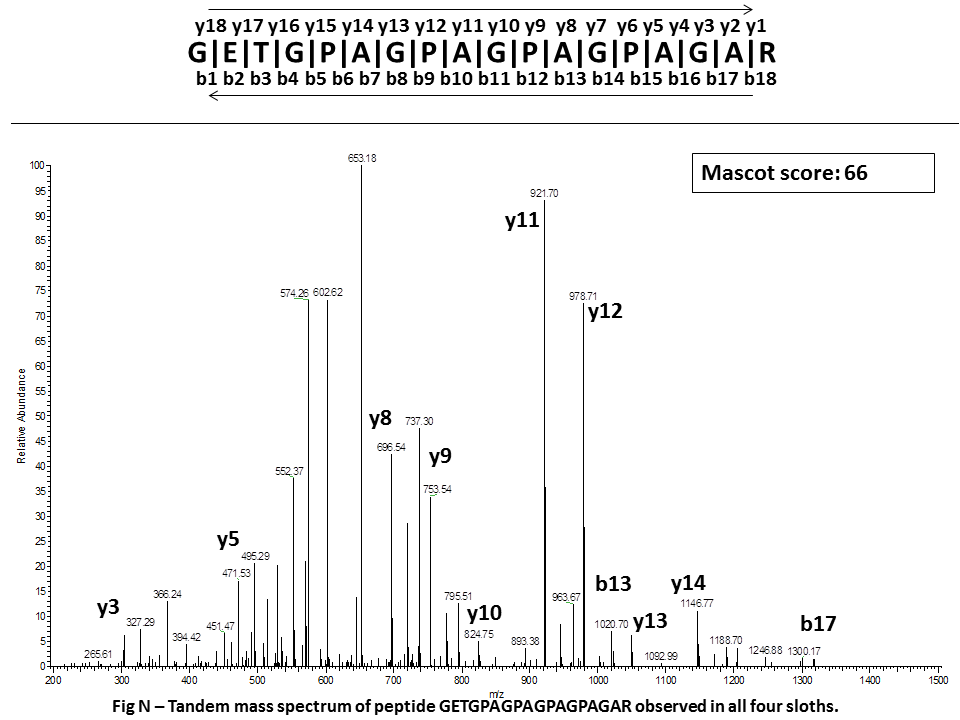

Supplement: S3 File — Supplementary figures (Figures A-P) including additional Maximum Likelihood phylogenetic tree (Figure A) and tandem mass spectra for new unique peptide sequences (Figures B-P). (ZIP) [file pone.0139611.s003.zip › S3 File/Slide14.TIF]

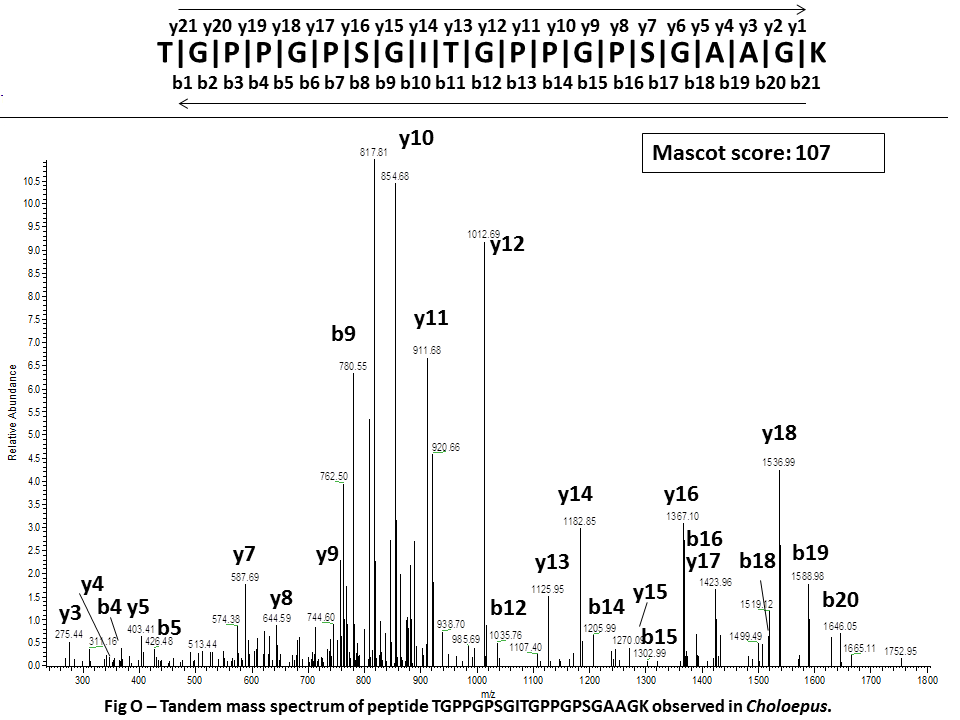

Supplement: S3 File — Supplementary figures (Figures A-P) including additional Maximum Likelihood phylogenetic tree (Figure A) and tandem mass spectra for new unique peptide sequences (Figures B-P). (ZIP) [file pone.0139611.s003.zip › S3 File/Slide15.TIF]

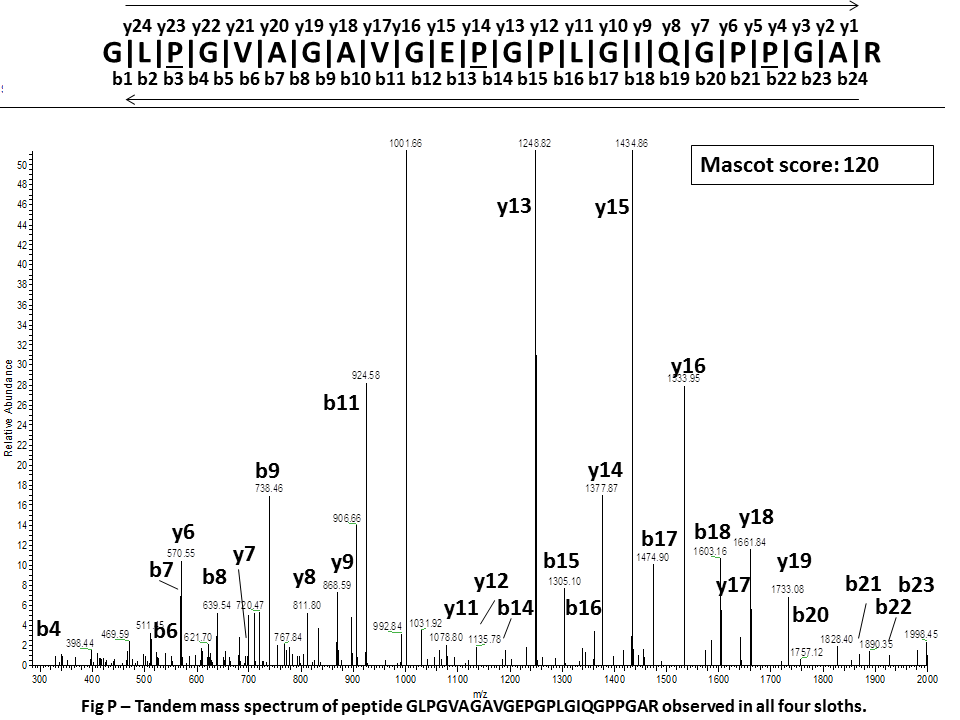

Supplement: S3 File — Supplementary figures (Figures A-P) including additional Maximum Likelihood phylogenetic tree (Figure A) and tandem mass spectra for new unique peptide sequences (Figures B-P). (ZIP) [file pone.0139611.s003.zip › S3 File/Slide16.TIF]

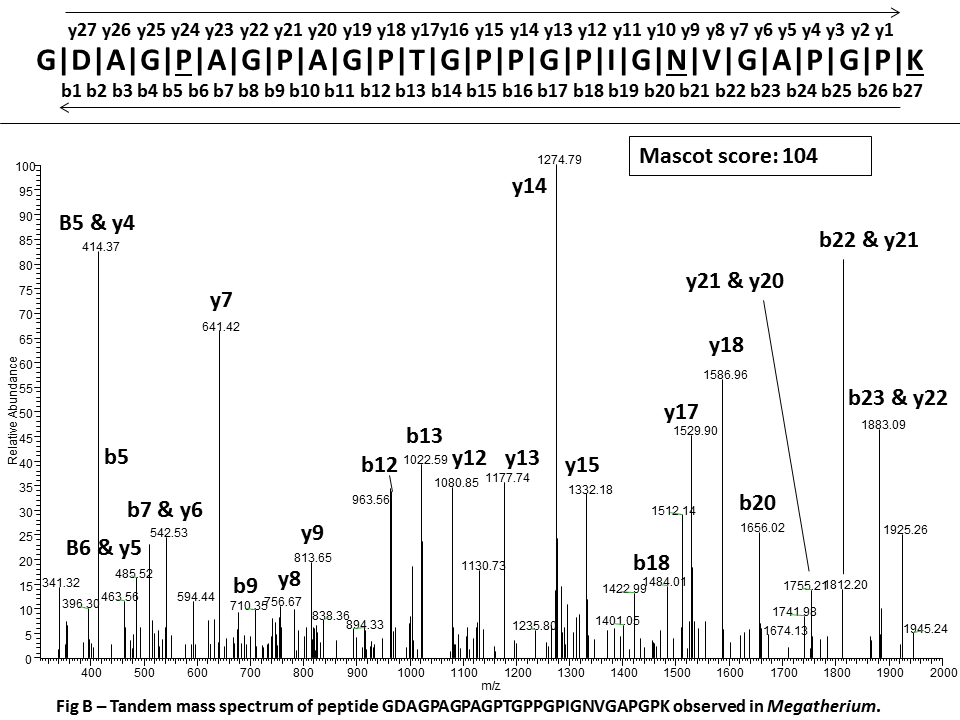

Supplement: S3 File — Supplementary figures (Figures A-P) including additional Maximum Likelihood phylogenetic tree (Figure A) and tandem mass spectra for new unique peptide sequences (Figures B-P). (ZIP) [file pone.0139611.s003.zip › S3 File/Slide2.TIF]

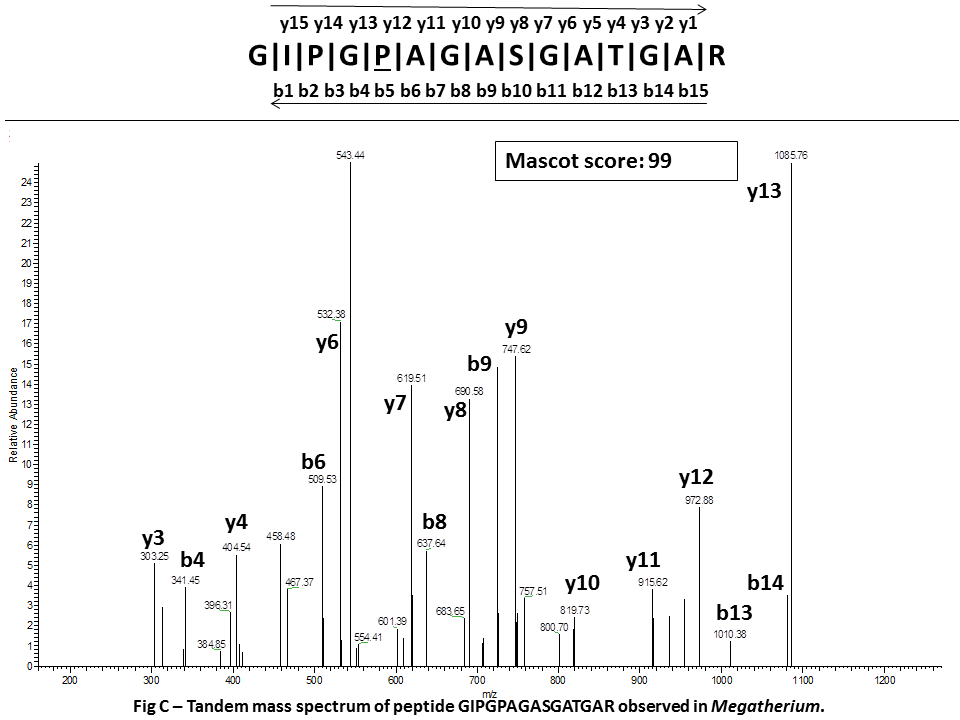

Supplement: S3 File — Supplementary figures (Figures A-P) including additional Maximum Likelihood phylogenetic tree (Figure A) and tandem mass spectra for new unique peptide sequences (Figures B-P). (ZIP) [file pone.0139611.s003.zip › S3 File/Slide3.TIF]

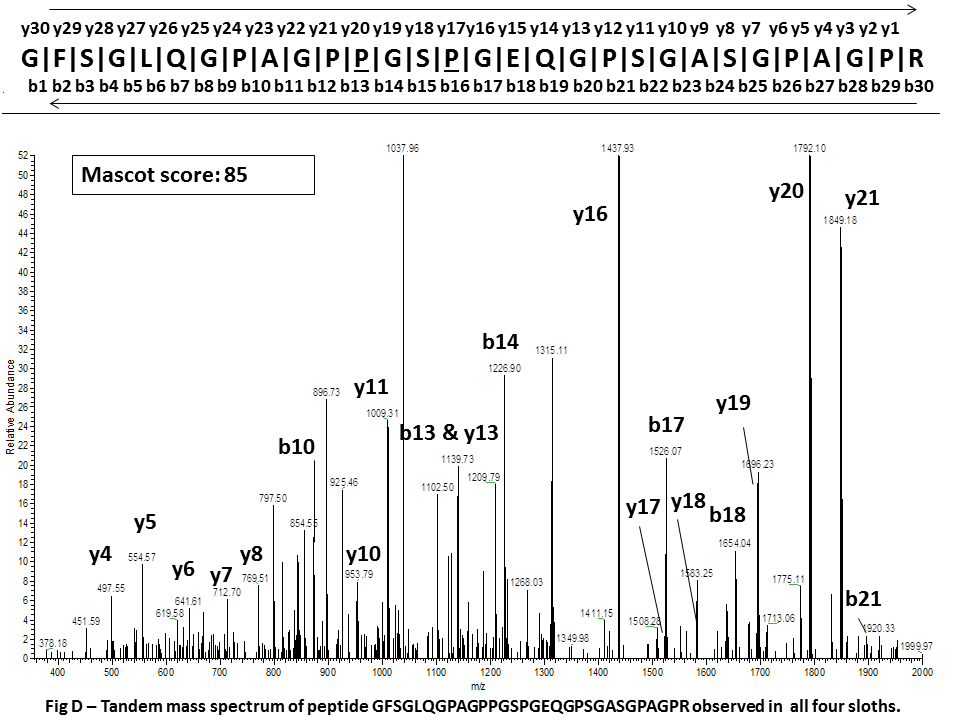

Supplement: S3 File — Supplementary figures (Figures A-P) including additional Maximum Likelihood phylogenetic tree (Figure A) and tandem mass spectra for new unique peptide sequences (Figures B-P). (ZIP) [file pone.0139611.s003.zip › S3 File/Slide4.TIF]

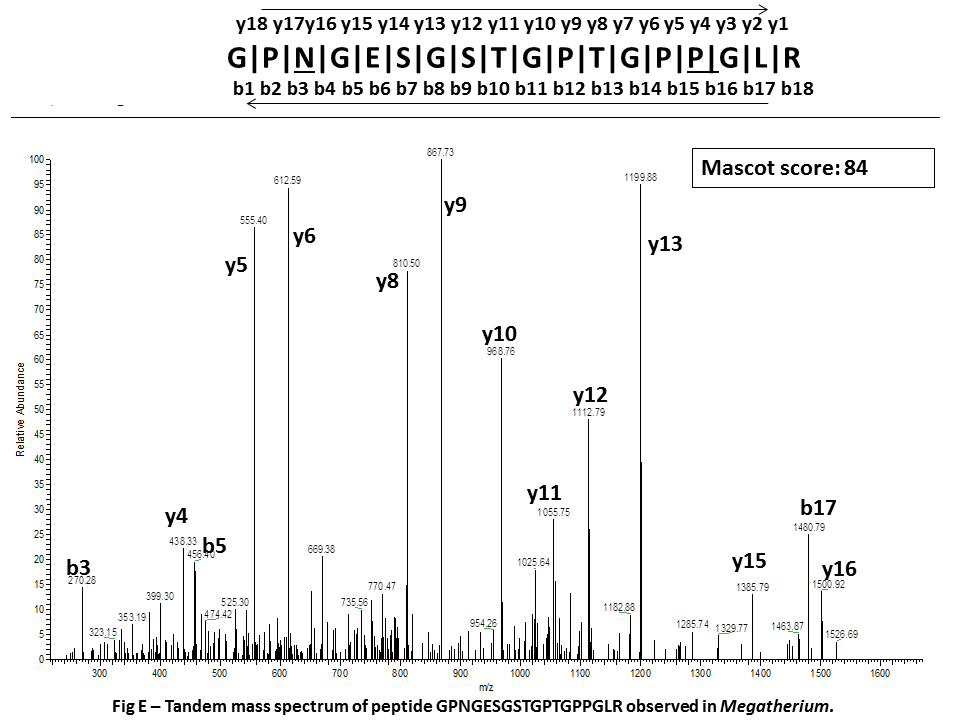

Supplement: S3 File — Supplementary figures (Figures A-P) including additional Maximum Likelihood phylogenetic tree (Figure A) and tandem mass spectra for new unique peptide sequences (Figures B-P). (ZIP) [file pone.0139611.s003.zip › S3 File/Slide5.TIF]

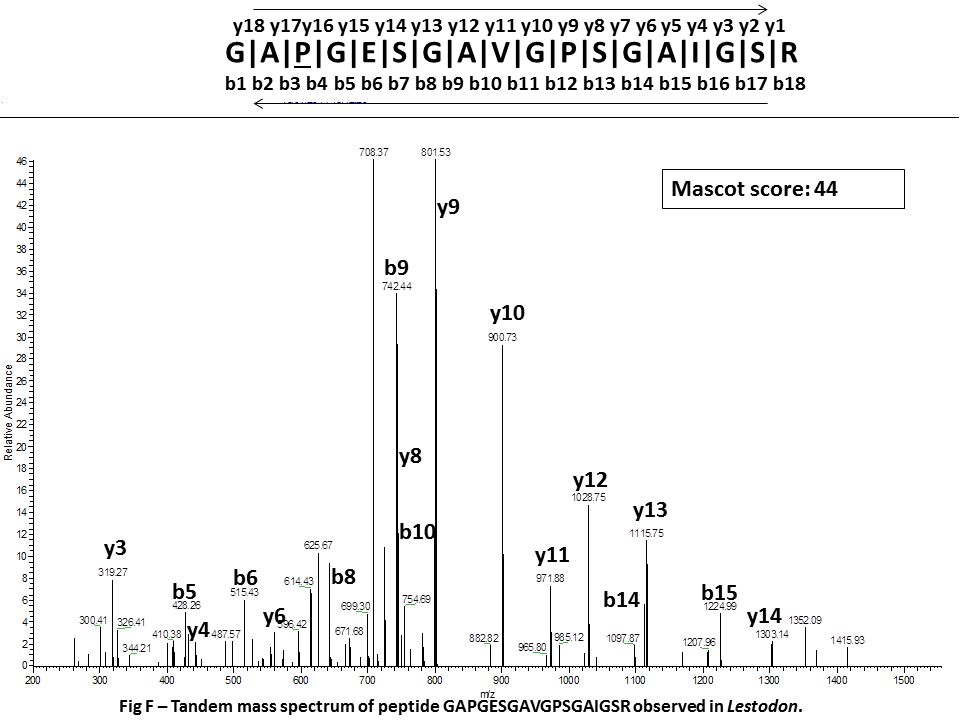

Supplement: S3 File — Supplementary figures (Figures A-P) including additional Maximum Likelihood phylogenetic tree (Figure A) and tandem mass spectra for new unique peptide sequences (Figures B-P). (ZIP) [file pone.0139611.s003.zip › S3 File/Slide6.TIF]

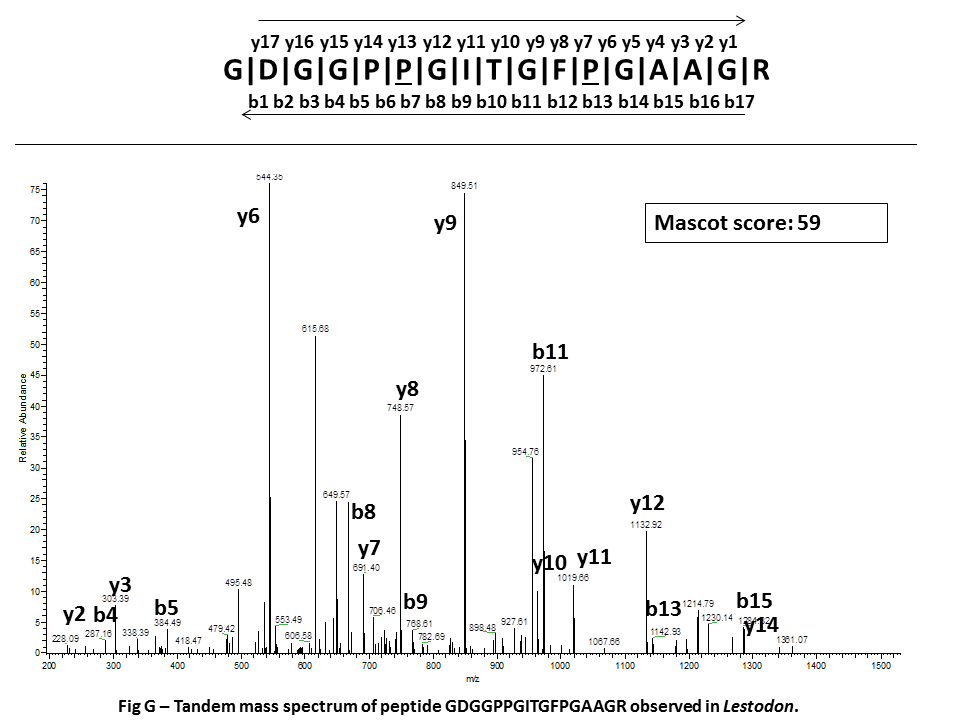

Supplement: S3 File — Supplementary figures (Figures A-P) including additional Maximum Likelihood phylogenetic tree (Figure A) and tandem mass spectra for new unique peptide sequences (Figures B-P). (ZIP) [file pone.0139611.s003.zip › S3 File/Slide7.TIF]

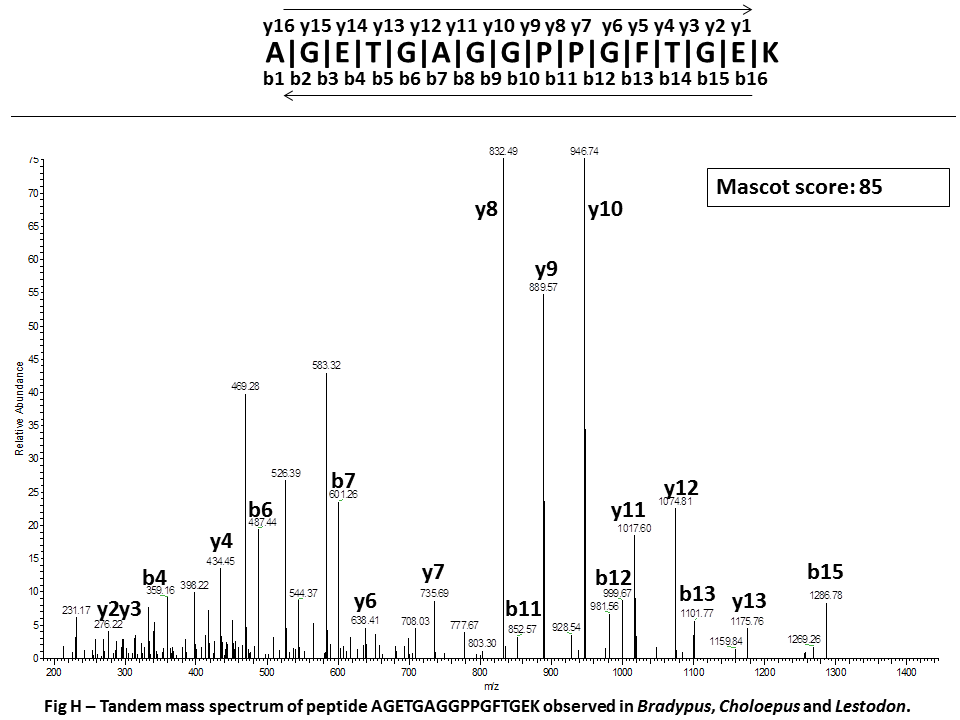

Supplement: S3 File — Supplementary figures (Figures A-P) including additional Maximum Likelihood phylogenetic tree (Figure A) and tandem mass spectra for new unique peptide sequences (Figures B-P). (ZIP) [file pone.0139611.s003.zip › S3 File/Slide8.TIF]

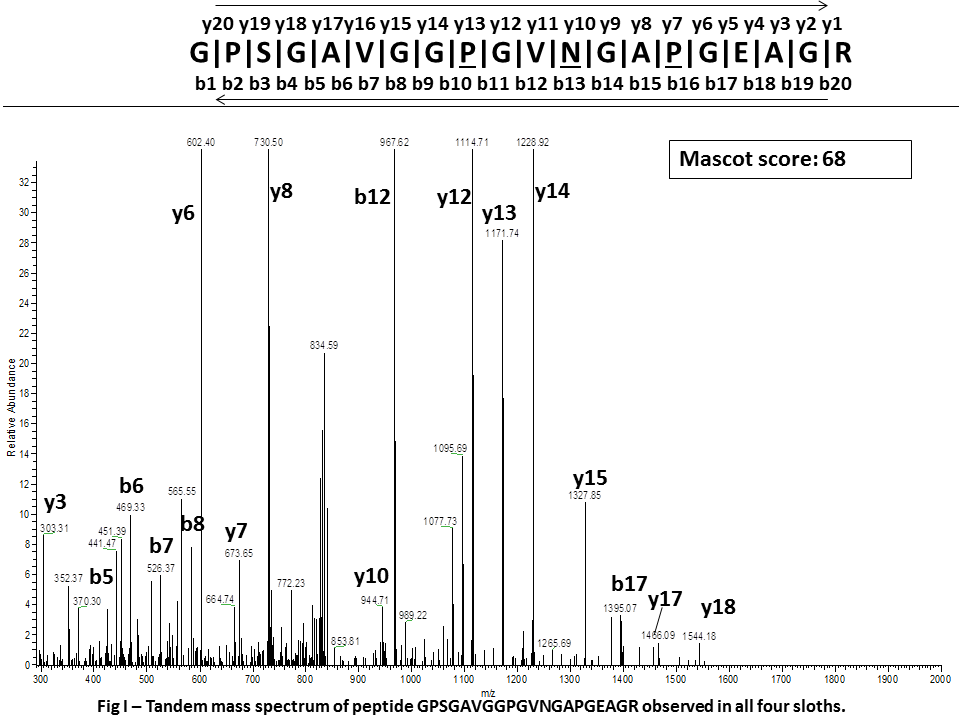

Supplement: S3 File — Supplementary figures (Figures A-P) including additional Maximum Likelihood phylogenetic tree (Figure A) and tandem mass spectra for new unique peptide sequences (Figures B-P). (ZIP) [file pone.0139611.s003.zip › S3 File/Slide9.TIF]
